# Protodefluorinated Selectfluor^®^ Aggregatively Activates Selectfluor^®^ for Efficient Radical C(sp^3^)−H Fluorination Reactions

**DOI:** 10.1002/cssc.202401057

**Published:** 2024-10-30

**Authors:** Shahboz Yakubov, Bastian Dauth, Willibald J. Stockerl, Wagner da Silva, Ruth M. Gschwind, Joshua P. Barham

**Affiliations:** ^1^ Institute of Organic Chemistry University of Regensburg Universitätsstr. 31 93053 Regensburg Germany

**Keywords:** fluorination, aggregation, radical reactions, photocatalysis, protodefluorinated Selectfluor®

## Abstract

Efficient fluorination reactions are key in the late‐stage functionalization of complex molecules in medicinal chemistry, in upgrading chemical feedstocks, and in materials science. Radical C(sp^3^)−H fluorinations using Selectfluor^®^ – one of the most popular fluorination agents – allow to directly engage unactivated precursors under mild photochemical or thermal catalytic conditions. However, **H−TEDA(BF_4_)_2_** to date is overlooked and discarded as waste, despite comprising 95% of the molecular weight of Selectfluor^®^. We demonstrate that the addition of **H−TEDA(BF_4_)_2_** at the start of fluorination reactions markedly promotes their rates and accesses higher overall yields of fluorinated products (~3.3 × higher on average across the cases studied) than unpromoted reactions. Several case studies showcase generality of the promotor, for photochemical, photocatalytic and thermal radical fluorination reactions. Detailed mechanistic investigations reveal the key importance of aggregation changes in Selectfluor^®^ and **H−TEDA(BF_4_)_2_** to fill gaps of understanding in how radical C(sp^3^)−H fluorination reactions work. This study exemplifies an overlooked reaction waste product being upcycled for a useful application.

## Graphical abstract

**H‐TEDA(BF_4_)_2_** is an overlooked waste product in radical C‐H fluorinations using Selectfluor^®^. We report the hitherto ‘hidden’ role of **H‐TEDA(BF_4_)_2_** as a promotor. When added at the beginning of radical C‐H fluorinations, rates and final product yields are dramatically promoted. DOSY NMR indicates a reactive heteroaggregate between Selectfluor^®^ and **H‐TEDA(BF_4_)_2_** to explain rate enhancement. The promotor is recoverable and reusable.

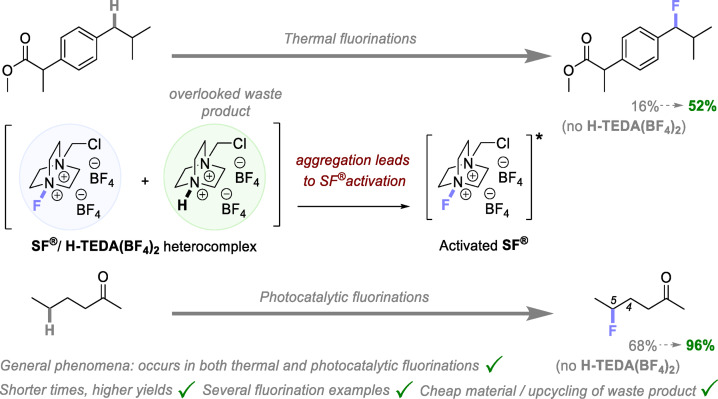

## Introduction

**Scheme 1:**
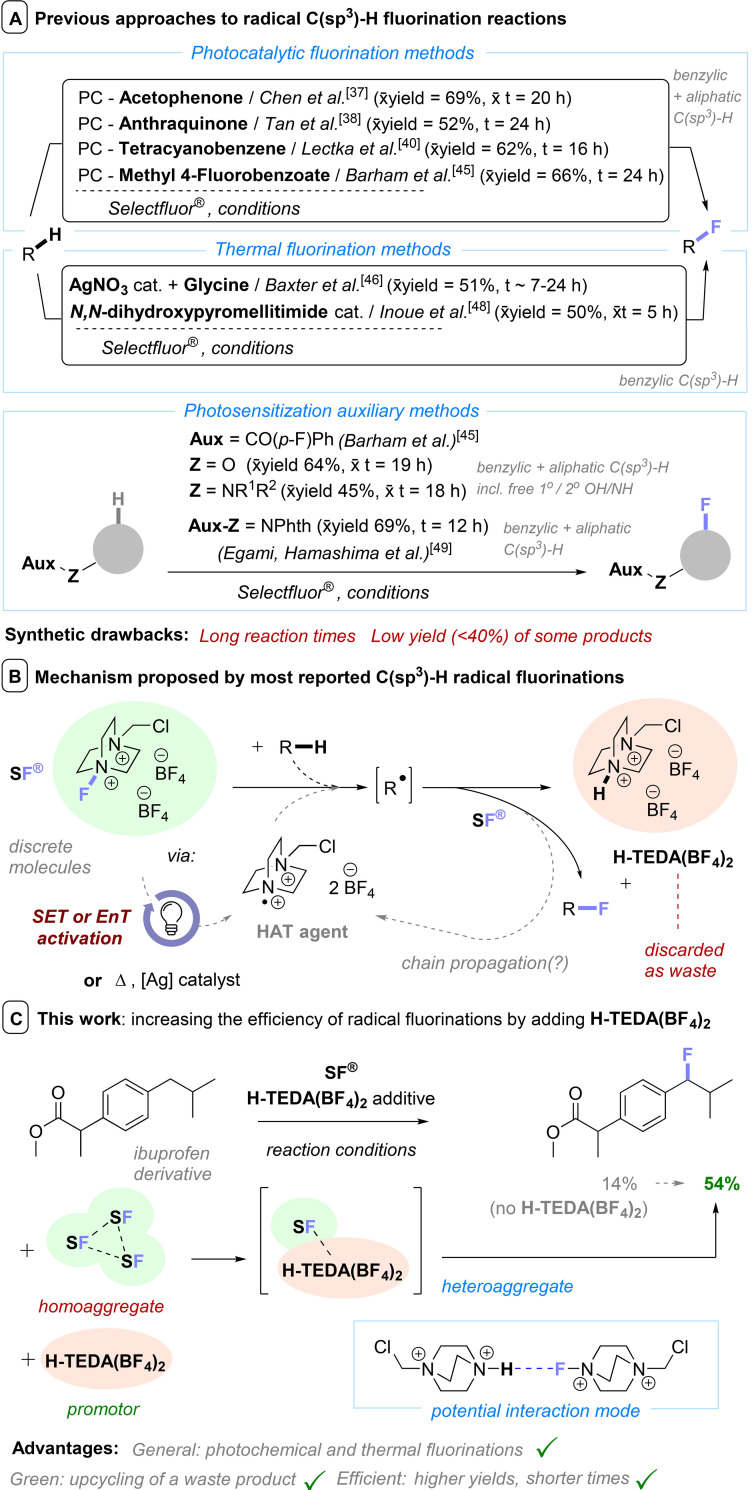
(A) Previous approaches to radical fluorination reactions. x̄=mean average of yield (^1^H NMR by default) /reaction time of the scope reported. (B) General mechanistic dogma proposed in most reported radical fluorination methods. (C) Increasing radical fluorination efficiencies by adding promotor **H‐TEDA(BF_4_)_2_** to form the active **SF^®^**/**H‐TEDA(BF_4_)_2_** heteroaggregate.

Apart from well‐defined complexes and single molecules, a particularly useful form of matter is an aggregate. Aggregates (i. e., irregular clusters of many molecules) demonstrate modified or wholly new properties in comparison to their molecular components. The profound effects of aggregation on the photophysics of organic molecules are well studied,[^1^, ^2^, ^3^, ^4^] with the importance of their aggregation states in catalysis receiving increasing attention.[^5^, ^6^, ^7^, ^8^, ^9^, ^10^, ^11^, ^12^, ^13^, ^14^, ^15^, ^16^, ^17^] However, the roles of reactant aggregation states in photochemical or thermal chemical reactions are underestimated.^[18]^ This is primarily due to the difficulties in detecting aggregation states in solution that are not obvious by UV‐visible spectroscopy. Nonetheless, aggregation states of reactants influence solubility, reactivity, selectivity and efficiency of their reactions. Elsewhere, the importance of organofluorine compounds to all areas of chemistry has exploded over the past decades, including organic synthesis,[^19^, ^20^, ^21^, ^22^, ^23^] pharmaceutical science,[^24^, ^25^, ^26^, ^27^] and materials development.[^28^, ^29^, ^30^] In this context, procedures for the direct conversion of unactivated C−H bonds to C−F bonds under mild conditions are highly prized. Among these, radical C(sp^3^)−H fluorinations[^31^, ^32^, ^33^] using Selectfluor^®^ (**F−TEDA(BF_4_)_2_**_,_ **‘SF^®^’**) are particularly attractive for their applicability in late‐stage functionalization (LSF) of complex molecules, their mild conditions, and (when photosensitized) the use of light as a sustainable source of energy.[^34^, ^35^] Activation of **SF^®^** can be achieved by a photocatalyst, a photosensitized auxiliary or thermal fluorination methods often in the presence of transition metal (T.M.) catalysts (Scheme 1, A).[^36^, ^37^, ^38^, ^39^, ^40^, ^41^, ^42^, ^43^, ^44^, ^45^, ^46^, ^47^, ^48^, ^49^] However, despite good or excellent yields for some products (average yields ~45–65% throughout previous reports), there is still room for improvement for many other products which arise in unsatisfactory yields (<40%).

In addition, long reaction times are oftentimes required (typically >12 h for photochemical reactions). The generally accepted mechanism that is proposed in most reported radical fluorination methods involves HAT between the substrate and **TEDA^•2+^** to generate the radical of the substrate (Scheme 1, C). In many previous reports on radical C−H fluorinations, a chain mechanism is drawn and inferred. To the contrary, Lu, Soo, Tan and co‐workers^[39]^ measured a very low quantum yield for their photocatalytic C(sp^3^)−H fluorinations. Later, Baxter^[46]^ also contested a possibility of radical chain mechanism after showing how stoichiometric (and not catalytic) amounts of glycine were necessary for product formation. Baxter also demonstrated the generation of fluoride during reactions,^[46]^ revealing how **SF^®^** can engage in unproductive electron transfer reactions. Since **SF^®^** is the limiting agent, such pathways could explain the limited yields in these radical fluorination reactions. Owing to on‐line NMR irradiation capability, our team was able to discover an induction period for metal‐free photochemical C−H radical fluorination reactions^[45]^ – that is likely a general phenomenon for all photochemical C(sp^3^)−H fluorinations (see kinetic results *vide infra*). This suggests the mechanistic situation is more complex than previously anticipated. We hypothesized^[45]^ that this induction period relates to a change in the aggregation state of **SF^®^** and that this may be the key efficiency‐limiting factor in such reactions but the nature of the reactive aggregate and its composition remained unclear.

Protodefluorinated Selectfluor^®^ (**H‐TEDA(BF_4_)_2_**) and **TEDA(BF_4_)** are the byproducts of any fluorination reaction using **SF^®^** as a radical/electrophilic fluorine source (Scheme 1, B), and are always discarded as waste.[^50^, ^51^] According to a research excellence framework report on the institute in which it was discovered, **SF^®^** is the world′s most popular organic fluorination reagent in industrial processes – with annual worldwide production reaching ~25 tonnes (as of 2014).^[52]^ However, obviously with the transfer of only one fluorine atom to the product in fluorination reactions, 95% of the M.W. is discarded. This could generate as much as ~24 tonnes of **H‐TEDA(BF_4_)_2_**/**TEDA(BF_4_)** waste per year. If such waste could be upcycled for useful synthetic applications (even once), this would be a valuable endeavor.

Herein, we report the discovery of **H‐TEDA(BF_4_)_2_** ‘s hitherto unknown function as a cheap and fully recoverable, reusable promotor. Adding **H‐TEDA(BF_4_)_2_** improves the efficiencies of a diversity of radical fluorinations by altering the aggregation state of **SF^®^** (Scheme 1, C). Promoted reactions achieve yields up to 8× higher (on average 3.3× higher) then unpromoted ones. We exemplify promotion in both thermal and photocatalytic C(sp^3^)−H fluorinations where **H‐TEDA(BF_4_)_2_** markedly increases the efficiency, rapidity, and practicality of reactions. A highly attractive feature is that the **H‐TEDA(BF_4_)_2_** can either be authentically synthesized from cheap DABCO or isolated as a ‘waste’ product from radical fluorination reactions, both approaches being feasible, high yielding and tracelessly executed on a gram scale. The latter approach allows to upcycle a waste product that is until now discarded after radical fluorination reactions, improving the efficiency of those very reactions.

## Results and Discussion

### Discovering Radical Fluorination Promotor H‐TEDA(BF_4_)_2_

**Scheme 2:**
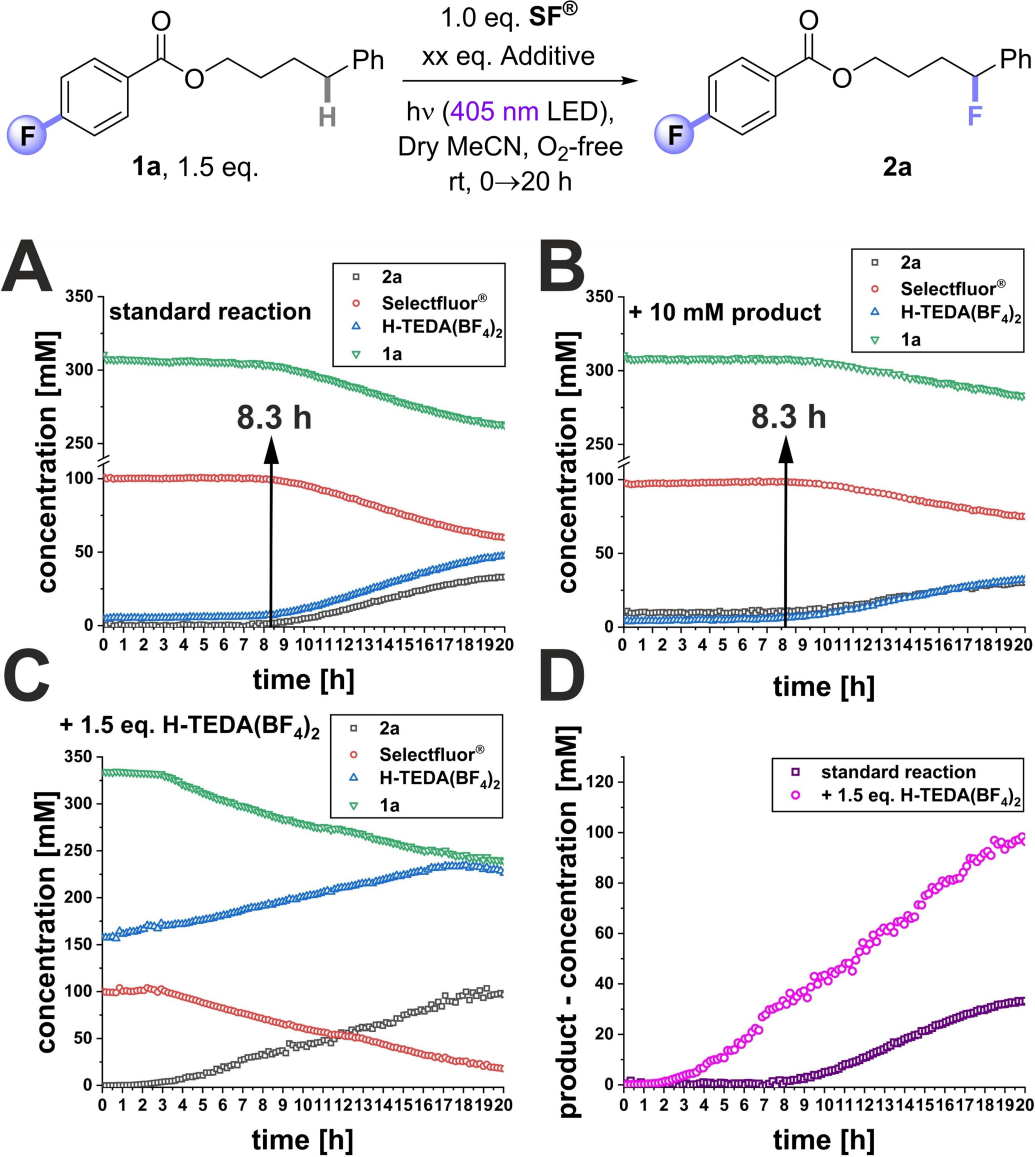
1.5 eq. **H‐TEDA(BF_4_)_2_** loading reveals a significantly shortened induction period and a threefold increase in product formation in the photochemical C(sp^3^)‐H fluorination. Kinetic profiles of the photochemical reaction (A) under standard conditions, (B) with 10 mM product added, and (C) with 1.5 eq. **H‐TEDA(BF_4_)_2_** at the start. D) Detailed comparison of the reaction profiles of (A) and (C).

In photosensitized fluorination reactions with **SF^®^** we observed unproductive induction periods of up to ~8 h in *in situ* NMR reactions, showing how/that **SF^®^** is initially in an inactive state. Previously, we could decrease this induction period by covalently attaching a photosensitizing auxiliary and increasing the substrate loading.^[45]^ However, it was clear that only an understanding of the key activation mode of **SF^®^** would allow us to develop a more efficient protocol for **SF^®^** fluorinations in general. With the kinetic profile of **1a** reminiscent of an autocatalytic reaction (see Scheme 2, A), we wondered if the reaction products were responsible for activating **SF^®^**. In the first instance, we focused our efforts towards exploring the effect of adding the fluorinated reaction product **2a** and **H‐TEDA(BF_4_)_2_** on the reaction kinetics. For this kinetic studies were performed by on‐line LED irradiation in the NMR spectrometer (see Supporting Information (SI) for details).[^53^, ^54^, ^55^, ^56^, ^57^, ^58^] Time‐resolved ^1^H{^19^F} NMR was used to track consumption of all starting materials and formation of all products. Addition of **2a** did not influence the induction period of ~8.3 h (Scheme 2, B). However, addition of 1.5 eq. of **H‐TEDA(BF_4_)_2_** led to a substantially shorter induction period of ~1.7 h and a profile typical of a first‐order reaction (Scheme 2, C). Moreover, the rate of **2a**‘s formation and its overall yield was notably higher (Scheme 2, D). Thus, it was a highly encouraging find that adding exogeneous **H‐TEDA(BF_4_)_2_** at the start of reactions not only strikingly shortens their induction period, it markedly improves the efficiency of the reactions vs nascent **H‐TEDA(BF_4_)_2_** generated in the reaction.^[59]^

This result raised the question of whether **H‐TEDA(BF_4_)_2_** is the missing key activation compound for **SF^®^** radical fluorinations in general. Therefore, to evaluate the generality and synthetic efficiency benefit of additive **H‐TEDA(BF_4_)_2_** we examined the impact of its presence on all three known reaction sub‐classes of radical C(sp^3^)−H fluorinations; photocatalytic, photochemical and thermal (**Reaction Classes 1–3**). We specifically selected substrates which afforded poor/moderate product yields (<20% or <55%) under the standard (unpromoted) reaction conditions. For all subsequent case studies, we evaluated the standard literature conditions without vs with 2.0 eq. of **H‐TEDA(BF_4_)_2_** additive present at the start of the reaction for a fixed reaction time period. Even if higher than 2.0 eq. of promotor gave higher yields in certain case studies, 2.0 eq. of promotor was used by default as it (i) was the most generally applicable loading and (ii) was a compromise of promotion effect vs increased mass intensity/screening of light from the reaction by particulates as solubility became problematic at higher loadings (see the SI file for results with different loadings). Finally, while other protic derivatives were also found to promote the reaction (see below), **H‐TEDA(BF_4_)_2_** was the most general promotor across case studies and as it is formed in the reaction this avoids contamination by foreign entities that may interfere with certain substrate functional groups.

#### Reaction Class 1: Photocatalytic C(sp^3^)−H Radical Fluorinations

**Scheme 3:**
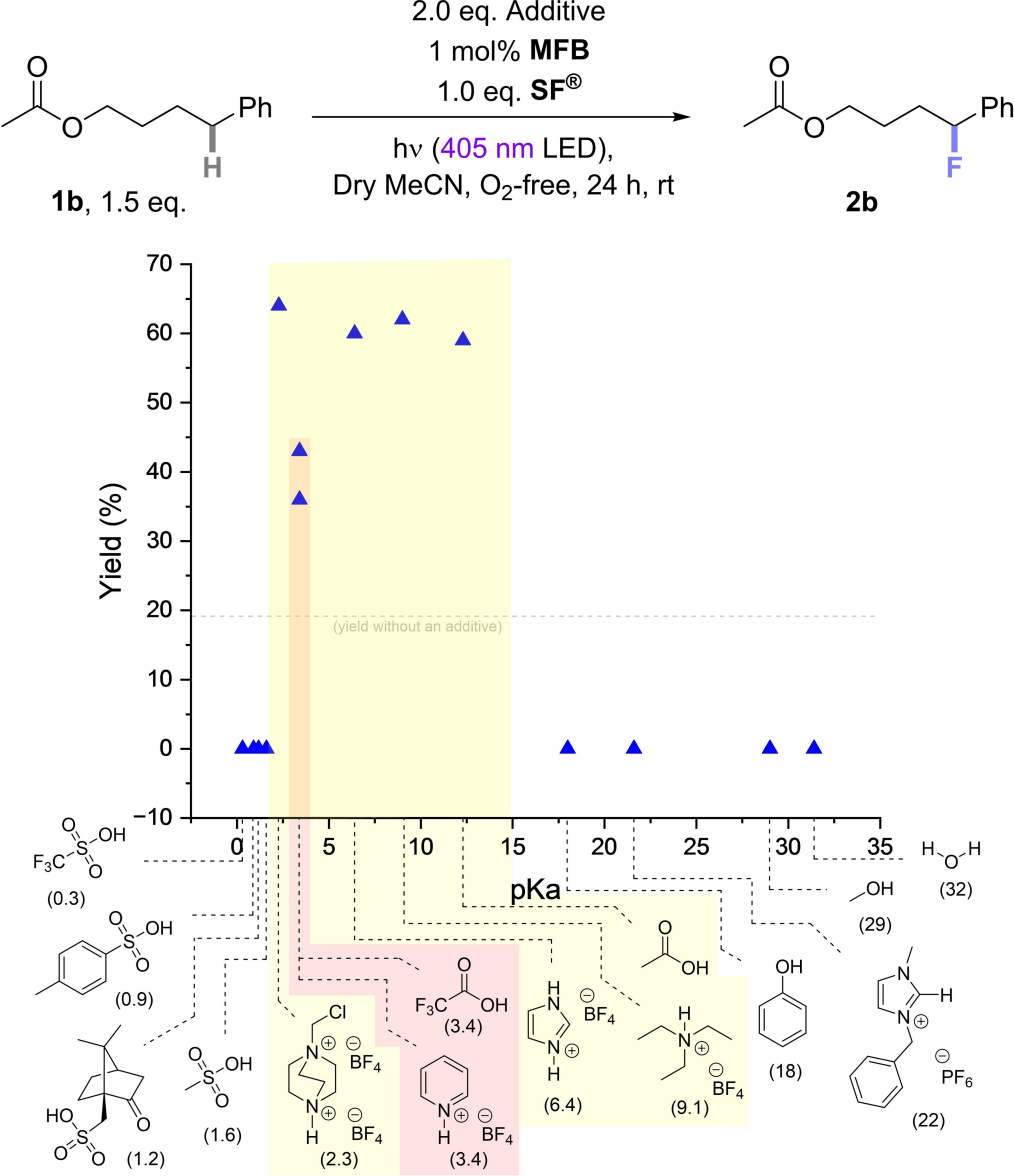
Photocatalytic fluorination of **1b** with **MFB** as the photocatalyst in presence of different additives and different loadings.

Case Study 1: Despite a lot of synthetic developments in radical fluorinations using **SF^®^** under various catalytic manifolds,[^36^, ^37^, ^38^, ^39^, ^40^, ^41^, ^42^, ^43^, ^44^, ^45^, ^46^, ^47^, ^48^, ^49^] still a lot of substrates with low to poor yields or requiring long reaction times remain. These reactions can be enabled by the addition of **H‐TEDA(BF_4_)_2_**. As a first test system we selected substrates for benzylic fluorination which reacted poorly. For poorly reacting molecules, activation via a simple additive would be by far more straightforward than a covalently bound photosensitizer.[^45^, ^49^] In our previous study,^[45]^ many substrates (unprotected alcohols or amines) required covalent installation of a 4‐fluorobenzoyl photosensitizing auxiliary to achieve satisfactory (>50%) yields. One poorly‐efficient substrate was 4‐phenylbutyl acetate (**1b**),^[45]^ which afforded only 19% yield of **2b** when treated with 1 mol% photocatalyst (**MFB**) under 400 nm irradiation for 24 h (Scheme 3, grey dotted line). Adding 2.0 eq. of promoter **H‐TEDA(BF_4_)_2_** to the reaction mixture increased the yield of **2b** by more than 3× from 19% to 64% (Scheme 3, highest blue triangle). The photocatalytic reaction efficiency of **1b** was then comparable to that achieved with the covalently attached 4‐fluorobenzoyl derivative (**1a**→**2a** (67%)), showing that a simple addition of **H‐TEDA(BF_4_)_2_** can substitute even a covalently bound photosensitization auxiliary.

We questioned if Brønsted acidity of **H‐TEDA(BF_4_)_2_** might underlie its promotionary role and examined other protic additives (see the SI file for full details). Since the p*K*_a_ of **H‐TEDA(BF_4_)_2_** was not previously reported, it was experimentally determined as 2.3 (in water). The p*K*_a_ of analogous doubly‐protonated dicationic DABCO is 2.98 both in water and in DMSO,^[60]^ so we presumed the p*K*_a_ of dicationic **H‐TEDA(BF_4_)_2_** as 2.3 in DMSO. Thus, we examined additives with p*K*_a_ values ranging from 0.3 to 32 (values in DMSO). Protic additives with much higher p*K*_a_ values (range 18.0–31.4)[^61^, ^62^, ^63^] – such as water,^[61]^ methanol,^[61]^ 3‐benzyl‐1‐methyl‐1H‐imidazol‐3‐ium hexafluorophosphate (BnMIM⋅PF_6_)^[62]^ and phenol^[63]^ – inhibited the reaction w.r.t. the baseline result with no additve (Scheme 3).

**Scheme 4:**
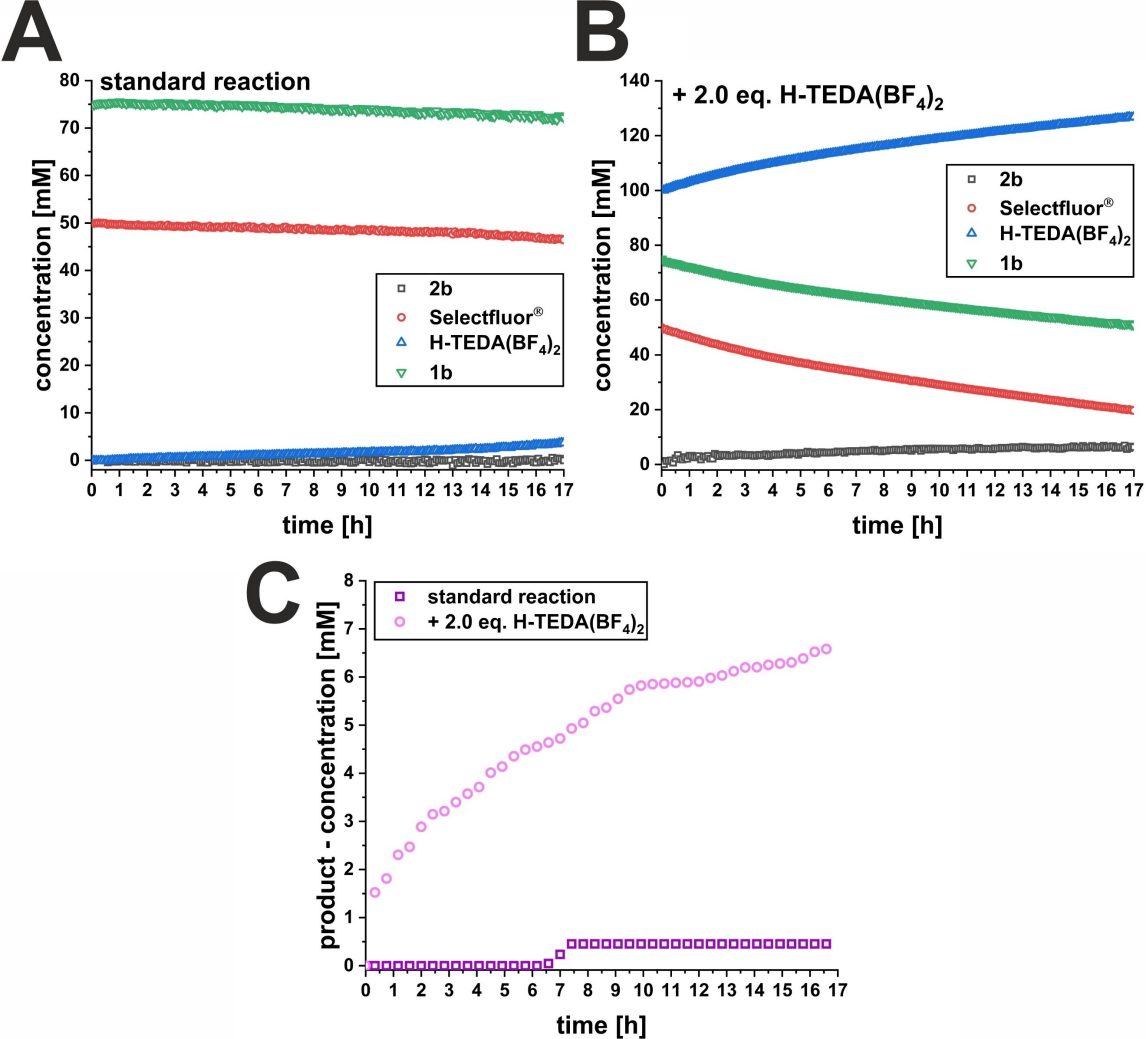
Significant increase in product formation of **2b** in the photochemical C(sp^3^)‐H fluorination reaction with 2.0 eq. **H‐TEDA(BF_4_)_2_** loading. Kinetic profiles detected via ^1^H{^19^F} NMR monitoring (A) under standard conditions and (B) with 2.0 eq. **H‐TEDA(BF_4_)_2_**. C) Comparison of the product formation profiles.

Additives within a p*K*_a_ range 6.4–12.3 (entries 6–8)[^64^, ^65^] – such as acetic acid,^[64]^ triethylammonium tetrafluoroborate (TEA−H⋅BF_4_)^[65]^ and imidazolium tetrafluoroborate (Imid−H⋅BF_4_)^[65]^ – gave comparable, if slightly inferior promotion vs **H‐TEDA(BF_4_)_2_**. In contrast, additives with slightly higher p*K*_a_ values (3.4) – Pyridinium tetrafluoroborate (Py−H⋅BF_4_)^[66]^ and TFA^[24]^ – gave notably lower product yields, 36% and 43% respectively. Acids with lower p*K*_a_ values (range 0.3–1.6) – such as methyl sulfonic acid (MsOH),^[67]^ camphorsulfonic acid (CSA),^[68]^ toluene sulfonic acid (TsOH)^[68]^ and trifluoromethane sulfonic acid (TfOH)^[67]^ – inhibited the reaction completely. That clearly shows that for a successful activation of **SF^®^** a certain p*K*_a_ range between 2–15 (DMSO) is mandatory. However, the reactivity promotion does not trend with increasing acidity indicating other activation factors beyond acidity. The *in situ* NMR irradiation kinetic experiment for **1b** (Scheme 4) revealed almost no product formation after 17 h under standard conditions (Scheme 4, A). At 2.0 eq. of **H‐TEDA(BF_4_)_2_**, product formation even starts directly at the beginning of the *in situ* illumination and steadily increases over time (Scheme 4, B). Overall, after 17 h of *in situ* illumination, only 0.12 mM of product was formed in the standard reaction whereas 6.6 mM of product was generated by addition of 2.0 eq. **H‐TEDA(BF_4_)_2_** (Scheme 4, C). Naturally, the NMR yields in the on‐line NMR experiments are lower due to the lower light intensity (~1/10 the synthetic reaction setup) and the lack of agitation.

Next, we questioned whether the activation is dependent on the ester moiety of the substrate and tested 4‐phenylbutyl benzoate (**1c**), which afforded only 10% of **2c** when treated with 1 mol% photocatalyst (**MFB**) under 400 nm irradiation for 24 h (Table 1, entry 1). With 2.0 eq. of **H‐TEDA(BF_4_)_2_** present at the start of the reaction, the yield of **2c** again increased by almost 7× (68% see Table 1, entry 2). Furthermore, TFA and TEA−H⋅BF_4_ – showed a similar relative activation pattern as for **1b** (Table 1, entries 2,3). The nearly identical reactivity pattern suggests that additives of a certain p*K*_a_ range can also successfully activate **SF^®^**. However, the activation with **H‐TEDA(BF_4_)_2_** is slightly superior, provides a by far better overall mass balance over several cycles and omits the introduction of further molecules that might complicate work‐up/separation.

**Table 1:** Photocatalytic fluorination of **1c** using **MFB** as the photocatalyst with different amounts of additives.

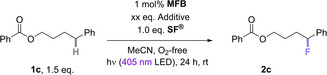		
Entry	Additive/p*K*_a_/eq.	NMR yield (%)
1	–	10
2	**H‐TEDA(BF_4_)_2_**/2.3/2.0	68
3	TEA‐H ⋅ BF_4_/9.0/2.0	63
4	TFA/3.4/2.0	45

**Scheme 5:**
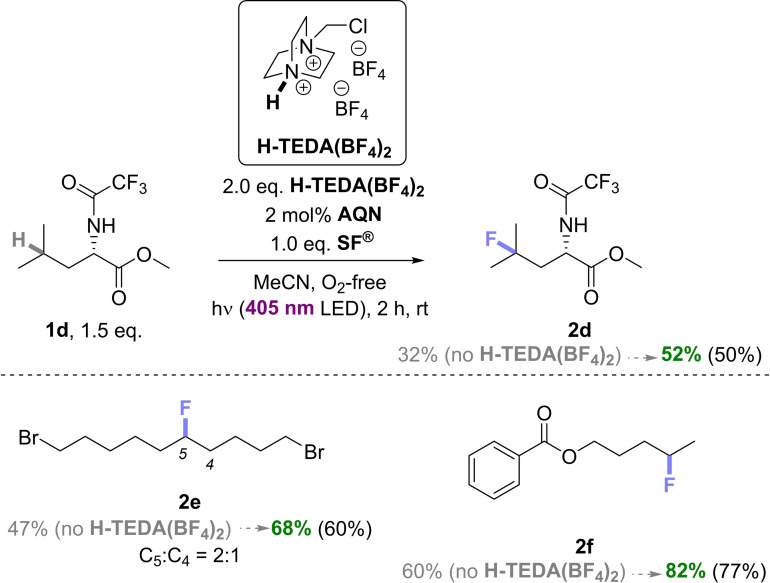
Promoted vs unpromoted photocatalytic fluorinations of **1d**, **1e**, and **1f** with AQN catalyst. NMR yields were determined by ^19^F NMR with pentafluorobenzene as the internal standard (IS). Isolated yields are in parenthesis.

Case Study 2: Next we tested the effect of **H‐TEDA(BF_4_)_2_** on the direct remote fluorination of non‐benzylic C−H bonds by **SF^®^**. We selected the photocatalytic protocol of Tan and co‐workers,^[38]^ using anthraquinone (AQN) as a photocatalyst. In our hands, their standard conditions (1.0 eq. **SF^®^** and 2 mol% AQN), gave **2d** in a yield (32%) comparable to the literature (34%),^[38]^ giving us confidence over our literature reproducibility (Scheme 5). Under the same conditions with 2.0 eq. of **H‐TEDA(BF_4_)_2_** present at the reaction start, **2d**‘s yield increased notably to 55%. We examined 1,10‐dibromodecane (**1e**) and amyl benzoate (**1f**), whose literature yields (41% and 55%, respectively)^[38]^ were successfully reproduced in our hands (47% and 60%, respectively). By adding 2.0 eq. of **H‐TEDA(BF_4_)_2_**, the fluorinated product yields increased by ~20% (68% of **2e** and 82% of **2f**). Thus, promotor **H‐TEDA(BF_4_)_2_** is also effective for fluorinations of non‐benzylic C−H bonds, providing notable yield increases (1.5×).

**Scheme 6:**
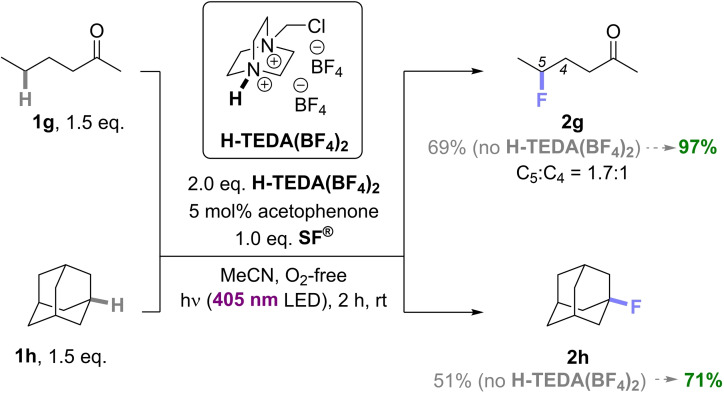
Promoted vs unpromoted photocatalytic fluorinations of **1g** and **1h**. NMR yield is determined by ^19^F NMR with pentafluorobenzene as the IS.

**Scheme 7:**
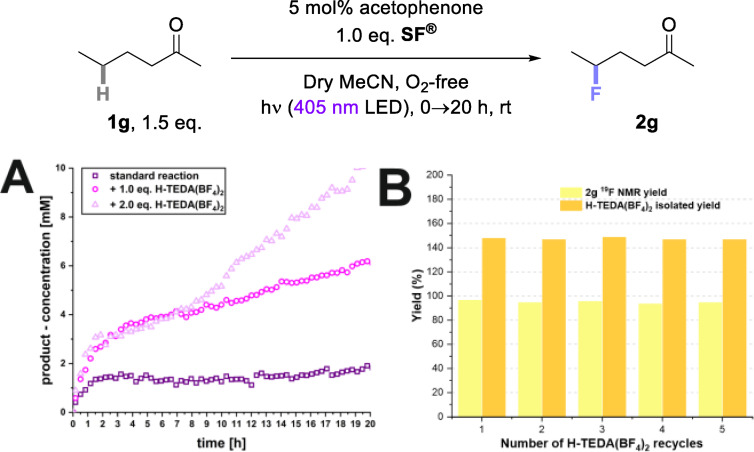
A) The photocatalytic C(sp^3^)‐H fluorination of **1g** shows a significant increase in product formation by a factor of **3** (1.0 eq.) and **5** (2.0 eq.) with exogenous **H‐TEDA(BF_4_)_2_** loading. Kinetic profiles were detected with ^1^H{^19^F} NMR reaction monitoring under standard conditions with 1.0 eq. **H‐TEDA(BF_4_)_2_** and 2.0 eq. **H‐TEDA(BF_4_)_2_** loading. B) Recycling of **H‐TEDA(BF_4_)_2_** in photocatalytic fluorination of **1g** using 2.0 eq. **H‐TEDA(BF_4_)_2_**.

Case Study 3: Next, we clarified whether different photocatalysts could impact the promotion of yield for unactivated substrates. Chen and co‐workers reported acetophenone as a photocatalyst for the direct C−H fluorination of unactivated C(sp^3^)–H bonds under near‐UV light (375–400 nm). Under reported conditions, **2g** was obtained in a good 60% NMR yield,^[37]^ and we wondered if this could be increased further. In our hands with a 405 nm LED, the reported standard conditions (1.0 eq. **SF^®^** and 5 mol% acetophenone), gave **2g** in a yield comparable (69%) to the literature (Scheme 6).^[37]^ Under the same conditions but with 2.0 eq. of **H‐TEDA(BF_4_)_2_**, the yield of **2g** increased notably from 69% to 97%, once again proving the generality of the promotor (Scheme 6). The yield of the fluorinated adamantane (**2h**) increased from 51% to 71% as well. The degree of yield promotion (1.4×) tracked well with Case Study 2 (1.5×), showing how the nature of the photocatalyst is irrelevant to promotion. For an insight into the kinetics of the non‐benzylic C−H fluorination reactions, the model reaction of **1g** (Scheme 6) without, and with different loadings of **H‐TEDA(BF_4_)_2_** was investigated (Scheme 7).

The unpromoted reaction (no added **H‐TEDA(BF_4_)_2_**) showed immediate formation of a small amount of product at an initial rate of 1.39×10^−5^ mM/s. Then, product formation shut down prematurely, speaking to a deactivation of unreacted **SF^®^**. After 2 h, nearly no further product formation occurs. When 1.0 eq. of **H‐TEDA(BF_4_)_2_** was added, the product formation rate was almost tripled to 3.97×10^−5^ mM/s and product formation continued after 20 h. A slightly higher product formation was obtained by addition of 2.0 eq. of **H‐TEDA(BF_4_)_2_** in the first 2 h. After 2 h, product formation continued at a steady, slower rate (12.3×10^−5^ mM/s), clearly leading to a higher overall product yield (Scheme 7, A). That 1.0 eq. and 2.0 eq. of **H‐TEDA(BF_4_)_2_** promotes the later stages of the reaction suggests the generation of more nascent **H‐TEDA(BF_4_)_2_**, which counters the deactivation of unreacted **SF^®^** and increases the overall rate further. Overall, the kinetics of the photocatalytic C(sp^3^)−H fluorination reaction indicated that **H‐TEDA(BF_4_)_2_** loading enhances the reaction rate, and this is inconsistent with a radical chain mechanism (which should not depend on the nature of concentration of chain‐terminated products). Our conclusion is consistent with the fact that previous studies reported low quantum yields <0.15 for such reactions.[^39^, ^45^] Of note from a practicality side, **H‐TEDA(BF_4_)_2_** was recovered quantitatively from **1g**‘s reaction and was reused in iterative reactions up to 5 times (Scheme 7, B), without any erosion of either **2g**’s yield or the (quantitative) recovery of **H‐TEDA(BF_4_)_2_** (see SI for details).

**Scheme 8:**
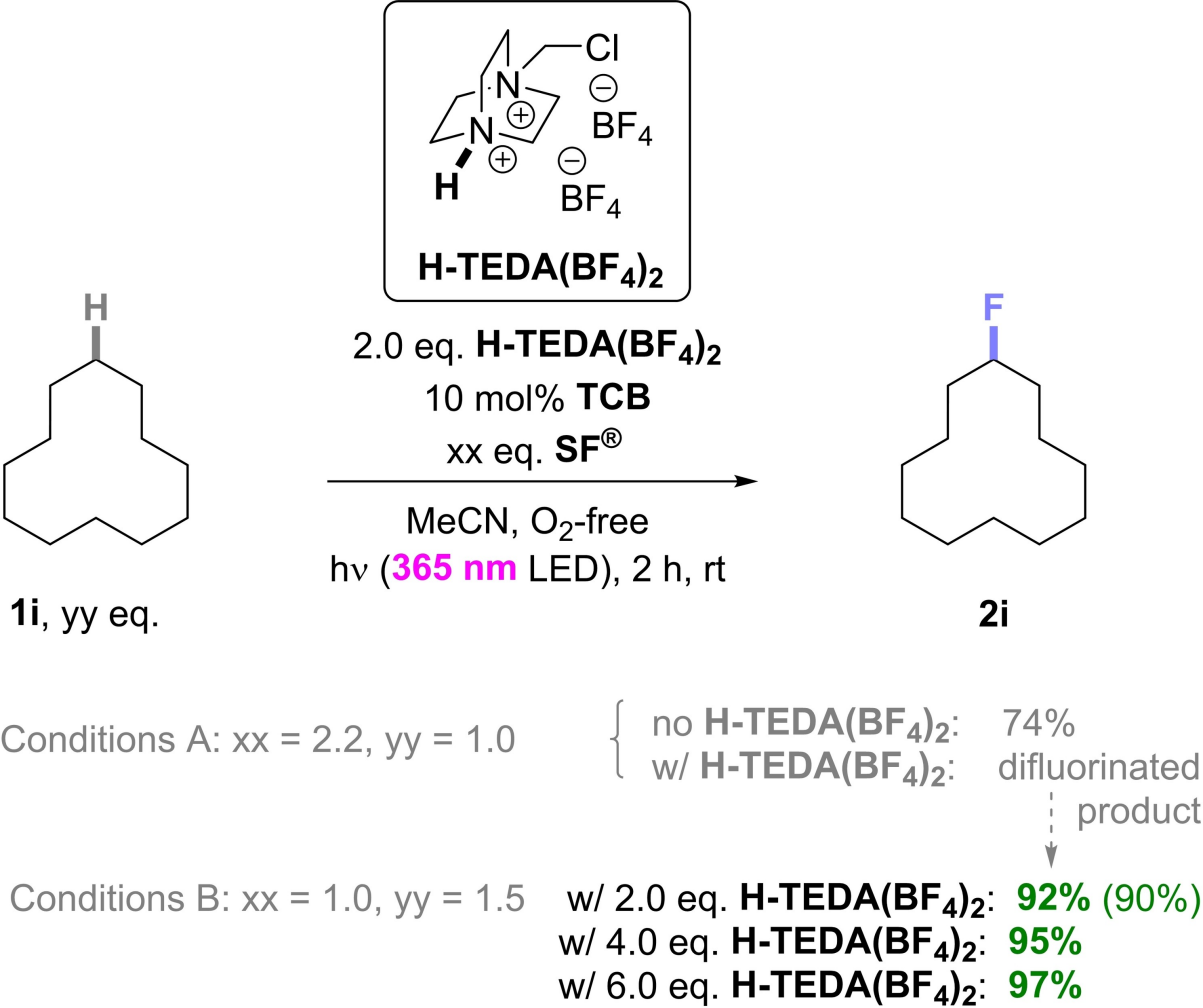
Promoted vs unpromoted photocatalytic fluorination of **1i**. NMR yield is determined by ^19^F NMR with pentafluorobenzene as the IS. Isolated yield is in parenthesis.

Case Study 4: Last, we tested fluorination of hydrocarbons as completely unactivated substrates under conditions reported by Lectka and co‐workers, using 1,2,4,5‐tetracyanobenzene (TCB) as a non‐carbonylic photosensitizer under near UV‐light.^[40]^ Here, neither substrate nor catalyst has a handle (carbonyl group/amine) to interact with **H‐TEDA(BF_4_)_2_**, meaning the only interaction could be between **H‐TEDA(BF_4_)_2_** and **SF^®^**. Lectka and co‐workers’ standard conditions (2.2 eq. **SF^®^** and 10 mol% TCB; 63% of **2i**),^[40]^ in our hands gave **2i** in a slightly higher, but comparable yield of 74% (Scheme 8). However, with addition of 2.0 eq. of **H‐TEDA(BF_4_)_2_**, we observed mainly difluorinated products of **1i**, presumably due to the much higher reactivity of **SF^®^** in the presence of **H‐TEDA(BF_4_)_2_** (Scheme 8). We repeated this experiment with 1.0 eq. **SF^®^** (i. e. as the limiting reagent) and 2.0 eq. **H‐TEDA(BF_4_)_2_** and obtained the product **2i** in 92% yield. Even higher loadings of **H‐TEDA(BF_4_)_2_** (4.0 and 6.0 eq.) led to marginally higher yields of **2i** (95% and 97%, respectively). This data show that not only does **H‐TEDA(BF_4_)_2_** notably improve the yield (up to 97%), but also allows to decrease the amount of **SF^®^** to less than half. Considering the enormous consumption of **SF^®^** in the global production of fluorinated compounds, leading to **H‐TEDA(BF_4_)_2_**/**TEDA(BF_4_)** as waste, the presented change of reaction conditions is highly beneficial for both ecological as well and economic reasons.

#### Reaction Class 2: Photosensitized Auxiliary C(sp^3^)−H Fluorinations

Case Study 5: Next, we pushed the boundaries of **H‐TEDA(BF_4_)_2_** promotion, asking (i) can it compete with our previously reported covalently linked photosensitized auxiliary that also modifies the aggregation state of **SF^®^**?,^[45]^ (ii) does it apply to fluorination reagents other than **SF^®^**? and (iii) how competitive are other additives (Brønsted acids) in these kinds of reactions? We decided to test the effect of different **H‐TEDA(BF_4_)_2_** additive loadings on the reaction of 4‐phenylbutyl 4‐fluorobenzoate **1a** (See the SI file for the details). Under standard unpromoted reaction conditions, a 67% product yield resulted (Table 2, entry 1). With the addition of 2.0 eq. of **H‐TEDA(BF_4_)_2_** to the reaction mixture, an 85% yield of **2a** was obtained after the same period (Table 2, entry 2). However, further increasing the **H‐TEDA(BF_4_)_2_** loading led to lower product yields; e. g., 4.0 eq. and 8.0 eq. of **H‐TEDA(BF_4_)_2_** provided 63% and 17% product yields, respectively (Table 2, entries 3 and 4). Next, we tested if the **H‐TEDA(BF_4_)_2_** additive promotes reactions of other fluorine sources. The standard reaction with Selectfluor II (‘**SF II**’ i. e., **SF^®^** where the Cl atom is replaced by H) instead of **SF^®^** provided only 36% of **2a** (Table 2, entry 5). However, the same reaction with addition of 2.0 eq. **H‐TEDA(BF_4_)_2_** gave 64% of **2a** (Table 2, entry 6). The standard reaction with *N*‐fluorobenzenesulfonimide (**NFSI**) instead of **SF^®^** did not afford **2a** (Table 2, entry 7). However, the same reaction with addition of 2.0 eq. **H‐TEDA(BF_4_)_2_** provided 27% of **2a** (Table 2, entry 8). Thus, the promotionary effect of **H‐TEDA(BF_4_)_2_** is not limited to **SF^®^** and is general to other fluorine sources in radical fluorinations.

**Table 2:** Photofluorination of **1a** in presence of different fluorinating agents, additives and different loadings.

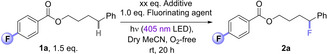			
Entry	Reagents	Additive/p*K*_a_/eq.	NMR yield (%)
1	**SF^®^**	–	67
2	**SF^®^**	**H‐TEDA(BF_4_)_2_**/2.3/2.0	85
3	**SF^®^**	**H‐TEDA(BF_4_)_2_**/2.3/4.0	63
4	**SF^®^**	**H‐TEDA(BF_4_)_2_**/2.3/8.0	17
5	**SF II**	–	36
6	**SF II**	**H‐TEDA(BF_4_)_2_**/2.3/2.0	64
7	**NFSI**	–	0
8	**NFSI**	**H‐TEDA(BF_4_)_2_**/2.3/2.0	27
9	**SF^®^**	Acetic acid/12.3/2.0	59
10	**SF^®^**	TEA‐H ⋅ BF_4_/9.0/2.0	65
11	**SF^®^**	Imid−H ⋅ BF_4_/6.4/2.0	65
12	**SF^®^**	Py‐H ⋅ BF_4_/3.4/2.0	62
13	**SF^®^**	TFA/3.4/2.0	90

**Scheme 9:**
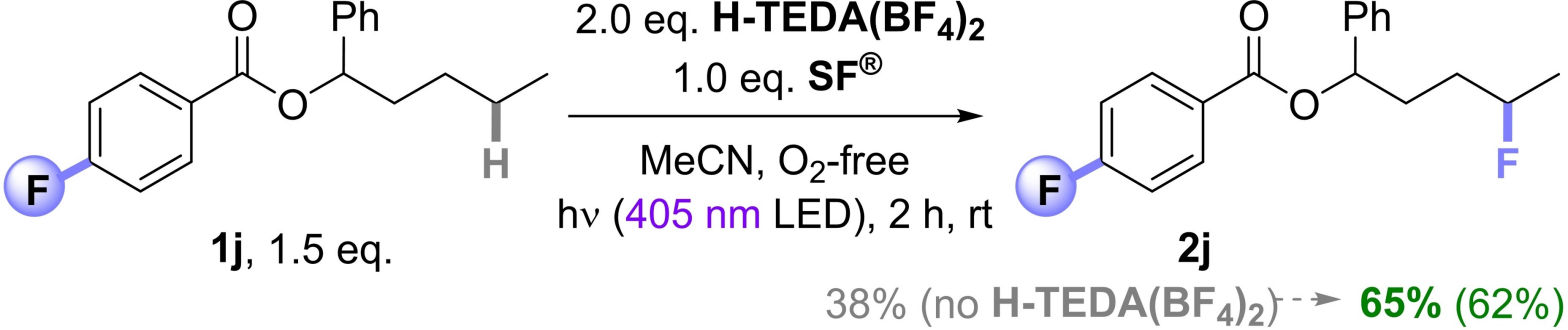
Promoted vs unpromoted photosensitized auxiliary fluorination of **1 j**. NMR yields were determined by ^19^F NMR with pentafluorobenzene as the IS. Isolated yield is in parenthesis.

Regarding the effect of other Brønsted acidic additives, most of those with higher p*K*_a_ values – such as acetic acid (p*K*_a_ 12.3),^[64]^ triethylammonium tetrafluoroborate (TEA−H⋅BF_4_) (p*K*_a_ 9.0),^[65]^ imidazolium tetrafluoroborate (Imid−H⋅BF_4_) (p*K*_a_ 6.4),^[65]^ pyridinium tetrafluoroborate (Py−H⋅BF_4_) (p*K*_a_=3.4)^[66]^ – gave similar results to the unpromoted reaction (Table 2, entries 9–12). In contrast, TFA (p*K*_a_=3.4, identical to Py−H⋅BF_4_)^[64]^ gave a yield even slightly higher than **H‐TEDA(BF_4_)_2_** – 90 % (Table 2, entry 13). This is reminiscent of Case Study 1, where TFA led to a comparable, if slightly lower yield than **H‐TEDA(BF_4_)_2_** for the benzylic fluorination. Again, the data show that p*K*_a_ cannot be the only criterion, making predictions difficult. Furthermore, a comparison with Scheme 3 shows that while other additives are sensitive to the individual reaction system, the effective activation with **H‐TEDA(BF_4_)_2_** is stable throughout the systems. One further auxiliary‐loaded substrate with an additional benzylic position was texted (Scheme 9), which again led to an increase in yield from 38% to 65% upon promotion.

#### Reaction Class 3: Thermal C(sp^3^)−H Radical Fluorinations

Case Study 6: Having demonstrated the generality of **H‐TEDA(BF_4_)_2_** as a promoter for photochemical fluorinations, we sought to assess its promotionary impact on thermal fluorination reactions. Baxter and co‐workers reported a radical C(sp^3^)−H fluorination method^[46]^ using **SF^®^**, a catalytic amount of silver nitrate and an unprotected amino acid – glycine – as a radical precursor. One substrate for which the method was inefficient was 4‐methyl acetophenone (**1 k**). The standard conditions – in our hands – provided only 8% of fluorinated product **2k** (Table 3, entry 1), and a catalytic quantity of **H‐TEDA(BF_4_)_2_** (0.1 eq.) made no difference (Table 3, entry 2). By adding 2.0 eq. of **H‐TEDA(BF_4_)_2_** to the reaction mixture (entry 3), the yield of **2k** more than doubled (20%). Interestingly, presence of 2.0 eq. **H‐TEDA(BF_4_)_2_** *in the absence of glycine* led to a 37% yield of **2k** (Table 3, entry 3) which ~doubled to 66% with a longer reaction time of 48 h (Table 3, entry 4). Increasing the loading of **H‐TEDA(BF_4_)_2_** further to 6.0 eq. or 10.0 eq. increased the yield further up to 77%, giving a clear trend both in the presence and absence of glycine (Table 3, entries 5–9). When other protic additives with similar p*K*_a_ values were tested, Py−H⋅BF_4_ and TFA halted reactivity in the presence of glycine. Presumably, glycine is deactivated by protonation, while the proton of **H‐TEDA(BF_4_)_2_** may be occupied in aggregates, see studies *vide infra*. Only Py−H⋅BF_4_ increased the yield in the absence of glycine (Table 3, entries 10–11). This corroborates the sensitivity of other Brønsted acid‐type activators to the individual experimental conditions, as already demonstrated in the previous examples and confirms **H‐TEDA(BF_4_)_2_** as the most robust promotor.

**Table 3:** Thermal Ag‐catalyzed Fluorination of **1k** with Different Additives and Additive Loadings.

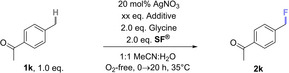		
Entry	Additive/p*K*_a_/eq.	NMR yield (%)
1	–^*a*^	8
2	**H‐TEDA(BF_4_)_2_**/2.3/0.1	3
3	**H‐TEDA(BF_4_)_2_**/2.3/2.0	20
4	**H‐TEDA(BF_4_)_2_** ^*b*^/2.3/2.0	37
5	**H‐TEDA(BF_4_)_2_** ^*a,b*^/2.3/2.0	66
6	**H‐TEDA(BF_4_)_2_**/2.3/6.0	41
7	**H‐TEDA(BF_4_)_2_** ^*a,b*^/2.3/6.0	68
8	**H‐TEDA(BF_4_)_2_**/2.3/10.0	58
9	**H‐TEDA(BF_4_)_2_** ^*a,b*^/2.3/10.0	77
10	Py‐H ⋅ BF_4_/3.4/2.0	Traces
11	Py‐H ⋅ BF_4_^*b*^/3.4/2.0	18
12	TFA/3.4/2.0	0
13	TFA/3.4/2.0	0

^*a*^ Reaction time 48 h. ^*b*^ Without glycine.

**Scheme 10:**
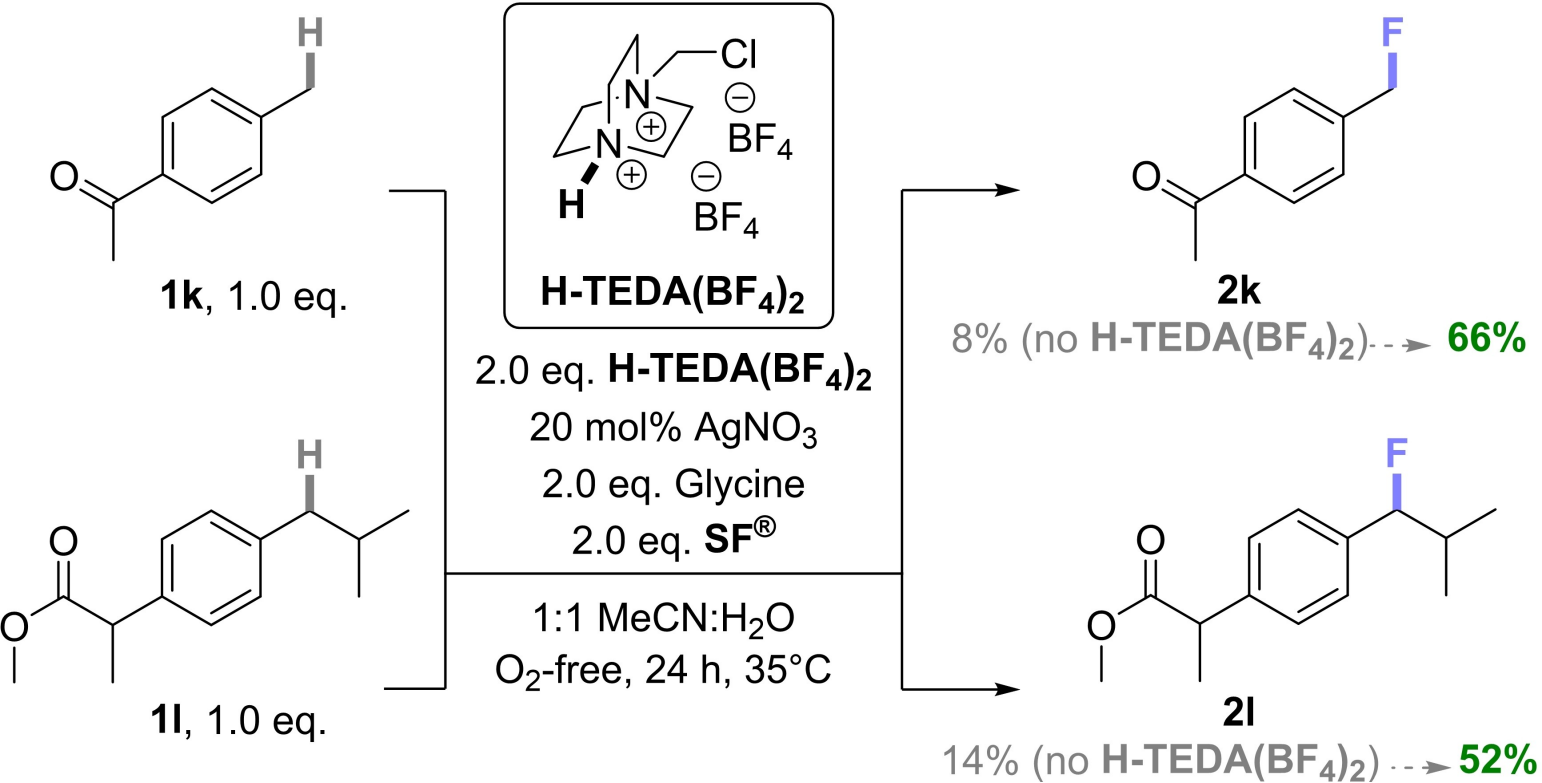
Promoted vs unpromoted thermal Ag‐catalyzed fluorinations of **1k** and **1l**. NMR yields determined by ^19^F NMR with pentafluorobenzene as the IS.

**Scheme 11:**
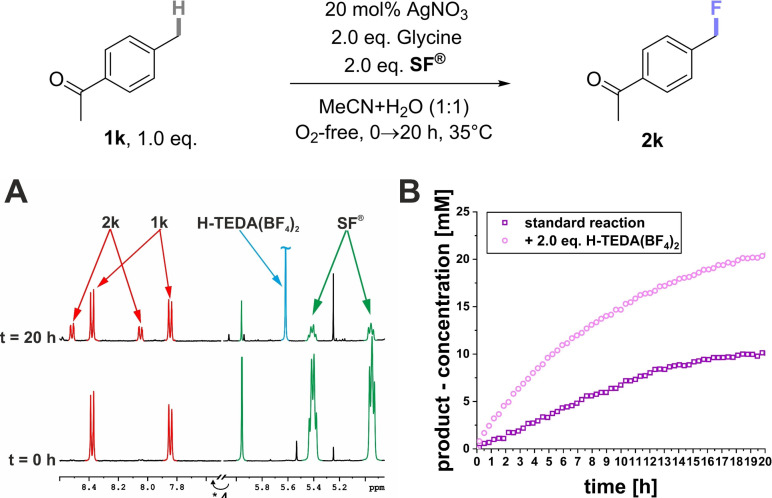
**Thermal C(sp^3^)‐H fluorination**: The addition of 2.0 eq. exogenous **H‐TEDA(BF_4_)_2_** results in a twofold increase in product formation of **2k**. ^1^H NMR reaction monitoring of **H‐TEDA(BF_4_)_2_** ‘s promotionary effect before and after illumination. B) Comparison of the product formation profiles of **2k** under standard conditions and with 2.0 eq. **H‐TEDA(BF_4_)_2_** loading.

Elsewhere, Baxter and co‐workers fluorinated the more electron‐rich benzylic position of ibuprofen methyl ester (**1l**),^[46]^ using 5.0 eq. of both glycine and **SF^®^** for this particular substrate (46% literature yield of **2l**).^[46]^ In our hands, when using 2.0 eq. of both glycine and **SF^®^**, only 14% of **2l** was obtained (Scheme 10). By adding 2.0 eq. of **H‐TEDA(BF_4_)_2_** to the reaction, the yield of **2l** increased to 52%. In summary, addition of the **H‐TEDA(BF_4_)_2_** promoter provided an even higher yield than the literature and allowed us to employ far less (2.5×) equivalents of **SF^®^** and to omit glycine. Since **SF^®^** is substantially more expensive to prepare than **H‐TEDA(BF_4_)_2_**, the cost and sustainability benefits of our discovery are clear. To explore further the promotionary effect of **H‐TEDA(BF_4_)_2_** on thermal radical fluorinations, the reaction kinetics of **1k**’s reaction without and with **H‐TEDA(BF_4_)_2_** were followed by *in situ* NMR monitoring within a variable temperature NMR probe (Scheme 11). The unpromoted reaction generated approximately 10 mM of product after 20 h of irradiation, while the reaction promoted by 2.0 eq. of **H‐TEDA(BF_4_)_2_** gave approximately 20 mM after the same time period (Scheme 11, B). Calculated initial rates revealed that 2.0 eq. of **H‐TEDA(BF_4_)_2_** loading increased the product formation rate by a factor of 3 (see SI section 3.4.2). Not only was the initial rate of the reaction faster in the presence of **H‐TEDA(BF_4_)_2_**, the final yield upon which the reaction converged was doubled.

Providing a notable benefit to the efficiencies and kinetics of all six case studies aforementioned, including photochemical, photocatalytic and thermal phenotypes, the generality of **H‐TEDA(BF_4_)_2_** as a promotor of radical fluorination reactions was clear. We then turned to more detailed mechanistic studies to identify the nature of this promotionary effect.

#### Aggregation Studies

**Scheme 12:**
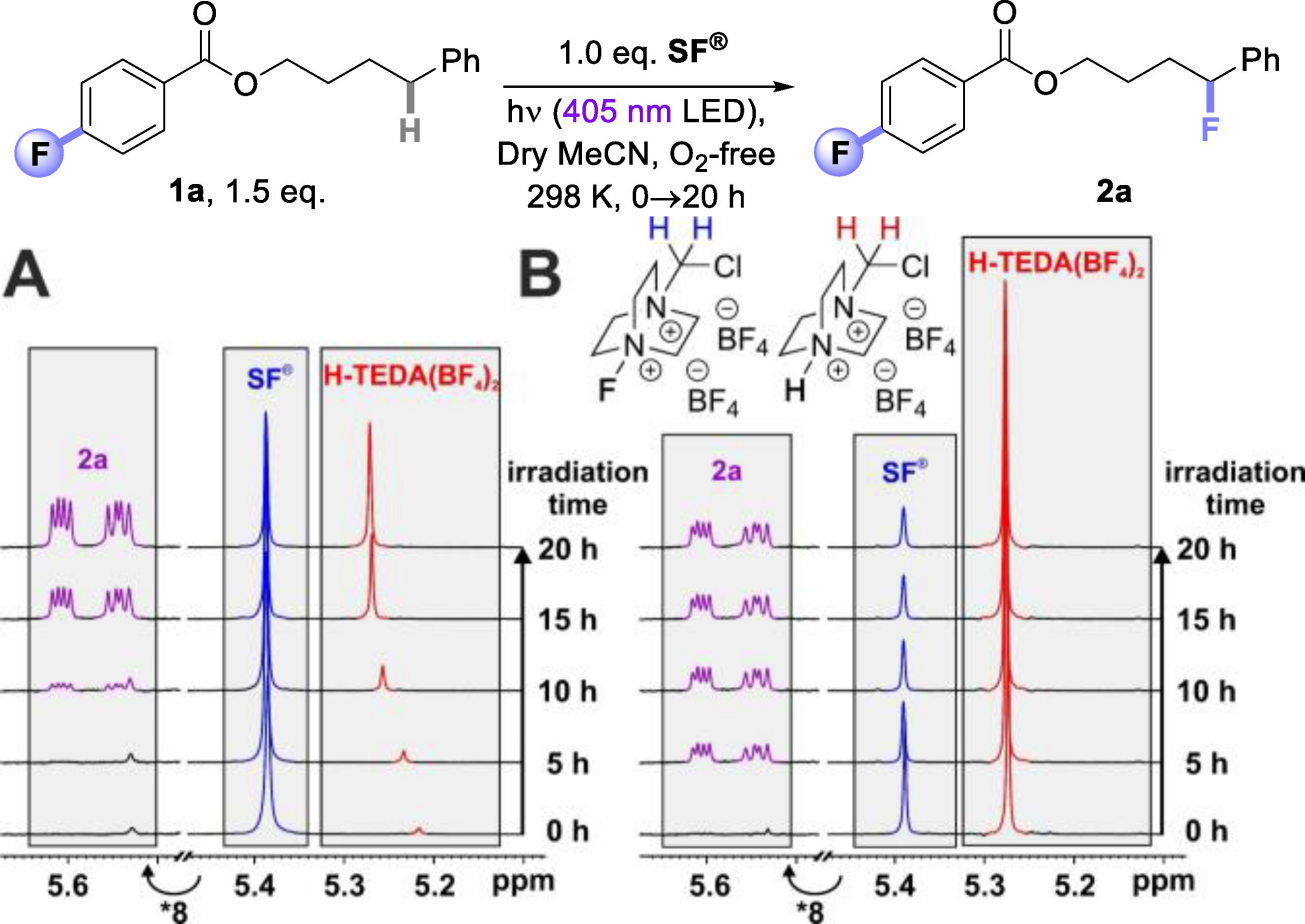
Slow heteroaggregation formation vs. pre‐heteroaggregation: A) Under standard conditions in the photosensitized auxiliary C(sp^3^)−H fluorination reaction of **1a, H‐TEDA(BF_4_)_2_** is formed and heteroaggregates with **SF** ^®^ during illumination, indicated by its chemical shift change upon increasing concentrations in the ^1^H spectra. B) With 1.0 eq. **H‐TEDA(BF_4_)_2_** loading the final heteroaggregate exists already at the beginning.

We hypothesize that intermolecular interactions between **SF^®^** and **H‐TEDA(BF_4_)_2_** potentially *via* higher aggregation leads to activation of **SF^®^** in the reaction mixture. In case **H‐TEDA(BF_4_)_2_** directly activates **SF^®^** as a promoter, an intermolecular interaction between these molecules should be detected. Therefore, substrate **1a** was fluorinated by **SF^®^** (Case Study 5) in the absence of **H‐TEDA(BF_4_)_2_** and ^1^H NMR spectra were recorded periodically after 5 h of *in situ* irradiation. Indeed, as the concentration of nascent **H‐TEDA(BF_4_)_2_** increased, an evident downfield shift of its N−C*H*_2_−Cl NMR peak was observed during *in situ* irradiation (Scheme 12, A). The maximal chemical shift ceased at a certain point (~5.28 ppm), and significant product formation could be detected by increased **H‐TEDA(BF_4_)_2_** concentration. When performing the same experiment with addition of 1.0 eq. exogeneous **H‐TEDA(BF_4_)_2_** (alone, its N−CH_2_−Cl NMR peak comes at 5.20 ppm in MeCN‐d_3_) the chemical shift observed was *already* ~5.28 ppm from the beginning, and notable formation of the product started instantly (Scheme 12, B). This chemical shift of **H‐TEDA(BF_4_)_2_** remained consistent across all case studies, irrespective of the reaction conditions and thus serves as a first indication of **H‐TEDA(BF_4_)_2_** heteroaggregation with **SF^®^** with increasing concentration.

To manifest aggregation trends of **SF^®^** and **H‐TEDA(BF_4_)_2_**, we performed diffusion ordered spectroscopy (DOSY) measurements for the pure components and for the reaction mixture and calculated the related volumes to determine aggregation trends (Table 4). At synthetic reaction concentrations (Table 4, entry 1), precipitation of **H‐TEDA(BF_4_)_2_** occurs due to limited solubility in CD_3_CN. Therefore, we used maximum concentrations of 90 mM for **SF^®^** and **H‐TEDA(BF_4_)_2_** for reliable aggregation studies. The data in Table 4 (entries 2–5) show that the volume of **H‐TEDA(BF_4_)_2_** nearly doubles from 1 mM to 90 mM (506 Å^3^ and 934.9 Å^3^, respectively) and is then close to 974 Å^3^ at synthetic reaction concentrations (Table 4, entries 1 and 2). The monomeric volume of **H‐TEDA(BF_4_)_2_** was calculated to be 321 Å^3^ (see SI section 4.1.4). This indicates an average aggregation number of 3 for **H‐TEDA(BF_4_)_2_** under synthetic conditions in MeCN as solvent. **SF^®^** is significantly lower aggregated than **H‐TEDA(BF_4_)_2_** at 90 mM (Table 4, entries 2 and 6). This offset in aggregation might be explained by pure ion pair aggregation of **SF^®^**, while **H‐TEDA(BF_4_)_2_** can undergo ion pairing and hydrogen bonding. Even more interesting, a further increase in volume for both **SF^®^** and **H‐TEDA(BF_4_)_2_** was observed in the 1 : 1 component mixture at 90 mM (Table 4, entry 7). The volume of **SF^®^** even increased by 47% (entries 6 and 7) while for **H‐TEDA(BF_4_)_2_** a more moderate increase of 19% was observed (entries 2 and 7). These results clearly indicate a preferred heteroaggregation between **SF^®^** and **H‐TEDA(BF_4_)_2_** over the homoaggregation of **SF^®^** or **H‐TEDA(BF_4_)_2_**. Furthermore, similar ratios of diffusion coefficients for the ^−^BF_4_ anion for both the homo‐ and heteroaggregates indicate that ion pair formation is not the exclusive determining factor for aggregation (entries 12–14). Despite the higher overall ion concentration in the homoaggregate situation at 90 mM (entries 2 and 6), the volumes of **SF^®^** and **H‐TEDA(BF_4_)_2_** remain constant at a lower ion concentration of 40 mM in their 1 : 1 component mixture (entry 8). Again, this corroborates heteroaggregate formation of **SF^®^** and **H‐TEDA(BF_4_)_2_** over homoaggregate formation. Therefore, we suggest additional intermolecular ^+^N−H—F−N^+^ and possible ^+^N−H—Cl−N^+^ interactions within the aggregate as a driving force for the preferred heteroaggregate formation (see Scheme 14A, *vide infra*). This complex formation is well documented for **SF^®^** and Lewis bases in the literature.[^69^, ^70^, ^71^] It is known that organofluoride F atoms form strong H bonds with N−H donors.[^72^, ^73^] Subsequently, we determined the self‐diffusion coefficients and volumes for **SF^®^** and **H‐TEDA(BF_4_)_2_** with different **H‐TEDA(BF_4_)_2_** loadings. As predicted, enhancing the concentration of **H‐TEDA(BF_4_)_2_** in the **SF^®^**/**H‐TEDA(BF_4_)_2_** mixture markedly increases the volumes of both components (Table 4, entries 8–11). Thus, **H‐TEDA(BF_4_)_2_** loading appears to promote aggregation beyond heterodimer formation.

**Table 4:** Volumes of **SF^®^** and **H‐TEDA(BF_4_)_2_**, pure and with various **H‐TEDA(BF_4_)_2_** loadings in CD_3_CN at 35 °C, measured by DOSY NMR experiments (for details, see SI section 4.1.4). The highest volumes are reached with 1 : 1 heteroaggregates.

Entry	Compounds	C [mM]	Average volume [Å^3^]^a^
1	**H‐TEDA(BF_4_)_2_** (precipitation)	209	974
2	**H‐TEDA(BF_4_)_2_**	90	935
3	**H‐TEDA(BF_4_)_2_**	50	878
4	**H‐TEDA(BF_4_)_2_**	20	797
5	**H‐TEDA(BF_4_)_2_**	1.0	506
6	**SF^®^**	90	718
7	**SF^®^/H‐TEDA(BF_4_)_2_**	90/90	**SF^®^**: 1055 **H‐TEDA(BF_4_)_2_**: 1111
8	**SF^®^/H‐TEDA(BF_4_)_2_**	40/40	**SF^®^**: 1045 **H‐TEDA(BF_4_)_2_**: 1040
9	**SF^®^/H‐TEDA(BF_4_)_2_**	40/30	**SF^®^**: 813 **H‐TEDA(BF_4_)_2_**: 792
10	**SF^®^/H‐TEDA(BF_4_)_2_**	40/20	**SF^®^**: 763 **H‐TEDA(BF_4_)_2_**: 710
11	**SF^®^/H‐TEDA(BF_4_)_2_**	40/10	**SF^®^**: 766 **H‐TEDA(BF_4_)_2_**: 696
12	**BF_4_** of **SF^®^**	90	461
13	**BF_4_** of **H‐TEDA(BF_4_)_2_**	90	504
14	**BF_4_** of **SF^®^/H‐TEDA(BF_4_)_2_**	90	620

^a^ The absolute volumes may vary due to the presence of ionic interactions, but the relative volumes clearly indicate the qualitative trend of aggregation.

**Scheme 13:**
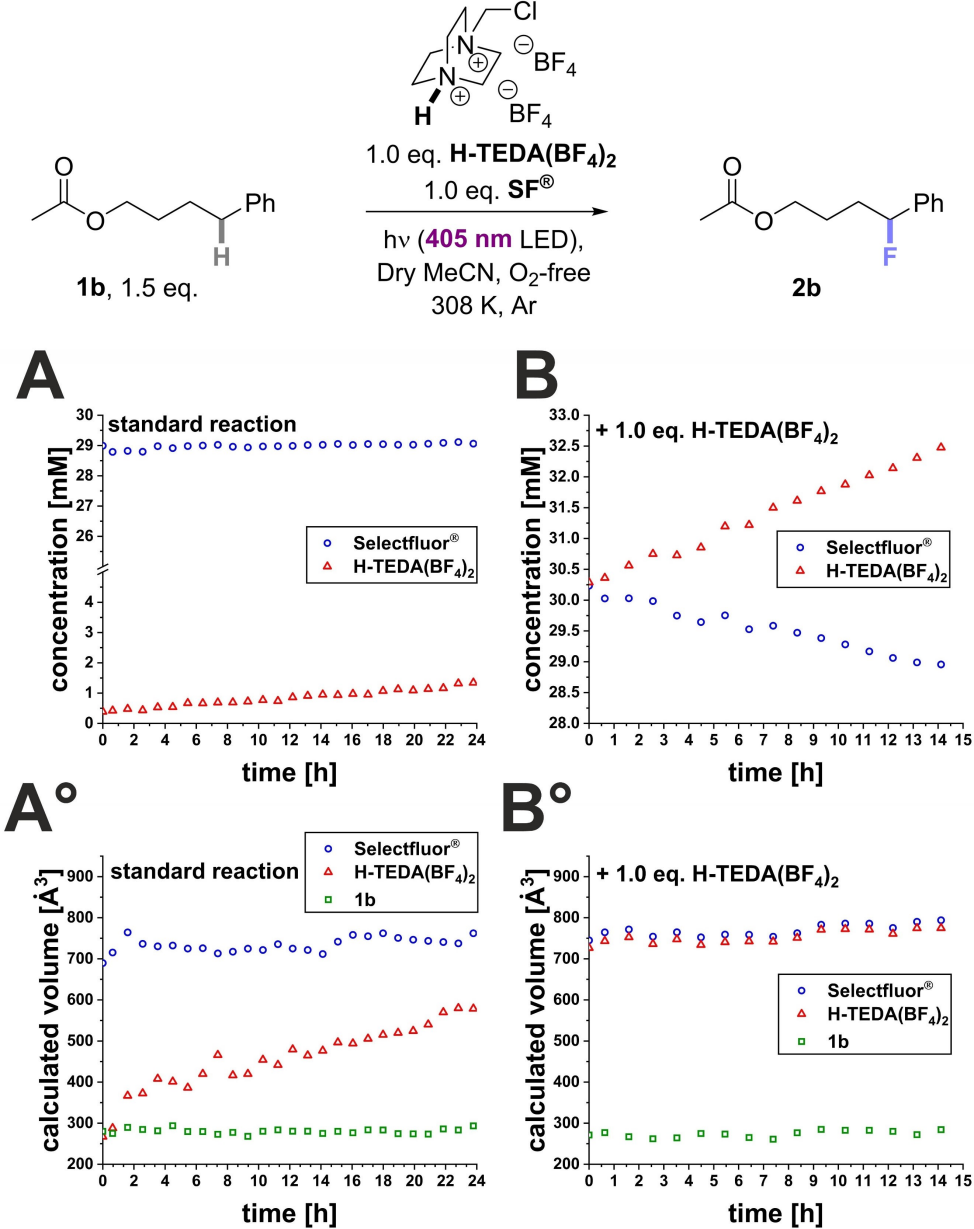
Under standard conditions the nascent **H‐TEDA(BF_4_)_2_** forms only small, less reactive aggregates for hours (A°), while the larger, more reactive aggregates are present from the start with 1.0 eq. **H‐TEDA(BF_4_)_2_** (B°). Change in concentration of **SF^®^** and **H‐TEDA(BF_4_)_2_** during the photochemical C(sp^3^)−H fluorination of **1b** under standard conditions (A) and with 1.0 eq. **H‐TEDA(BF_4_)_2_** loading (B). Simultaneous ^1^H‐NMR aggregation monitoring by DOSY experiments (A°, B°).

To manifest the effects of **H‐TEDA(BF_4_)_2_** loading during the reaction, DOSY experiments and simultaneous ^1^H NMR kinetic measurements were performed in combination with *in situ* illumination (see SI section 4.1.5). As model system we selected photochemical reaction conditions, since during the induction period the highly reactive aggregate should be absent or extremely low in concentration. As evident from the consumption of **SF^®^**, the reaction starts directly for the **H‐TEDA(BF_4_)_2_** promoted experiment, while under the standard conditions no conversion can be detected (see Scheme 13, A/B). For the **H‐TEDA(BF_4_)_2_** promoted experiment, the volumes of **SF^®^** and **H‐TEDA(BF_4_)_2_** hardly change during *in situ* illumination (Scheme 13, B°). Thus, an aggregation state of **SF^®^** and **H‐TEDA(BF_4_)_2_** of approx. 700 Å^3^ at concentrations of 30 mM each allows high reactivity. In contrast, for the unpromoted reaction, the aggregation state of **H‐TEDA(BF_4_)_2_** increases during the reaction (Scheme 13, A°).

The DOSY data for both **SF^®^** and **H‐TEDA(BF_4_)_2_** clearly show that the nascent **H‐TEDA(BF_4_)_2_** is initially not included in the **H‐TEDA(BF_4_)_2_**/**SF^®^** heteroaggregate. Of course, its gradual inclusion in the reactive **H‐TEDA(BF_4_)_2_**/**SF^®^** aggregate can explain the induction period as observed in the previous reaction studies (Scheme 2). This nicely explains the experimental observation that nascent **H‐TEDA(BF_4_)_2_** formed during the reaction *does not substitute the more reactive **H‐TEDA(BF***_***4***_ ***)***_***2***_/***SF*** ^***®***^ *aggregate formed by adding **H‐TEDA(BF***_***4***_ ***)***_***2***_ *at the start of the reaction*. Taken together, aggregation and concentration monitoring during the reaction indicated that the formation of the reactive heteroaggregate requires certain **H‐TEDA(BF_4_)_2_** concentrations in solution. Consequently, **H‐TEDA(BF_4_)_2_** loading prior to the reaction allows immediate aggregation of the components and thus activates **SF^®^** at the beginning of the reaction, eliminating induction phases in which the concentration of nascent **H‐TEDA(BF_4_)_2_** is required to increase.

**Scheme 14:**
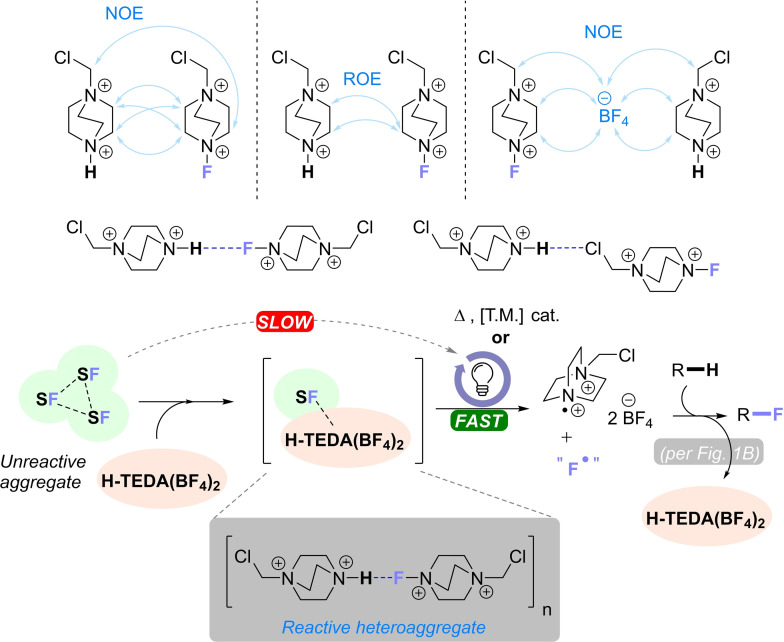
(A) Possible interaction modes of the **SF^®^**/**H‐TEDA(BF_4_)_2_** aggregates based on 2D NMR experiments. (B) Proposed general reaction mechanism of the radical fluorination reactions promoted by **H‐TEDA(BF_4_)_2_**.

Next, more information about the structure and interactions within the **H‐TEDA(BF_4_)_2_**/**SF^®^** aggregate was gathered. As per the aforementioned model system, a 1 : 1 mixture with concentrations of 90 mM of both components was studied in CD_3_CN. The acidic ^+^N−H proton signal of pure **H‐TEDA(BF_4_)_2_** – a broad singlet at 7.11 ppm – shifts to 7.45 ppm in this 1 : 1 mixture, while no other signals of **H‐TEDA(BF_4_)_2_** show any change (see SI section 5.1). This ^+^N−H shift is typical for the formation of a hydrogen bond involving the acidic proton of **H‐TEDA(BF_4_)_2_** and correlates directly with the amount of **SF^®^**. In contrast, ion pair aggregation can occur without chemical shift changes as observed in previous investigations.^[74]^ To verify whether there are specific intermolecular interactions between the components, we performed ^1^H ^1^H NOESY, ^1^H ^1^H ROESY and ^1^H ^19^F HOESY experiments at lower temperatures (230 K). These low temperatures are applied to reduce exchange processes and promote preferred conformations. Cross peaks of the remaining signals in the ^1^H ^1^H NOESY and ^1^H ^1^H ROESY experiments showed multiple intermolecular NOE contacts between the cations of **SF^®^** and **H‐TEDA(BF_4_)_2_** (see Scheme 14A and SI chapter 5.1). The ^1^H ^19^F HOESY experiment could not reveal any N^+^‐H⋅⋅⋅F‐^+^N interactions due to remaining exchange processes. However, the ^1^H ^1^H NOESY and ^1^H ^1^H ROESY at 230 K reveal contacts of **H‐TEDA(BF_4_)_2_** to both sides of the **SF^®^** structure, confirming possible hydrogen bond formation where Cl and F atoms potentially serve as H‐bond acceptors in the **H‐TEDA(BF_4_)_2_**/**SF^®^** aggregate (see Scheme 14A and SI section 5.1).

The F atom is known to be a stronger acceptor than the Cl atom in the literature,[^72^, ^73^] however, it may be weaker in this case due to the presence of the positively charged N atom that bears it. Overall, the general downstream mechanism of the **H‐TEDA(BF_4_)_2_** – induced fluorination reactions may resemble that proposed in the literature,[^36^, ^37^, ^38^, ^39^, ^40^, ^41^, ^42^, ^43^, ^44^, ^45^] however we propose initial steps of the aggregation that are essential for the activation of **SF^®^** (Scheme 14, B).

## Conclusions

We report the discovery of **H‐TEDA(BF_4_)_2_** as a highly efficient, cheap, performance‐enhancing additive repurposed from chemical waste that increases the rates and final reaction yields for various direct C(sp^3^)−H fluorination reactions, including those driven both photochemically and thermally. Reaction yields were increased as much as triple, and the duration of reactions could be shortened as dramatically as from 48 h to 2 h. This study also highlights an often overlooked but increasingly important mechanistic aspect of reactant aggregation in radical reactions. In this case Selectfluor^®^‘s aggregation state profoundly influences various radical fluorination reactions, and may well be – beyond temperature, catalyst, or light intensity – the *key* reactivity‐determining influence. Thorough DOSY investigations of Selectfluor^®^ and **H‐TEDA(BF_4_)_2_** we confirmed enhanced aggregation of both components by increased **H‐TEDA(BF_4_)_2_** concentrations. Rather than Selectfluor^®^ itself, NMR experiments confirmed that a heteroaggregate of Selectfluor^®^ and **H‐TEDA(BF_4_)_2_** is the active species in radical C−H fluorination reactions. Finally, showing the generality of the phenomenon, other individually fine‐tuned Brønsted ′acidic‐type′ additives can also serve as promoters (albeit not by acidity), although only in a ‘single‐use’ fashion. **H‐TEDA(BF_4_)_2_** is the most robust across all case studies herein, and is fully recycleable in quantitative yield by simple precipitation/filtration (see SI). Overall, we believe that this discovery of aggregative ion pair activation of Selectfluor^®^ could lead to more ecological and economical application of Selectfluor^®^ in the future.

## Conflict of Interests

A provisional patent has been filed by the authors based in part on this work: EP 23 205 559 0.

1

## Supporting information

As a service to our authors and readers, this journal provides supporting information supplied by the authors. Such materials are peer reviewed and may be re‐organized for online delivery, but are not copy‐edited or typeset. Technical support issues arising from supporting information (other than missing files) should be addressed to the authors.

Supporting Information

## Data Availability

The data that support the findings of this study are available in the supplementary material of this article.
