# Supplementary material for: Protodefluorinated Selectfluor® Aggregatively Activates Selectfluor® for Efficient Radical C(sp3)−H Fluorination Reactions
Source: ChemSusChem. 2024 Oct 30;17(23):e202401057. doi: 10.1002/cssc.202401057 (PMC11632574; doi:10.1002/cssc.202401057)
Supplement: Supplementary file 1 — Supporting Information [file CSSC-17-e202401057-s001.pdf]

ChemSusChem

Supporting Information

Protodefluorinated Selectfluor® Aggregatively Activates Selectfluor® for Efficient Radical C(sp^3)−H Fluorination Reactions

Shahboz Yakubov, Bastian Dauth, Willibald J. Stockerl, Wagner da Silva, Ruth M. Gschwind,* and Joshua P. Barham*

Supporting Information File

Protodefluorinated Selectfluor® Aggregatively Activates Selectfluor® for Efficient Radical C(sp^3)−H Fluorinations

Shahboz Yakubov,‡ Bastian Dauth,‡ Willibald J. Stockerl, Wagner M. da Silva, Ruth M. Gschwind* and Joshua P. Barham*

Universität Regensburg, Fakultät für Chemie und Pharmazie, 93040 Regensburg, Germany

‡ These authors contributed equally. * Corresponding Authors.

E-mails: Ruth.Gschwind@chemie.uni-regensburg.de ; Joshua-Philip.Barham@chemie.uni-regensburg.de

Table of Contents

1 Experimental 2

1.1 General Comments 2

1.2 Fluorination of substrates with different amounts of additives 4

1.3 Triplicate results 9

1.4 Calculating pK_a of **H-TEDA(BF$_4$)$_2$** in H_2O 12

1.5 Isolation of **H-TEDA(BF$_4$)$_2$** After Fluorination Reactions 13

1.6 Recycling of **H-TEDA(BF$_4$)$_2$** After Fluorination Reactions 13

1.7 Synthesis of **H-TEDA(BF$_4$)$_2$** from DABCO 15

2 Synthesis 15

2.1 Synthesis of Starting Materials 15

2.2 Synthesis of Additives 18

2.3 Synthesis of Fluorinated products 19

3 Advanced NMR-Spectroscopic Investigations 26

3.1 General 26

3.2 Chemicals 26

3.3 General method for quantitative *in situ* illumination NMR reaction monitoring 26

3.4 Reaction monitoring of fluorination reactions with Selectfluor® and protonated Selectfluor® 26

3.4.1 NMR sample preparation 26

3.4.2 Reaction monitoring of C(sp^3)-H fluorination reactions with an unprotected amino acid precursor, $AgNO_3$, Selectfluor® and **H-TEDA(BF$_4$)$_2$** 27

3.4.3 Reaction monitoring of photocatalytic C(sp^3)-H fluorination with acetophenone, **SF®** and **H-TEDA(BF$_4$)$_2$** 29

3.4.4 Reaction monitoring of photochemical C(sp^3)-H fluorination with **H-TEDA(BF$_4$)$_2$** 32

4 Diffusion ordered spectroscopy (DOSY) 34

4.1 Diffusion ordered spectroscopy (DOSY) measurements for aggregate-investigations in C(sp^3)-H fluorination reactions with SF® and **H-TEDA(BF$_4$)$_2$** loading 34

4.1.1 Introduction 34

4.1.2 General 36

4.1.3 NMR sample preparation 36

4.1.4 DOSY studies of **SF®** and **H-TEDA(BF$_4$)$_2$** using pure compounds at different concentrations and reaction mixtures with different **H-TEDA(BF$_4$)$_2$** loadings 37

4.1.5 DOSY studies of photochemical C(sp^3)-H fluorination reactions under standard conditions and with **H-TEDA(BF$_4$)$_2$** loading 42

5 Mechanistic study 47

5.1 Characterization of intermolecular interactions between **SF®** and **H-TEDA(BF$_4$)$_2$** with 1D and 2D NMR experiments 47

6 ^{1}H NMR, ^{13}C NMR and ^{19}F NMR Spectra 51

7 References 69

1 Experimental

1.1 General Comments

Unless otherwise specified, all reactions were performed using dried, deoxygenated solvents. Purifications were conducted by column chromatography with silica gel (Macherey Nagel 0.063–0.2 mm) and the solvents were used after receiving without further purification. Identities of products were confirmed by comparisons with the literature data as far as possible, using ^{1}H, ^{13}C and ^{19}F NMR spectra as well as HR-MS. Novel compounds were characterized by ^{1}H, ^{13}C, ^{19}F NMR as well as m.p. (solids), FT-IR and HR-MS. The reactions were followed and pure fractions from column chromatography were detected by TLC using silica gel pre-coated aluminum sheets (Macherey Nagel: Alugram Xtra SIL G UV254 Nr. 818333, thickness 0.2 mm). TLC plates were analyzed under a UV lamp (254 nm) and/or by potassium permanganate stain.

NMR spectra were recorded in $CDCl_3$ or CD_3CN on a Bruker Avance 400 (400 MHz for ^{1}H, 101 MHz for ^{13}C, 376 MHz for ^{19}F). For ^{1}H, ^{13}C and ^{19}F chemical shifts are presented in δ-scale as ppm (parts per million) with the residual solvent peak as the reference ($CDCl_3$: 7.26 ppm for ^{1}H and 117.7 ppm for ^{13}C; CD_3CN: 1.93 ppm for ^{1}H and 77.00 ppm for ^{13}C). For ^{19}F NMR, trifluorotoluene was added as an external standard and used as the reference peak (−63.38 ppm). If not stated, no reference was used. NMR yields were calculated based on ^{19}F NMR using either trifluorotoluene or pentafluorobenzene as an internal standard. MestReNova 6.0.2-5475 was used to process NMR spectra. The description of multiplicity that were used is as follows: s = singlet, br. s = broad singlet, d = doublet, dd = doublet of doublet, ddd = doublet of doublet of doublet, dm = doublet of multiplet, t = triplet, q = quartet, p = pentet, m = multiplet. FT-IR spectra were recorded on an Agilent Technologies Cary 630 FTIR Spectrometer. UV-vis absorption spectra were recorded on an Agilent Cary 100 UV/Vis spectrometer (the range of wavelength is 200 nm to 800 nm) and 0.10 mm thick 10 mm × 10 mm quartz cuvettes were used at 25 °C. HR-MS were recorded at the Central Analytical Department of the University of Regensburg and the spectra were measured on either a JOEL AccuTOF GCx instrument for electron ionization (EI) or an Agilent Q-TOF 6540 UHD instrument for electrospray ionization (ESI) and atmospheric-pressure chemical ionization (APCI).

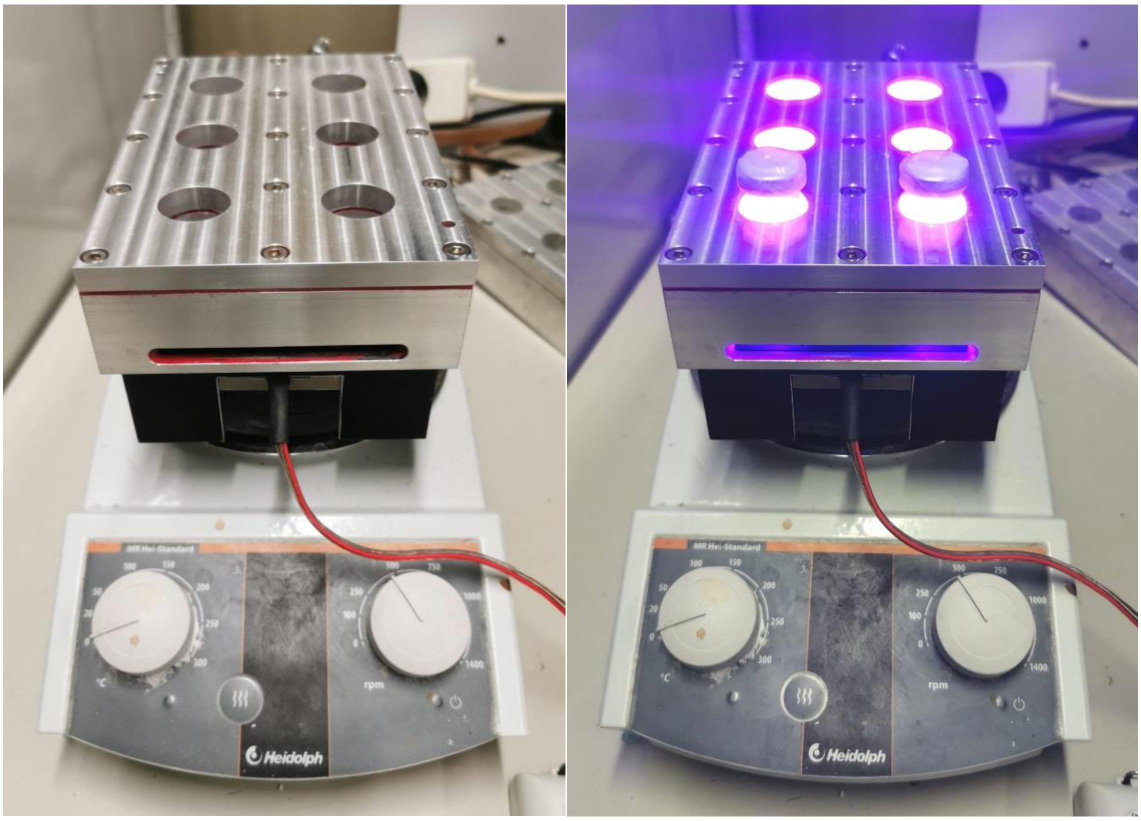

Figure S1. Typical set-up for photochemical reactions using purple LEDs 3.8 W (left) and 0.35 W (right).[1]

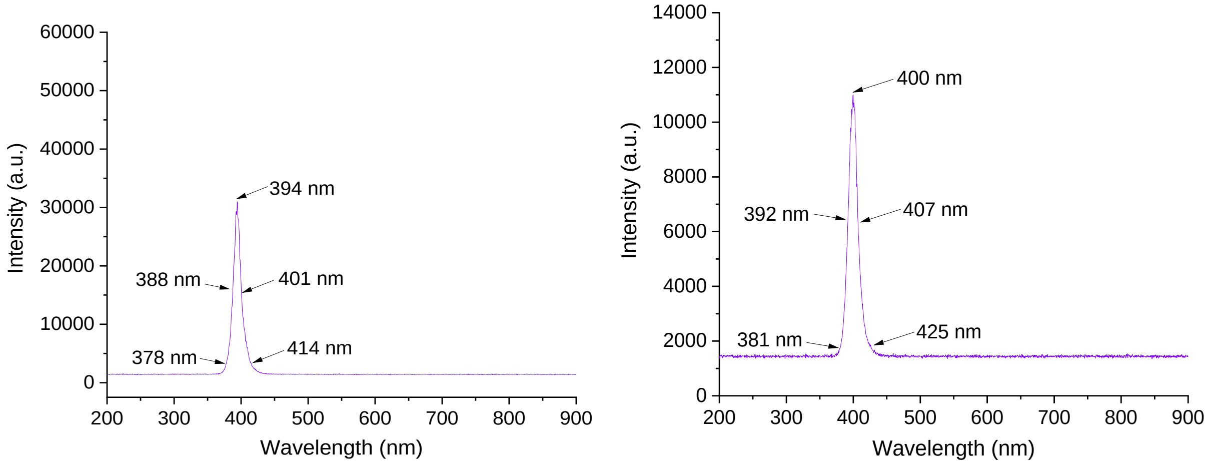

Figure S2. Relative LED intensities of 400 nm LEDs measured at a 30 cm distance directly above the LED. Input power of the higher intensity LED (left) = 3.8 W, input power of lower intensity LED (right) = 350 mW.

Specifications of LEDs: LED (left) = 3.8 W [LED Engine LZ4-40UB00-00U4 LEDs (λ = 395 nm, 14.8 V, 700 mA)], **:** LED (right) = 350 mW. [Edison EDEV-SLC1-03 LEDs (λ = 400 nm, 3.7 V, 700 mA)]. Optical power can be found at the manufacturer/distributor's website:

https://www.mouser.de/ProductDetail/ams-OSRAM/LZ4-40UB00-00U4?qs=VFUlUigmnHKqhqrhU8KwFQ%3D%3D

https://www.led-shop.com/?a=1007&lang=eng

(websites accessed 10.06.2024)

1.2 Fluorination of substrates with different amounts of additives

Table S1. Fluorination of butylphenyl benzoate with different amounts of additives.

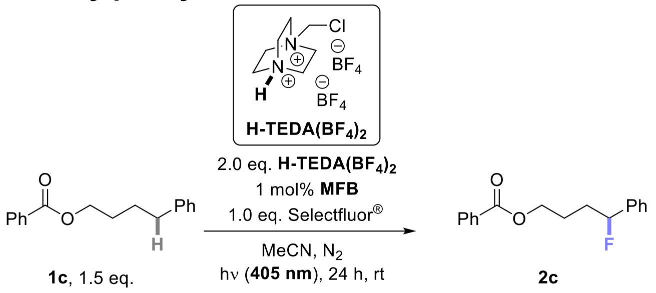

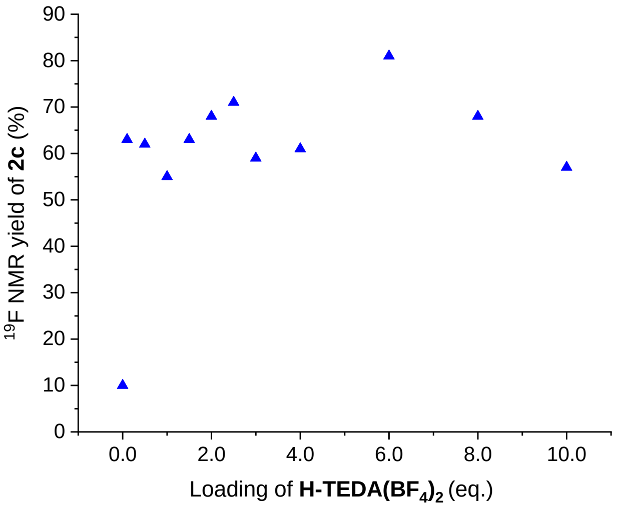

Entry	Additive / pK_a	NMR yield (%)
1	-	10
2	0.1 eq. **H−TEDA(BF$_4$)$_2$** / 2.3	63
3	0.5 eq. **H-TEDA(BF$_4$)$_2$** / 2.3	62
4	1.0 eq. **H-TEDA(BF$_4$)$_2$** / 2.3	55
5	1.5 eq. **H-TEDA(BF$_4$)$_2$** / 2.3	63
6	2.0 eq. **H-TEDA(BF$_4$)$_2$** / 2.3	68
7	2.5 eq. **H-TEDA(BF$_4$)$_2$** / 2.3	71
8	3.0 eq. **H-TEDA(BF$_4$)$_2$** / 2.3	59
9	4.0 eq. **H-TEDA(BF$_4$)$_2$** / 2.3	61
10	6.0 eq. **H-TEDA(BF$_4$)$_2$** / 2.3	81
11	8.0 eq. **H-TEDA(BF$_4$)$_2$** / 2.3	68
12	10.0 eq. **H-TEDA(BF$_4$)$_2$** / 2.3	57
13	2.0 eq. TFA / 3.4	45
14	4.0 eq. TFA / 3.4	24
15	6.0 eq. TFA / 3.4	19
16	2.0 eq. TEA−H·BF$_4$ / 9.0	63
17	6.0 eq. TEA−H·BF$_4$ / 9.0	46

For the references to pK_a values, see main manuscript (Refs. 20-28) and section S1.4.

*Note: Although in this specific case 6.0 eq. of **H−TEDA(BF$_4$)$_2$** gave the highest yield (entry 10), it was not the case in the majority of case studies that increasing **H−TEDA(BF$_4$)$_2$** gave a higher yield than 2.0 eq. Although in this case 0.1 eq. **H−TEDA(BF$_4$)$_2$** gave a yield only slightly lower than 2.0 eq. **H−TEDA(BF$_4$)$_2$** (entry 2), in other case studies 0.1 eq. **H−TEDA(BF$_4$)$_2$** gave very low / no product yield. Considering the loading of **H−TEDA(BF$_4$)$_2$** and the increasing mass intensity compared to the promotion of yield, we consider 2.0 or 2.5 eq. **H−TEDA(BF$_4$)$_2$** to be the best compromise (entries 6 and 7). Finally, 2.0 eq. was a superior loading than higher loadings for other protic additives (TFA or TEA−H·BF$_4$; entries 13 and 16).

Table S2. Fluorination of 4-methyl acetophenone with different amounts of additives.

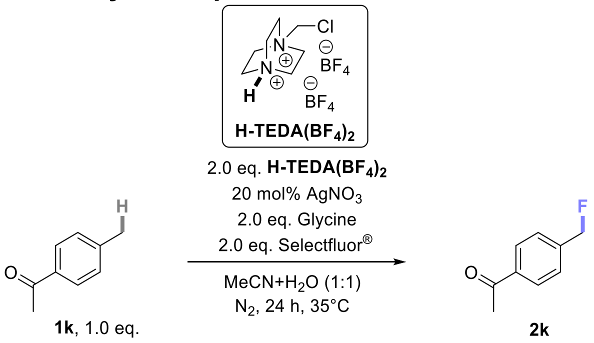

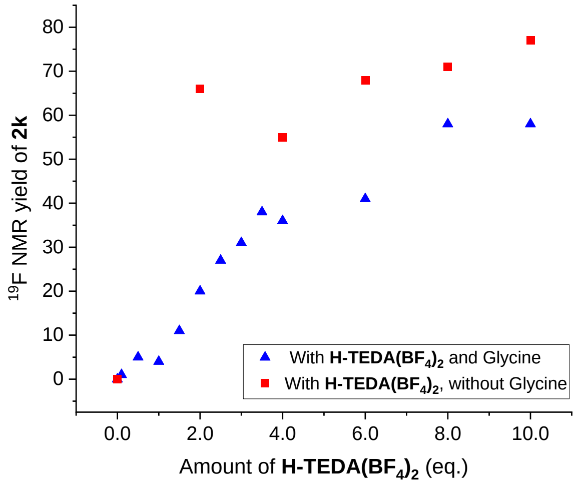

Entry	Additive	NMR yield (%)
1	-[a]	4
2	0.1 eq. **H-TEDA(BF$_4$)$_2$**	3
3	0.5 eq. **H-TEDA(BF$_4$)$_2$**	5
4	1.0 eq. **H-TEDA(BF$_4$)$_2$**	4
5	1.5 eq. **H-TEDA(BF$_4$)$_2$**	11
6	2.0 eq. **H-TEDA(BF$_4$)$_2$**	20
7	2.5 eq. **H-TEDA(BF$_4$)$_2$**	27
8	3.0 eq. **H-TEDA(BF$_4$)$_2$**	31
9	3.5 eq. **H-TEDA(BF$_4$)$_2$**	38

10	4.0 eq. **H-TEDA$(BF_4)_2$**	36
11	6.0 eq. **H-TEDA$(BF_4)_2$**	41
12	8.0 eq. **H-TEDA$(BF_4)_2$**	58
13	10.0 eq. **H-TEDA$(BF_4)_2$**	58
14	0.1 eq. **H-TEDA$(BF_4)_2$**[a,b]	0
15	2.0 eq. **H-TEDA$(BF_4)_2$**[a,b]	66
16	4.0 eq. **H-TEDA$(BF_4)_2$**[a,b]	55
17	6.0 eq. **H-TEDA$(BF_4)_2$**[a,b]	68
18	8.0 eq. **H-TEDA$(BF_4)_2$**[a,b]	71
19	10.0 eq. **H-TEDA$(BF_4)_2$**[a,b]	77
20	0 eq. **H-TEDA$(BF_4)_2$**[a,b]	0
21	2.0 eq. **H-TEDA$(BF_4)_2$**[a,b,c]	0
22	2.0 eq. TFA	0
23	4.0 eq. TFA	0
24	2.0 eq. TFA[b]	0
25	4.0 eq. TFA[b]	0
26	2.0 eq. Py-H BF_4	Traces
27	2.0 eq. Py-H BF_4[b]	18

[a] Reaction time 48 h. [b] Without glycine. [c] Without $AgNO_3$

*Note: In the presence of glycine (entries 1-13), an increasing loading of **H−TEDA$(BF_4)_2$** correlated with increasing product yield. However, in the absence of glycine (entries 14-19) this was not necessarily the case and 2.0 eq. of **H−TEDA$(BF_4)_2$** was deemed the optimal balance between yield benefit and added mass intensity.

Table S3. Fluorination of butylphenyl 4-fluorobenzoate with different amounts of additives.

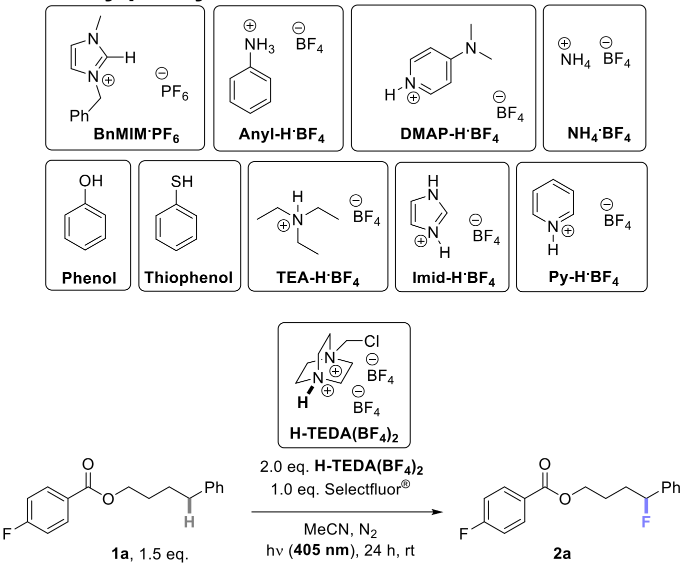

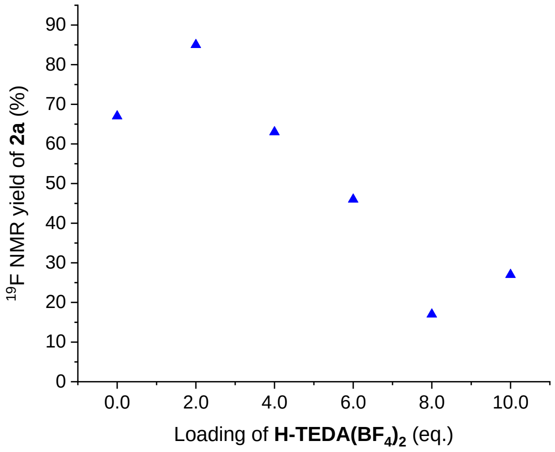

Entry	Fluorinating agent	Additive	NMR yield (%)
1	Selectfluor®	-	67
2	Selectfluor®	1.0 eq. **H-TEDA(BF$_4$)$_2$**	64
3	Selectfluor®	2.0 eq. **H-TEDA(BF$_4$)$_2$**	85
4	Selectfluor®	4.0 eq. **H-TEDA(BF$_4$)$_2$**	63
5	Selectfluor®	6.0 eq. **H-TEDA(BF$_4$)$_2$**	46
6	Selectfluor®	8.0 eq. **H-TEDA(BF$_4$)$_2$**	17
7	Selectfluor®	10.0 eq. **H-TEDA(BF$_4$)$_2$**	27
8	Selectfluor II	-	36
9	Selectfluor II	2.0 eq. **H-TEDA(BF$_4$)$_2$**	64
10	Selectfluor II	2.0 eq. TFA	43
11	NFSI	-	0
12	NFSI	2.0 eq. **H-TEDA(BF$_4$)$_2$**	27
13	NFSI	2.0 eq. TFA	14
14	Selectfluor®	2.0 eq. Phenol	0
15	Selectfluor®	2.0 eq. Thiophenol	0
16	Selectfluor®	2.0 eq. $NH_4{\cdot}BF_4$	0
17	Selectfluor®	2.0 eq. BnMIM·PF_6	0
18	Selectfluor®	2.0 eq. TEA-H·BF_4	65
19	Selectfluor®	2.0 eq. Py-H·BF_4	62
20	Selectfluor®	2.0 eq. DMAP-H·BF_4	0
21	Selectfluor®	2.0 eq. Anyl-H·BF_4	0
22	Selectfluor®	2.0 eq. Imid-H·BF_4	65
23	Selectfluor®	2.0 eq Acetic acid	59
24	Selectfluor®	0.1 eq. TFA	65
25	Selectfluor®	2.0 eq. TFA	90
26	Selectfluor®	4.0 eq. TFA	22
27	Selectfluor®	2.0 eq. TFA[a]	32

28	Selectfluor®	2.0 eq. TFA[b]	0
29	Selectfluor®	2.0 eq. $AlCl_3$	0
30	Selectfluor®	2.0 eq. $B(OPh)_3$	0
31	Selectfluor®	2.0 eq. $Zn(OTf)_2$	traces
32	Selectfluor®	2.0 eq. $Cu(OTf)_2$	0

[a] Under air. [b] Without irradiation.

Note: In the reaction of **1a**, 2.0 eq. of promotor proved most beneficial both for **H−TEDA(BF$_4$)$_2$** and for TFA as protic additives (entries 3 and 25). **H−TEDA(BF$_4$)$_2$** was a more effective promotor for different fluorination agents than TFA (entries 9 and 12).

Table S4. Fluorination of butylphenyl acetate with different amounts of additives.

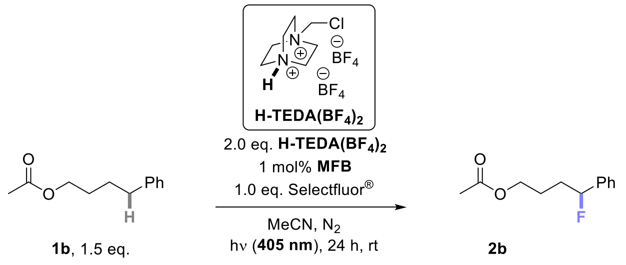

Entry	Additive (2.0 eq.) / pK_a in DMSO	NMR yield (%)
1	-	19
2	-[a]	0
3	-[b]	0
4	H_2O / 31.4	0
5	MeOH / 29	0
6	BnMIM·PF_6 / 21.6	0
7	Phenol / 18	0
8	**H-TEDA(BF$_4$)$_2$ / 2.3**	64
9	TFA / 3.4	43
10	TEA-H·BF_4 / 9.0	62
11	Py-H·BF_4 / 3.4	36
12	Imid-H·BF_4 / 6.4	60
13	Acetic acid / 12.3	59
14	TFA[a,b] / 3.4	0
15	MsOH / 1.6	0
16	MsOH[c] / 1.6	16
17	CSA / 1.2	0
18	TsOH / 0.9	0

19	TfOH / 0.3	0

[a] Without irradiation. [b] Without the photocatalyst - **MFB**. [c] 5.5 mg (0.05 mmol, 0.28 eq.) MsOH was added, giving a reaction mixture with the same pH as a reaction mixture in presence of 2.0 eq. **H-TEDA(BF_4)$_2$**. For the references to pK_a values, see main manuscript (Refs. 20-28) and section S1.4.

Note: For the reaction forming **2b**, 2.0 eq. of **H−TEDA(BF_4)$_2$** gave the best result of the different protic additives (entry 8). Acetic acid, TEA−H·BF_4 and Imid−H·BF_4 gave respectable yields (enties 10,12 and 13).

1.3 Triplicate results

Table S5. Thermal catalytic fluorination of ibuprofen derivative without and with promotor.

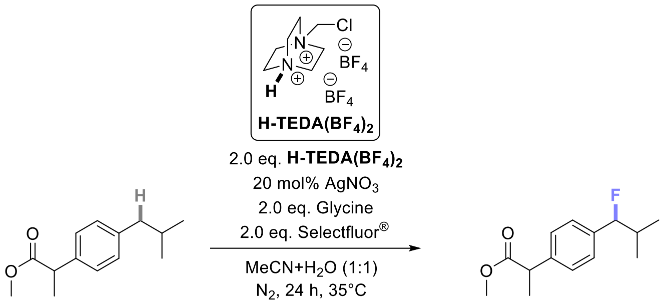

1l, 1.0 eq. **2l**

Entry	Conditions	NMR yield (%) Rep. 1	NMR yield (%) Rep. 2	NMR yield (%) Rep. 3
1	Literature conditions[2]	12	15	16
2	Literature conditions plus 2.0 eq. **H-TEDA(BF_4)$_2$**	54	49	52

Table S6. Thermal catalytic fluorination of 4-methylacetophenone without and with promotor.

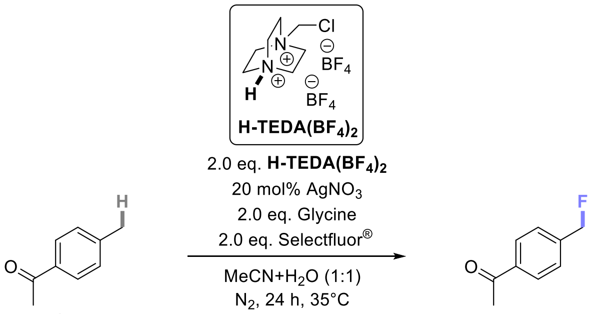

1k, 1.0 eq. **2k**

Entry	Conditions	NMR yield (%) Rep. 1	NMR yield (%) Rep. 2	NMR yield (%) Rep. 3
1	Literature conditions[2]	9	3	13
2	Literature conditions plus 2.0 eq. **H-TEDA(BF_4)$_2$**	21	18	22

Table S7. Photocatalytic fluorination of cyclododecane without and with promotor.

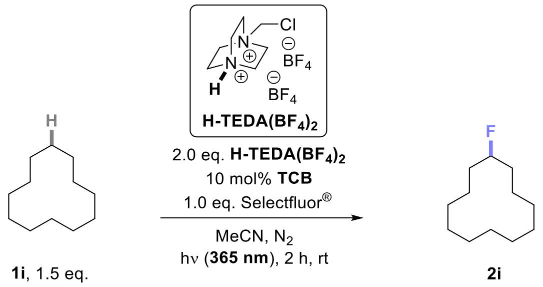

Entry	Conditions	NMR yield (%) Rep. 1	NMR yield (%) Rep. 2	NMR yield (%) Rep. 3
1	Literature conditions[3]	76	74	72
2	Literature conditions plus 2.0 eq. **H-TEDA(BF$_4$)$_2$**	94	90	91

Table S8. Photocatalytic fluorination of hexan-2-one without and with promotor.

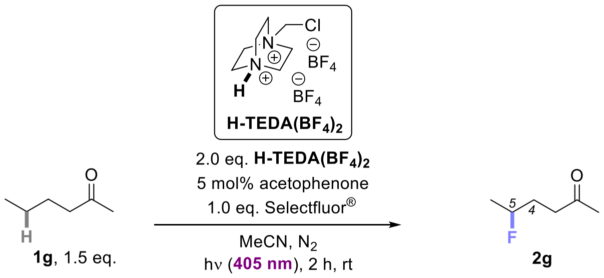

Entry	Conditions	NMR yield (%) Rep. 1	NMR yield (%) Rep. 2	NMR yield (%) Rep. 3
1	Literature conditions[4]	67	72	68
2	Literature conditions plus 2.0 eq. **H-TEDA(BF$_4$)$_2$**	98	96	97

Table S9. Photocatalytic fluorination of adamantane without and with promotor.

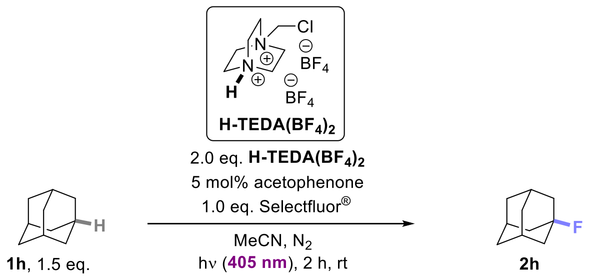

Entry	Conditions	NMR yield (%) Rep. 1	NMR yield (%) Rep. 2	NMR yield (%) Rep. 3
1	Literature conditions[4]	53	50	51

2	Literature conditions plus 2.0 eq. **H-TEDA(BF₄)₂**	74	70	68

Table S10. Photocatalytic fluorination of protected leucine without and with promotor.

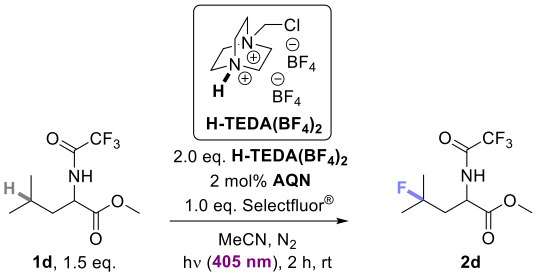

Entry	Conditions	NMR yield (%) Rep. 1	NMR yield (%) Rep. 2	NMR yield (%) Rep. 3
1	Literature conditions[5]	33	34	30
2	Literature conditions plus 2.0 eq. **H-TEDA(BF₄)₂**	44	55	56

Table S11. Photocatalytic fluorination of amyl benzoate without and with promotor.

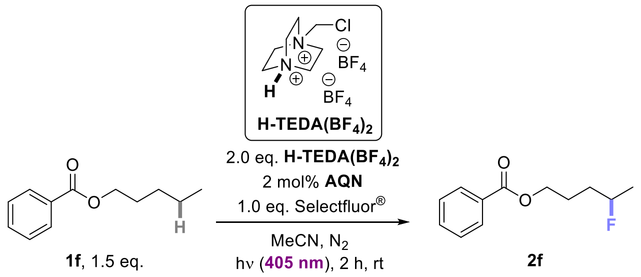

Entry	Conditions	NMR yield (%) Rep. 1	NMR yield (%) Rep. 2	NMR yield (%) Rep. 3
1	Literature conditions[5]	55	62	64
2	Literature conditions plus 2.0 eq. **H-TEDA(BF₄)₂**	82	82	81

Table S12. Photocatalytic fluorination of 1,10-dibromodecane without and with promotor.

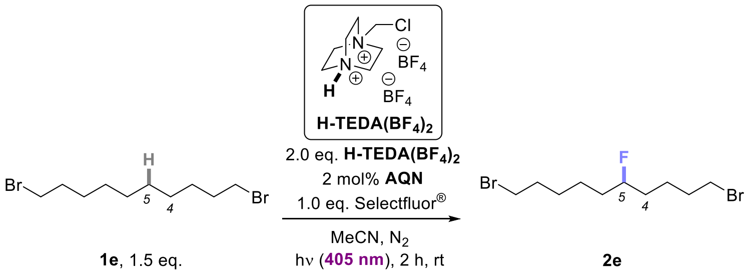

Entry	Conditions	NMR yield (%) Rep. 1	NMR yield (%) Rep. 2	NMR yield (%) Rep. 3
1	Literature conditions[5]	41	52	48
2	Literature conditions plus 2.0 eq. **H-TEDA(BF$_4$)$_2$**	62	72	69

Table S13. Photocatalytic fluorination of 1-phenylpentyl 4-fluorobenzoate without and with promotor.

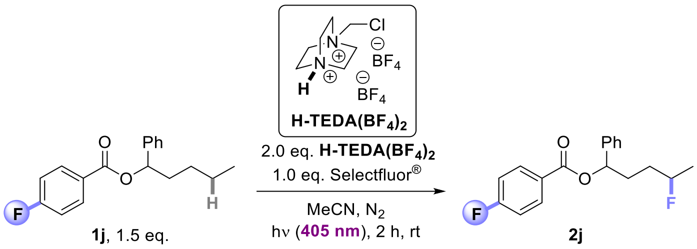

Entry	Additive	NMR yield (%)
1	-	38
2	1.0 eq. **H-TEDA(BF$_4$)$_2$**	61
3	2.0 eq. **H-TEDA(BF$_4$)$_2$**	65
4	2.0 eq. TFA	74
5	4.0 eq. TFA	54

Note: In the reaction forming **2j**, 2.0 eq. **H−TEDA(BF$_4$)$_2$** was superior to using 1.0 eq. or using none (entries 1-3). In this case, 2.0 eq. TFA gave a superior result, and increasing to 4.0 eq. TFA degraded the yield (entries 4-5).

1.4 Calculating pK_a of **H-TEDA(BF$_4$)$_2$** in H_2O

A pH titration method was used as appropriate for weak organic acids. The pH was measured with a pH meter (Schott CG 842), calibrated with solutions of known pH. The probe was cleaned with distilled water after each measurement. A 0.2 M solution of **H-TEDA(BF$_4$)$_2$** (672 mg, 2.0 mmol, in 10 mL water) was prepared. NaOH was used as a base and also 0.2 M solution of NaOH (80 mg, 2.0 mmol, in 10 mL water) was prepared. The pH of the **H-TEDA(BF$_4$)$_2$** solution was measured before the addition of the base. Then, to the stirring **H-TEDA(BF$_4$)$_2$** solution, 0.1 eq. (1.0 mL of 0.2 M solution) of NaOH was added slowly and pH of the solution was measured after each time 0.1 eq. NaOH solution was added. Thus, the titration curve of **H-TEDA(BF$_4$)$_2$** was obtained and pK_a value estimated from the pH value at the half-equivalence point = 2.3.

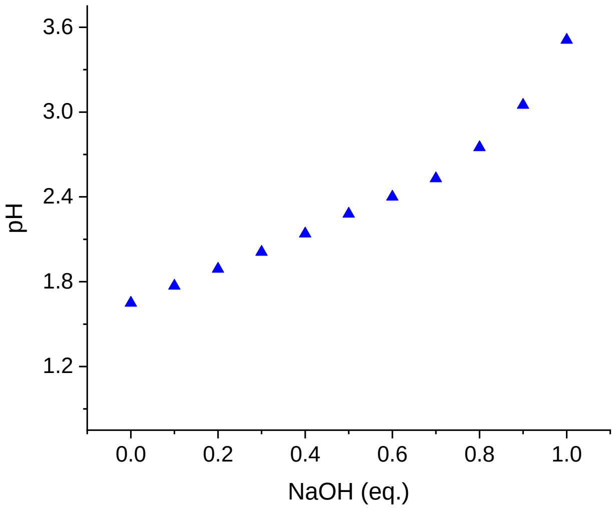

Figure S3. pH titration of **H-TEDA(BF$_4$)$_2$** vs (aq.) 0.2 M NaOH.

1.5 Isolation of **H-TEDA(BF$_4$)$_2$** After Fluorination Reactions

Upon completion of fluorination reaction, diethyl ether was added to the reaction mixture, and instant precipitation of **H-TEDA(BF$_4$)$_2$** as a white powder was observed. The mixture was filtered and the filtered powder was washed with more diethyl ether. The powder was dried, and it was confirmed pure by ^{1}H and ^{19}F NMR. This **H-TEDA(BF$_4$)$_2$**, obtained consistently in quantitative yield, is used for further fluorination reactions.

1.6 Recycling of **H-TEDA(BF$_4$)$_2$** After Fluorination Reactions

The efficient recycling of additives in chemical reactions holds paramount importance in sustainable synthesis strategies. The recycling of additives represents a fundamental aspect of green chemistry, aiming to reduce waste generation and enhance the sustainability of chemical processes. Among various additives, **H-TEDA(BF$_4$)$_2$** has garnered significant attention due to its versatile properties and potential for recycling in multiple reactions. Herein, we elucidate the recyclability of **H-TEDA(BF$_4$)$_2$** through a comprehensive series of experiments, showcasing its effectiveness in promoting diverse fluorination methods while maintaining high yields without diminishing efficiency. First, we synthesized **H-TEDA(BF$_4$)$_2$** via the fluorination of adamantanol in a photocatalytic reaction, yielding the desired additive in quantitative yield and subsequently employed it in various reactions, isolating it after each cycle and utilizing it for subsequent reactions. The recycling efficacy was demonstrated by maintaining quantitative yields of **H-TEDA(BF$_4$)$_2$** throughout multiple cycles, underscoring its sustainable application as an additive. Subsequently, **H-TEDA(BF$_4$)$_2$** was utilized in successive reactions, including photocatalytic fluorination of butylphenyl acetate, thermal fluorination of 4-methyl acetophenone, and photosensitization auxiliary fluorination of butylphenyl 4-fluorobenzoate. Each reaction was performed using 2.0 equivalents of **H-TEDA(BF$_4$)$_2$**, resulting in the isolation of 3.0 equivalents after completion, due to the conversion of 1.0 equivalent of Selectfluor® to **H-TEDA(BF$_4$)$_2$**. The isolated **H-TEDA(BF$_4$)$_2$** was then recycled for subsequent reactions, maintaining quantitative yields throughout multiple cycles.

The practicality of this recycling method was also evident in its application to the photocatalytic fluorination of 2-hexanone using 5 mol% acetophenone, where **H-TEDA(BF$_4$)$_2$** exhibited consistent performance over five consecutive reactions. Notably, the yield of the desired products remained constant (Run 1 = 97%,

Run 2 = 96%, Run 3 = 95%, Run 4 = 94%, Run 5 = 95%), and **H-TEDA(BF$_4$)$_2$** was isolated in excellent yields (Run 1 = 148%, Run 2 = 147%, Run 3 = 149%, Run 4 = 147%, Run 5 = 147%) after each reaction. In contrast, attempts to recycle other additives such as TEA−H·BF$_4$ and TFA proved futile. After the standard workup of the reaction with TEA−H·BF$_4$ (attempting to crash out a solid in the same way as **H-TEDA(BF$_4$)$_2$**), 95% of this additive could be detected in the organic phase together with the desired product (**2b**). Therefore, in contrast to the **H-TEDA(BF$_4$)$_2$**, TEA−H·BF$_4$ requires column chromatography to isolate it from the reaction crude (if this is even possible, since there is no chromophore for TLC) rendering it unsuitable to recycle for subsequent reactions. Similarly, TFA, being volatile, was separated into organic part of the reaction crude with only 30% NMR yield, hindering its recycling potential. Although TEA−H·BF$_4$ can compete with **H-TEDA(BF$_4$)$_2$** in promotion performance in some cases, it can only be used as a single-use stoichiometric promotor (moreover, it costs ≥2 € / g according to two major suppliers, Sigma-Aldrich: product number 242144-25G https://www.sigmaaldrich.com/DE/de/product/aldrich/242144 ; TCI chemicals product number T0983 25 G https://www.tcichemicals.com/AT/de/search/?text=429-06-1)

In summary, the inability to recycle other additives emphasizes the unique recyclability and practicality of **H-TEDA(BF$_4$)$_2$** in chemical transformations. Moreover, the generality of **H-TEDA(BF$_4$)$_2$** as a promotor is clear, since in most reaction cases tested TFA and TEA−H·BF$_4$ performed inferiorly to **H-TEDA(BF$_4$)$_2$** (see Tables S1, S2, S4) or even gave no reaction at all.

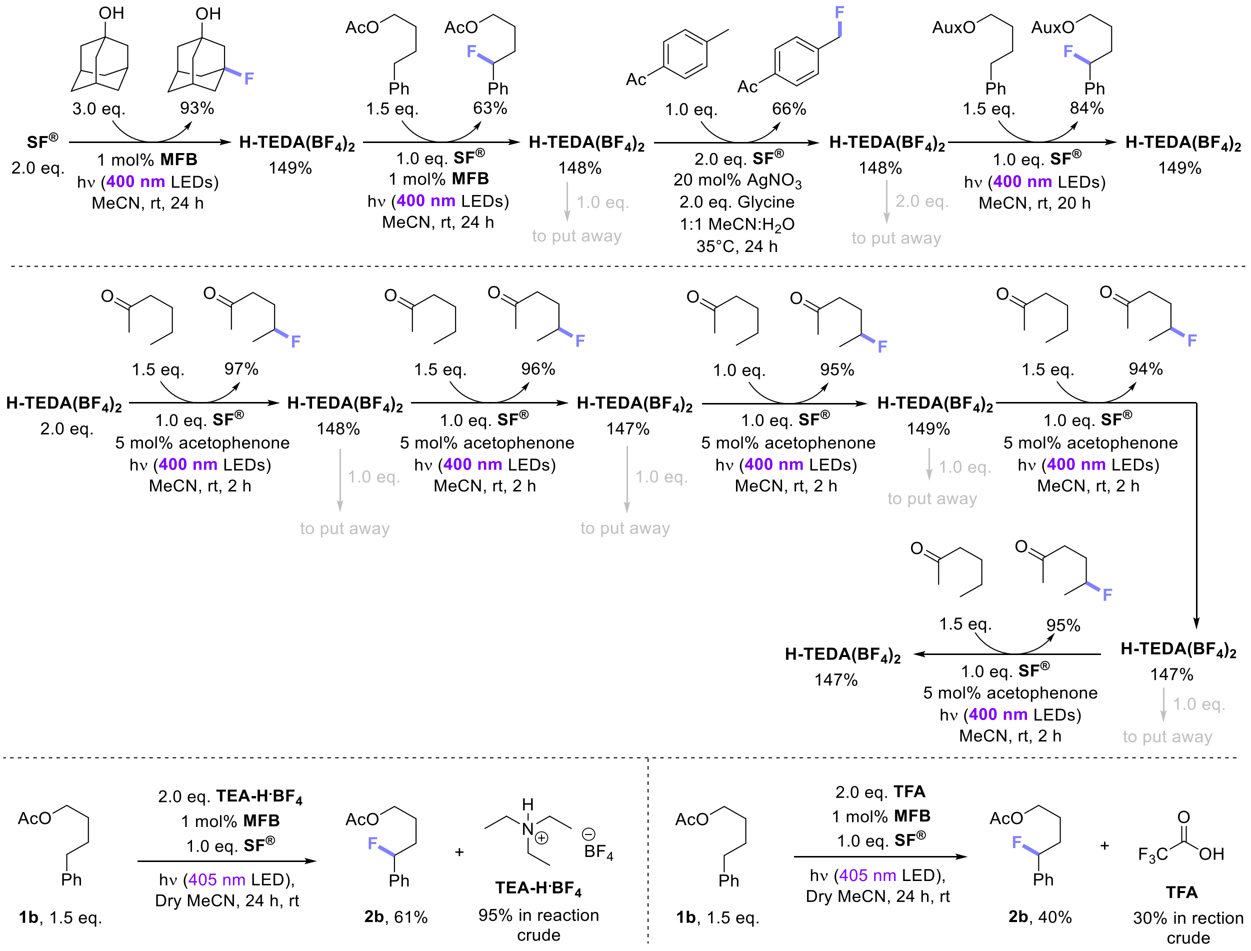

Figure S4. Recyclability assessment of **H-TEDA(BF$_4$)$_2$** and other additives.

1.7 Synthesis of **H-TEDA(BF$_4$)$_2$** from DABCO

The first step - alkylation: 1,4-diazabicyclo[2.2.2]octane (DABCO, also known as triethylenediamine (**TEDA**)) (5.61 g, 50.0 mmol, 1.0 eq.) was dissolved in DCM (100 mL) and refluxed under 40 °C for 24 h. The reaction mixture was concentrated *in vacuo* and the crude product was taken through to the second step.

The second step – counterion exchange and protonation: the crude product from the first step (entire quantity from the step above) was dissolved in acetonitrile (50 mL) and a solution of tetrafluoroboric acid (48 wt.% in H_2O) (20.0 mL, 150 mmol, 3.0 eq.) was added to the solution. The reaction mixture was stirred for 1 h. A white solid precipitated that was filtered and dried *in vacuo* to provide the desired product - **H-TEDA(BF$_4$)$_2$** (quantitative yield, 16.81 g, 50.0 mmol) - pure by ^{1}H and ^{19}F NMR.

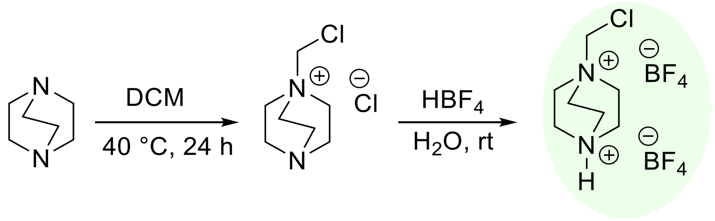

1-(chloromethyl)-1,4-diazabicyclo[2.2.2]octane-1,4-diium ditetrafluoroborate (H-TEDA(BF$_4$)$_2$)

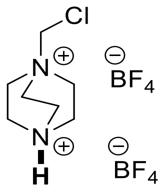

Yield: quant.; white solid; IR (neat) ν (cm^{-1}): 3179, 3060, 3012, 1480, 1409, 1338, 1290, 1036, 943, 895, 850, 798, 764, 731; ^{1}H NMR (400 MHz, CD_3CN) δ 7.45 (br. s, 1H), 5.20 (s, 2H), 3.82 (dt, *J* = 15.0, 6.7 Hz, 12H) ppm; ^{13}C NMR (101 MHz, CD_3CN) δ 70.1, 51.0, 45.1 ppm; ^{19}F NMR (377 MHz, CD_3CN) δ −150.0 (s), −150.1 (s) ppm; HRMS (ESI) (*m/z*) [M-H]$^+$: exact mass calcd. for $C_7H_{14}ClN_2^+$: 161.0840; found: 161.0837.

2 Synthesis

2.1 Synthesis of Starting Materials

General Procedure 1: Esterification

To a solution of DMAP (0.1 eq.), Et_3N (1.5 eq.) in DCM (0.2 M) was added the alcohol substrate (1.0 eq.) and the mixture cooled to 0°C. Benzoyl chloride (1.2 eq.) was added dropwise to the reaction mixture and the reaction was stirred overnight at room temperature (rt). The solution was quenched with H_2O and extracted three times with DCM (20 mL). Combined organic layers were dried over $NaSO_4$, filtered, and concentrated *in vacuo*. The residue was purified by column chromatography using silica gel and the specified eluent to yield the desired ester.

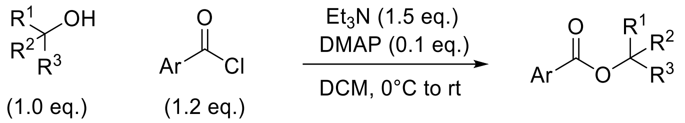

4-Phenylbutyl benzoate (1c)

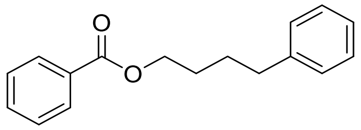

According to the **General Procedure 1**. Yield: 2.44 g, 96%; colorless liquid; ^{1}H NMR (400 MHz, $CDCl_3$) δ 8.14 – 8.07 (m, 2H), 7.63 – 7.55 (m, 1H), 7.47 (dd, *J* = 10.6, 4.7 Hz, 2H), 7.38 – 7.30 (m, 2H), 7.25 (dd, *J* = 10.1, 4.1 Hz, 3H), 4.40 (t, *J* = 6.2 Hz, 2H), 2.74 (t, *J* = 7.1 Hz, 2H), 2.02 – 1.72 (m, 4H) ppm.

Data are consistent with the literature.[1]

Methyl 2-(4-isobutylphenyl)propanoate (1l)

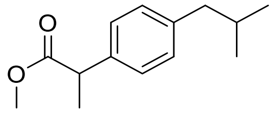

Prepared according to a literature procedure.[6] To the mixture of Ibuprofen (5.00 mmol, 1.0 eq.) and DMAP (0.50 mmol, 0.1 eq.) in 10 mL DCM was added methanol (15.00 mmol, 3.0 eq.) at 0 °C. The resulting reaction mixture was stirred for 5 min. DCC (5.50 mmol, 1.1 eq.) was added next and the reaction mixture stirred at 0 °C for 5 more minutes. The reaction was allowed to warm to rt and stirred for 3 h. The resulting precipitates were filtered, the filtrate treated with diluted HCl (20 mL, 0.5 M) and the product was extracted three times with DCM (40 mL). The organic layers were combined, dried over $MgSO_4$, filtered and concentrated *in vacuo*. The residue was purified by column chromatography using 5% EtOAc in pentane to afford the desired product.

Yield: 1.05 g, 95%; colorless liquid; ^{1}H NMR (400 MHz, $CDCl_3$) δ 7.20 (d, *J* = 8.1 Hz, 2H), 7.11 (d, *J* = 8.1 Hz, 2H), 3.70 (q, *J* = 7.2 Hz, 1H), 3.67 (s, 3H), 2.46 (d, *J* = 7.2 Hz, 2H), 1.86 (td, *J* = 13.6, 6.8 Hz, 1H), 1.51 (d, *J* = 7.2 Hz, 3H), 0.91 (d, *J* = 6.6 Hz, 6H) ppm.

Data are consistent with the literature.[1]

4-Phenylbutyl 4-fluorobenzoate (1a)

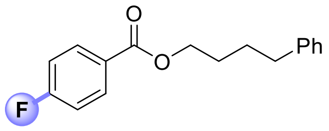

According to the **General Procedure 1**. Yield: 1.28 g, 94%; colorless liquid; ^{1}H NMR (400 MHz, $CDCl_3$) δ 8.12 – 8.02 (m, 2H), 7.36 – 7.27 (m, 2H), 7.25 – 7.18 (m, 3H), 7.16 – 7.08 (m, 2H), 4.35 (dd, *J* = 8.4, 4.0 Hz, 2H), 2.70 (t, *J* = 7.1 Hz, 2H), 1.88 – 1.75 (m, 4H) ppm; ^{19}F NMR (377 MHz, $CDCl_3$) δ −106.4 (tt, *J* = 8.5, 5.5 Hz) ppm.

Data are consistent with the literature.[1]

4-Phenylbutyl acetate (1b)

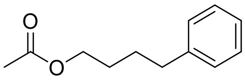

Prepared according to the **General Procedure 1**. Yield: 1.88 g, 98%, colorless liquid; ^{1}H NMR (400 MHz, $CDCl_3$) δ 7.34 – 7.16 (m, 5H), 4.11 (dd, *J* = 8.6, 4.0 Hz, 2H), 2.66 (t, *J* = 7.2 Hz, 2H), 2.05 (s, 3H), 1.78 – 1.56 (m, 4H).
^{1}H NMR data are consistent with the literature.[1]

1-phenylpentyl 4-fluorobenzoate (1j)

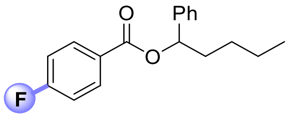

Prepared according to the **General Procedure 1**. Yield: 2.12 g, 95%; slightly yellow solid; IR (neat) ν (cm^{-1}): 2960, 2863, 1718, 1603, 1506, 1454, 1413, 1264, 1152, 1088, 1044, 962, 910, 854, 801, 764, 701; ^{1}H NMR (400 MHz, $CDCl_3$) δ 8.32 – 8.20 (m, 2H), 7.59 (dd, *J* = 5.2, 3.4 Hz, 2H), 7.55 – 7.48 (m, 2H), 7.48 – 7.41 (m, 1H), 7.30 – 7.22 (m, 2H), 6.15 (dd, *J* = 7.5, 6.4 Hz, 1H), 2.31 – 2.15 (m, 1H), 2.14 – 2.00 (m, 1H), 1.63 – 1.43 (m, 4H), 1.06 (t, *J* = 7.0 Hz, 3H) ppm; ^{13}C NMR (101 MHz, $CDCl_3$) δ 167.0 (s), 164.9 (s), 164.5 (s), 140.8 (s), 132.2 (d, *J* = 9.3 Hz), 128.5 (s), 127.9 (s), 126.8 (d, *J* = 3.0 Hz), 126.5 (s), 115.5 (d, *J* = 22.0 Hz), 77.0 (s), 36.3 (s), 27.7 (s), 22.5 (s), 14.0 (s) ppm; ^{19}F NMR (377 MHz, $CDCl_3$) δ −106.3 (tt, *J* = 8.5, 5.5 Hz) ppm; HRMS (ESI) (*m/z*) $[M]^+$: exact mass calcd. for $C_{18}H_{19}FO_2$: 286.1369; found: 286.1365.

Methyl (2,2,2-trifluoroacetyl)leucinate (1d)

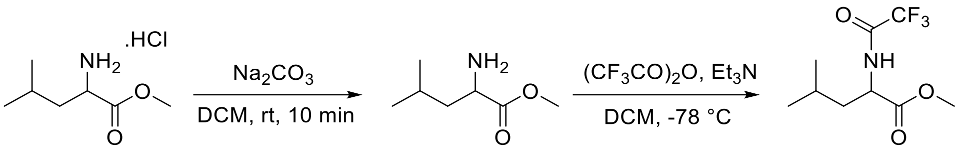

Prepared according to a literature procedure:[7]
Step 1: *L*-Leucine methyl ester hydrochloride salt (10.00 mmol) was added into a separatory funnel with DCM (20 mL) followed by the addition of sat. aq. Na_2CO_3 (50 mL). The reaction mixture was vigorously shaken for a few minutes. The mixture was extracted with DCM (3 x 20 mL). Organic layers were collected, dried over $MgSO_4$, filtered, and concentrated under reduced pressure to obtain the neutralized product that was used in the next step without purification. Step 2: Trifluoroacetic anhydride (10 mmol, 1.0 eq.) was added dropwise (over 5 min) to a solution of neutralized *L*-leucine methyl ester (10 mmol, 1.0 eq.) and Et_3N (11 mmol, 1.1 eq.) in DCM (20 mL) at −78 °C. After complete addition, the reaction mixture was stirred at −78 °C for 3 h and quenched with sat. aq. $NaHCO_3$ solution (10 mL). The mixture was allowed to warm to rt and was extracted with DCM (3 x 20 mL). The combined organic layers were washed with water (30 mL), dried over $MgSO_4$, and filtered. The solution was concentrated and purified

by silica gel flash column chromatography (50% EtOAc in pentane) to obtain the desired product. Yield: 2.30 g, 95%; colorless liquid; ^{1}H NMR (400 MHz, $CDCl_3$) δ 7.27 (d, *J* = 7.1 Hz, 1H), 4.88 (td, *J* = 8.5, 5.2 Hz, 1H), 3.99 (s, 3H), 2.00 – 1.80 (m, 3H), 1.17 (dd, *J* = 6.3, 2.1 Hz, 6H) ppm; ^{13}C NMR (101 MHz, $CDCl_3$) δ 171.9 (s), 156.9 (q, *J* = 37.6 Hz), 115.6 (q, *J* = 287.6 Hz), 52.7 (s), 51.1 (s), 41.1 (s), 24.7 (s), 22.5 (s), 21.7 (s) ppm; ^{19}F NMR (377 MHz, $CDCl_3$) δ −76.5 (s) ppm; HRMS (ESI) (*m/z*) $[M+NH_4]^+$: exact mass calcd. for $C_9H_{14}F_3NO_3$: 259.1270; found: 259.1260.
The data are consistent with the literature.[5]

2.2 Synthesis of Additives

Triethylammonium tetrafluoroborate (TEA-H·BF$_4$)

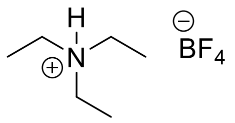

^{1}H NMR (400 MHz, D_2O) δ 4.79 (s, 1H), 3.27 (q, *J* = 7.3 Hz, 2H), 1.35 (t, *J* = 7.4 Hz, 3H) ppm; ^{13}C NMR (101 MHz, D_2O) δ 46.8, 8.3 ppm; ^{19}F NMR (377 MHz, D_2O) δ −150.3 (s), −150.4 (s) ppm; HRMS (ESI) (*m/z*) $[M]^+$: exact mass calcd. for $C_6H_{16}N^+$: 102.1277; found: 102.1278.

Pyridin-1-ium tetrafluoroborate (Py-H·BF$_4$)

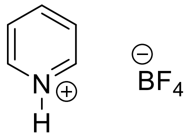

^{1}H NMR (400 MHz, D_2O) δ 8.79 (d, *J* = 5.3 Hz, 2H), 8.64 (tt, *J* = 7.9, 1.5 Hz, 1H), 8.09 (t, *J* = 7.1 Hz, 2H) ppm; ^{13}C NMR (101 MHz, D_2O) δ 147.3, 141.1, 127.5 ppm; ^{19}F NMR (377 MHz, D_2O) δ −150.4 (s), −150.4 (s) ppm; HRMS (ESI) (*m/z*) $[M]^+$: exact mass calcd. for $C_5H_6N^+$: 80.0495; found: 80.0494.

1*H*-imidazol-3-ium tetrafluoroborate (Imid-H·BF$_4$)

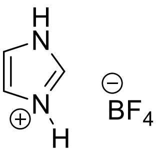

^{1}H NMR (400 MHz, D_2O) δ 8.72 (s, 1H), 7.52 (s, 2H) ppm; ^{13}C NMR (101 MHz, D_2O) δ 133.51, 119.01 ppm; ^{19}F NMR (377 MHz, D_2O) δ −150.3 (s), −150.4 (s) ppm; HRMS (ESI) (*m/z*) $[M]^+$: exact mass calcd. for $C_3H_5N_2^+$: 69.0447; found: 69.0446.

Benzenaminium tetrafluoroborate (Anyl-H·BF$_4$)

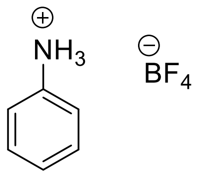

^1H NMR (400 MHz, D_2O) δ 7.54 – 7.44 (m, 3H), 7.41 – 7.35 (m, 2H) ppm; ^{13}C NMR (101 MHz, D_2O) δ 130.1, 129.7, 129.3, 122.9 ppm; ^{19}F NMR (377 MHz, D_2O) δ −150.3 (s), −150.4 (s) ppm; HRMS (ESI) (*m/z*) $[M]^+$: exact mass calcd. for $C_6H_8N^+$: 94.0651; found: 94.0651.

4-(Dimethylammonio)pyridin-1-ium ditetrafluoroborate (DMAP-H·BF$_4$)

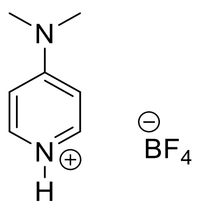

^1H NMR (400 MHz, D_2O) δ 8.00 (d, *J* = 7.7 Hz, 2H), 6.84 (d, *J* = 7.8 Hz, 2H), 3.18 (s, 6H) ppm; ^{13}C NMR (101 MHz, D_2O) δ 157.4, 138.2, 106.7, 39.3 ppm; ^{19}F NMR (377 MHz, D_2O) δ −150.3 (s), −150.4 (s) ppm; HRMS (ESI) (*m/z*) $[M]^+$: exact mass calcd. for $C_7H_{11}N_2^+$: 123.0917; found: 123.0917.

3-Benzyl-1-methyl-1*H*-imidazol-3-ium hexafluorophosphate (BnMIM-H·PF$_6$)

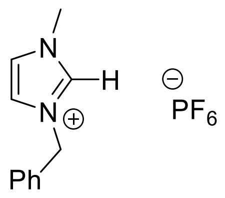

^1H NMR (400 MHz, DMSO) δ 9.23 (s, 1H), 7.79 (t, *J* = 1.7 Hz, 1H), 7.71 (t, *J* = 1.7 Hz, 1H), 7.55 – 7.30 (m, 5H), 3.91 (s, 3H) ppm; ^{13}C NMR (101 MHz, DMSO) δ 137.7, 135.7, 130.0, 129.8, 129.3, 125.0, 123.3, 52.9, 36.8 ppm; ^{19}F NMR (377 MHz, DMSO) δ −68.7 (s), −70.6 (s) ppm; ^{31}P NMR (162 MHz, DMSO) δ −128.8 → −157.0 (m) ppm; HRMS (ESI) (*m/z*) $[M]^+$: exact mass calcd. for $C_{11}H_{13}N_2^+$: 173.1073; found: 173.1073.

2.3 Synthesis of Fluorinated products

General Procedure A

Selectfluor® (**SF®**) (0.564 mmol, 1.0 eq.), protodefluorinated Selectfluor® (**H-TEDA(BF$_4$)$_2$**) (1.128 mmol, 2.0 eq.), starting material (0.846 mmol, 1.5 eq.) and methyl 4-fluorobenzoate (1 mol% based on **SF®**) were added in 2.7 mL dry MeCN in a 5 mL crimp vial equipped with stir bar. The vial was sealed and degassed *via* three cycles of *Freeze-Pump-Thaw*, then filled with N_2. The reaction mixture was stirred under 400 nm LED irradiation for 24 h at rt. After the reaction was completed, diethyl ether was added to the reaction mixture, and instant precipitation of derivatives of **SF®** including **H-TEDA(BF$_4$)$_2$** was observed. The mixture was filtered, and the residue was washed with more diethyl ether followed by evaporation of the solvent under reduced pressure. The crude yield was determined by ^{19}F NMR using

pentafluorobenzene as an internal standard. The residue was purified by column chromatography using silica gel and the specified eluent to afford the desired product.

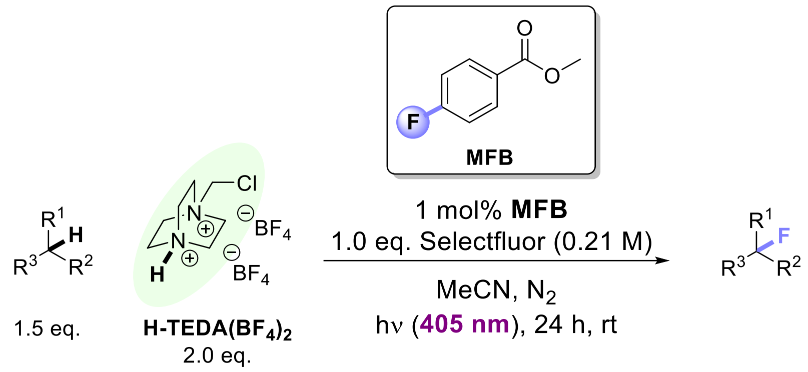

General Procedure B

SF® (0.564 mmol, 1.0 eq.), **H-TEDA(BF$_4$)$_2$** (1.128 mmol, 2.0 eq.), and starting material (0.846 mmol, 1.5 eq.) were added in 2.7 mL dry MeCN in a 5 mL crimp vial equipped with stir bar. The vial was sealed and degassed *via* three cycles of *Freeze-Pump-Thaw*, then filled with N_2. The reaction mixture was stirred under 400 nm LED irradiation for 24 h at rt. After the reaction was completed, diethyl ether was added to the reaction mixture, and instant precipitation of derivatives of **SF®** including **H-TEDA(BF$_4$)$_2$** was observed. The mixture was filtered, and the residue was washed with more diethyl ether followed by evaporation of the solvent under reduced pressure. The crude yield was determined by ^{19}F NMR using pentafluorobenzene as an internal standard. The residue was purified by column chromatography using silica gel and the specified eluent to afford the desired product.

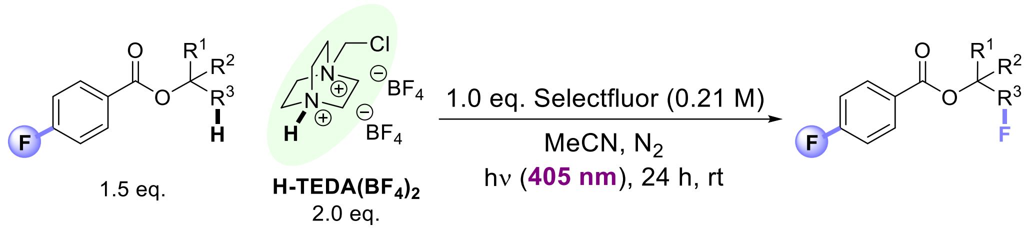

General Procedure C

To a crimp vial equipped with a stir bar were added **SF®** (142 mg, 0.4 mmol, 2.0 eq.), **H-TEDA(BF$_4$)$_2$** (135 mg, 0.4 mmol, 2.0 eq.), glycine (30 mg, 0.4 mmol, 2.0 eq.), and starting material (0.2 mmol, 1.0 eq.). MeCN (1.0 mL) and H_2O (0.9 mL) were then added and stirred for approximately 1 min at rt. A solution of $AgNO_3$ (0.1 mL of a 0.4 M solution in H_2O) was prepared and added to the reaction mixture. The vial was sealed and degassed *via* three cycles of *Freeze-Pump-Thaw*, then filled with N_2. The reaction was heated to 35 °C and stirred for 2 h. Upon completion, the reaction was diluted with EtOAc (1 mL) and transferred to a tube containing 3 mL of saturated $NaHCO_3$. The aqueous phase was extracted with EtOAc (3 x 3 mL) and the combined organic layers were dried over $NaSO_4$, filtered and concentrated under reduced pressure. The crude yield was determined by ^{19}F NMR using pentafluorobenzene as an internal standard. The crude material was purified by column chromatography using silica gel and the specified eluent to yield the desired fluorinated products.

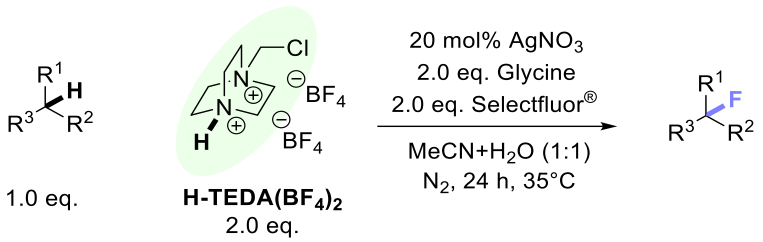

General Procedure D

To a crimp vial equipped with a stir bar were added **SF®** (0.5 mmol, 1.0 eq.), **H-TEDA(BF_4)$_2$** (1.0 mmol, 2.0 eq.), anthraquinone (2 mol%, 2.08 mg) and starting material (0.75 mmol, 1.5 eq.). MeCN (2 mL) was added next, and the vial was sealed and degassed *via* three cycles of *Freeze-Pump-Thaw*, then filled with N_2. The reaction was stirred with 400 nm LED irradiation for 2 h at r.t. The crude yield was determined by ^{19}F NMR using pentafluorobenzene as an internal standard. Upon completion, the reaction mixture was diluted with diethyl ether and instant precipitation of **SF®** derivatives was observed. The mixture was filtered, and the residue was rinsed with more diethyl ether. The solvent was then removed under reduced pressure. The residue was purified by column chromatography using silica gel and the specified eluent to afford the desired fluorinated products.

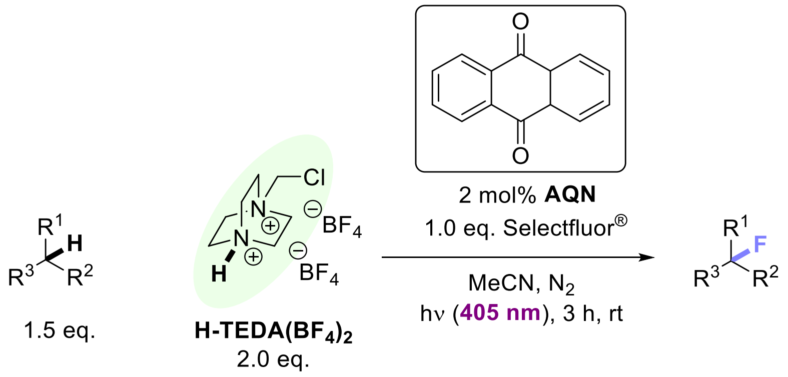

General Procedure E

To a 5 mL clear crimp vial charged with a stir bar were added **SF®** (0.2 mmol, 1.0 eq.), **H-TEDA(BF_4)$_2$** (0.4 mmol, 2.0 eq.), and anhydrous MeCN (2.0 mL). Photocatalyst - acetophenone (1.2 mg, 0.01 mmol), and the starting material (0.3 mmol, 1.5 eq.) were added next. The reaction mixture was degassed *via* three cycles of *Freeze-Pump-Thaw*, then filled with N_2, then irradiated with a 400 nm LED for 2 h at r.t. The crude yield was determined by ^{19}F NMR using pentafluorobenzene as an internal standard. The reaction mixture was then diluted with diethyl ether, filtered, concentrated, and purified by silica gel flash column chromatography using the specified eluent to provide the desired product.

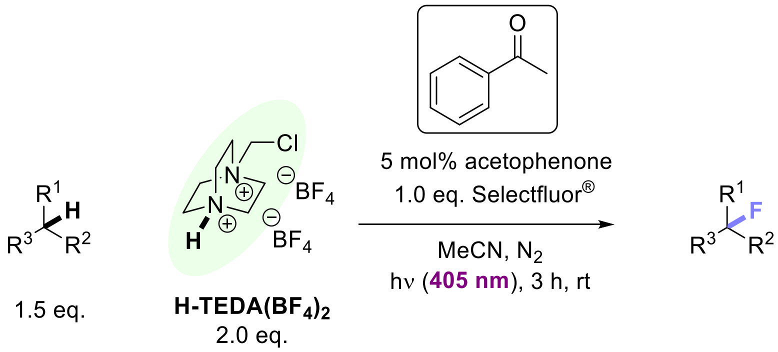

General Procedure F

To a 5 mL clear crimp vial charged with a stir bar were added **SF**® (0.37 mmol, 2.2 eq.), **H-TEDA(BF_4)$_2$** (0.34 mmol, 2.0 eq.), and anhydrous MeCN (2.0 mL). Photocatalyst - 1,2,4,5-tetracyanobenzene (3 mg, 0.017 mmol, 0.1 eq.), and the starting material (0.17 mmol, 1.0 eq.) were added next. The reaction mixture was degassed *via* three cycles of *Freeze-Pump-Thaw*, then filled with N_2, then irradiated using a 365 nm LED for 24 h at r.t. Product identity and yields were determined by ^{19}F NMR spectroscopy with pentafluorobenzene as an internal standard. The reaction mixture was then diluted with diethyl ether, filtered, concentrated, and purified by silica gel flash column chromatography using the specified eluent to provide the desired product.

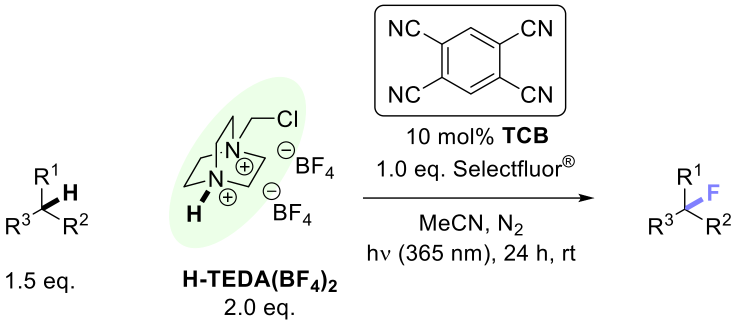

4-Fluoro-4-phenylbutyl 4-fluorobenzoate (2a)

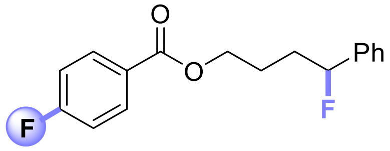

Prepared according to the **General Procedure B**. Yield: 114.6 mg, 70%; colorless viscous liquid; ^{1}H NMR (400 MHz, $CDCl_3$) δ 8.10 – 8.00 (m, 2H), 7.44 – 7.30 (m, 5H), 7.17 – 7.05 (m, 2H), 5.53 (ddd, *J* = 47.5, 8.0, 4.0 Hz, 1H), 4.46 – 4.29 (m, 2H), 2.19 – 1.86 (m, 4H) ppm; ^{19}F NMR (377 MHz, $CDCl_3$) δ −106.2 (tt, *J* = 8.4, 5.5 Hz), −175.8 → −176.3 (m) ppm.

Data are consistent with the literature.[1]

4-Fluoro-1-phenylpentyl 4-fluorobenzoate (2j)

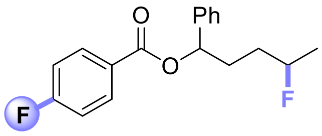

Prepared according to the **General Procedure B**. Yield: 103.0 mg, 60%; white solid; IR (neat) ν (cm^{-1}): 2982, 2937, 1722, 1603, 1506, 1454, 1413, 1387, 1267, 1156, 1111, 1014, 977, 917, 854, 768, 701; ^{1}H NMR (400 MHz, $CDCl_3$) δ 8.33 – 8.19 (m, 2H), 7.65 – 7.40 (m, 5H), 7.33 – 7.20 (m, 2H), 6.16 (dd, *J* = 13.7, 7.0 Hz, 1H), 4.99 – 4.69 (m, 1H), 2.51 – 2.07 (m, 2H), 2.04 – 1.62 (m, 2H), 1.46 (dd, *J* = 23.8, 6.2 Hz, 3H) ppm; ^{13}C NMR (101 MHz, $CDCl_3$) δ 167.1 (s), 164.8 (d, *J* = 1.4 Hz), 164.6 (s), 140.3 (d, *J* = 5.3 Hz), 132.2 (d, *J* = 9.3 Hz), 128.6 (s), 128.2 (d, *J* = 1.4 Hz), 126.6 (dd, *J* = 2.9, 1.5 Hz), 126.4 (s), 115.6 (d, *J* = 22.0 Hz), 90.4 (dd, *J* = 165.6, 18.0 Hz), 76.4 (d, *J* = 29.0 Hz), 32.9 (dd, *J* = 21.1, 13.8 Hz), 32.1 (d, *J* = 4.1 Hz), 21.0 (dd, *J* = 22.7, 2.4 Hz) ppm; ^{19}F NMR (377 MHz, $CDCl_3$) δ −106.0 → −106.1 (m), −173.6 → −174.3 (m) ppm; HRMS (ESI) (*m/z*) [M]$^+$: exact mass calcd. for $C_{18}H_{18}F_2O_2$: 304.1275; found: 304.1269.

4-Fluoro-4-phenylbutyl benzoate (2c)

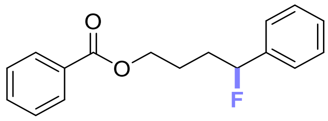

Prepared according to the **General Procedure B**. Yield: 34.0 mg, 63%; colorless viscous liquid; ^{1}H NMR (400 MHz, $CDCl_3$) δ 7.95 (dd, *J* = 5.2, 3.3 Hz, 2H), 7.58 – 7.42 (m, 1H), 7.41 – 7.20 (m, 7H), 5.43 (ddd, *J* = 47.5, 8.1, 4.0 Hz, 1H), 4.41 – 4.15 (m, 2H), 2.15 – 1.70 (m, 4H) ppm; ^{19}F NMR (377 MHz, $CDCl_3$) δ −175.8 → −176.3 (m) ppm.

Data are consistent with the literature.[1]

1-(4-(Fluoromethyl)phenyl)ethan-1-one (2k)

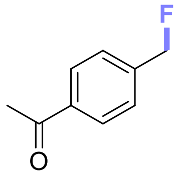

Prepared according to the **General Procedure C**. Yield: 16.4 mg, 54%; colorless liquid; ^{1}H NMR (400 MHz, $CDCl_3$) δ 8.00 (d, *J* = 7.6 Hz, 2H), 7.46 (d, *J* = 7.3 Hz, 2H), 5.46 (d, *J* = 47.2 Hz, 2H), 2.62 (s, 3H) ppm; ^{13}C NMR (151 MHz, $CDCl_3$) δ 197.6 (s), 141.4 (d, *J* = 17.1 Hz), 137.2 (d, *J* = 2.2 Hz), 128.6 (s), 126.8 (d, *J* = 6.6 Hz), 83.6 (d, *J* = 168.7 Hz), 31.9 (s), 30.3 (s), 29.7 (s), 29.6 (s), 29.4 (s), 26.7 (s), 22.7 (s), 14.1 (s), 1.0 (s) ppm; ^{19}F NMR (377 MHz, $CDCl_3$) δ −213.7 (t, *J* = 47.1 Hz) ppm; HRMS (EI) (*m/z*) $[M]^+$: exact mass calcd. for C_9H_9FO: 152.0637; found: 152.0632.

The data are consistent with the literature.[2]

Methyl 4-fluoro-4-methyl-2-(2,2,2-trifluoroacetamido)pentanoate (2d)

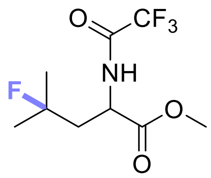

Prepared according to the **General Procedure D**. Yield: 65.0 mg, 50%; colorless liquid; ^{1}H NMR (400 MHz, $CDCl_3$) δ 7.02 (s, 1H), 4.71 (td, *J* = 7.6, 5.2 Hz, 1H), 3.78 (s, 1H), 2.37 – 2.10 (m, 1H), 1.43 (d, *J* = 21.6 Hz, 1H) ppm; ^{13}C NMR (101 MHz, $CDCl_3$) δ 170.8 (s), 156.9 (d, *J* = 37.8 Hz), 115.6 (d, *J* = 287.5 Hz), 95.3 (d, *J* = 165.7 Hz), 53.0 (s), 50.1 (s), 41.4 (d, *J* = 21.0 Hz), 29.7 (s), 27.5 (d, *J* = 24.4 Hz), 26.2 (d, *J* = 24.6 Hz) ppm; ^{19}F NMR (377 MHz, $CDCl_3$) δ -76.6 (s), −138.2 → −138.6 (m) ppm; HRMS (ESI) (*m/z*) $[M+NH_4]^+$: exact mass calcd. for $C_9H_{13}F_4NO_3$: 277.1175; found: 277.1167.

Data are consistent with the literature.[5]

(3*s*,5*s*,7*s*)-1-Fluoroadamantane (2h)

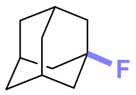

Prepared according to the **General Procedure G**. Yield: 18.0 mg, 58%; white solid; ^{1}H NMR (400 MHz, $CDCl_3$) δ 2.23 (br. s, 1H), 1.90 (dd, *J* = 5.6, 3.0 Hz, 2H), 1.70 – 1.58 (m, 2H) ppm; ^{19}F NMR (377 MHz, $CDCl_3$) δ −128.8 → −129.2 (m) ppm.

Data are consistent with the literature.[1]

Fluorocyclododecane (2i)

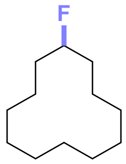

Prepared according to the **General Procedure F**. Yield: 34.0 mg, 91%; white solid; ^{1}H NMR (400 MHz, $CDCl_3$) δ 4.71 (dtt, *J* = 47.5, 7.2, 4.6 Hz, 1H), 1.88 – 1.71 (m, 2H), 1.62 (m, 2H), 1.46 – 1.25 (m, 18H) ppm; ^{13}C NMR (101 MHz, $CDCl_3$) δ 92.5 (d, *J* = 164.8 Hz), 29.9 (d, *J* = 21.0 Hz), 24.1 (s), 23.8 (s), 23.3 (s), 23.2 (s), 20.6 (d, *J* = 6.9 Hz) ppm; ^{19}F NMR (377 MHz, $CDCl_3$) δ −176.1 → −176.9 (m) ppm; HRMS (ESI) (*m/z*) $[M^+\text{-}HF]^+$: exact mass calcd. for $C_{12}H_{22}$: 166.1716; found: 166.1722.

Data are consistent with the literature.[8]

1,10-dibromo-5-fluorodecane (2e)

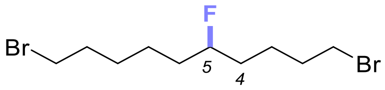

Prepared according to the **General Procedure G** (5F:4F=1.7:1 ratio). Yield: 48.0 mg, 75%; colorless viscous liquid; ^{1}H NMR (400 MHz, $CDCl_3$) δ 4.60 – 4.35 (m, 1H), 3.50 – 3.35 (m, 4H), 1.98 – 1.31 (m, 14H) ppm; ^{19}F NMR (377 MHz, $CDCl_3$) δ −181.2 → −181.6 (m), −181.5 → −181.9 (m) ppm.

Data are consistent with the literature.[1]

4-Fluoropentyl benzoate (2f)

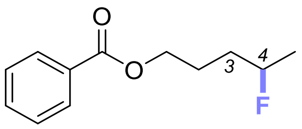

Prepared according to the **General Procedure D** (4F:3F=4:1 ratio). Yield: 36.0 mg, 85%; colorless viscous liquid; ^{1}H NMR (400 MHz, $CDCl_3$) δ 8.04 (d, *J* = 7.7 Hz, 53H), 7.56 (t, *J* = 7.4 Hz, 27H), 7.44 (t, *J* = 7.7 Hz, 55H), 4.87 – 4.44 (m, 30H), 4.43 – 4.28 (m, 54H), 2.19 – 1.50 (m, 121H), 1.36 (dd, *J* = 23.8, 6.2 Hz, 73H), 1.01 (t, *J* = 7.4 Hz, 4H) ppm; ^{19}F NMR (377 MHz, $CDCl_3$) δ −172.1 → −172.7 (m), −183.3 → −183.8 (m) ppm.

Data are consistent with the literature.[1]

4-fluoro-4-phenylbutyl acetate (2b)

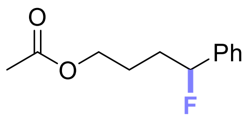

Prepared according to the **General Procedure G**. Yield: 33.6 mg, 80%; colorless viscous liquid; ^{1}H NMR (400 MHz, $CDCl_3$) δ 7.38 – 7.20 (m, 5H), 5.40 (ddd, *J* = 47.7, 8.2, 4.4 Hz, 1H), 4.12 – 3.96 (m, 2H), 1.98 – 1.95 (m, 3H), 1.97 – 1.60 (m, 4H) ppm; ^{19}F NMR (377 MHz, $CDCl_3$) δ −175.9 → −176.4 (m) ppm.

Data are consistent with the literature.[1]

Methyl 2-(4-(1-fluoro-2-methylpropyl)phenyl)propanoate (2l)

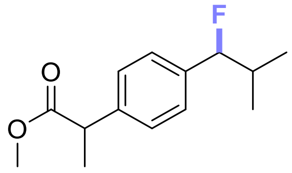

Prepared according to the **General Procedure G**. Yield: 28.6 mg, 60%; colorless viscous liquid; ^{1}H NMR (400 MHz, $CDCl_3$) δ 7.35 – 7.19 (m, 5H), 5.08 (dd, *J* = 47.0, 6.8 Hz, 1H), 3.73 (q, *J* = 7.2 Hz, 1H), 3.66 (s, 3H), 2.21 – 1.94 (m, 1H), 1.50 (d, *J* = 7.2 Hz, 3H), 1.02 (d, *J* = 6.6 Hz, 3H), 0.85 (d, *J* = 6.9 Hz, 3H) ppm; ^{19}F NMR (377 MHz, $CDCl_3$) δ −180.1 (ddd, *J* = 47.0, 17.1, 8.0 Hz) ppm.

Data are consistent with the literature.[1]

Fluorinated hexanone-2 (2g)

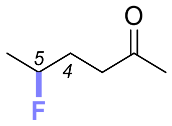

Prepared according to the **General Procedure E** (5F:4F=1.3:1). NMR yield: 92% (according to ^{19}F NMR with pentafluorobenzene as an internal standard); ^{1}H NMR (600 MHz, $CDCl_3$) δ 4.89 (dddd, *J* = 16.1, 12.2, 8.1, 4.6 Hz, 0.4H), 4.72 – 4.58 (m, 0.6H), 2.66 – 2.50 (m, 2H), 2.20 (s, 1.5H), 2.16 (s, 2H), 1.88 – 1.74 (m, 1H), 1.74 – 1.60 (m, 1H), 1.36 – 1.24 (m, 3H) ppm; ^{13}C NMR (151 MHz, $CDCl_3$) δ 208.0 (s), 205.8 (d, *J* = 3.8 Hz), 90.7 (t, *J* = 167.0 Hz), 60.4 (s), 48.4 (d, *J* = 22.9 Hz), 38.9 (d, *J* = 3.6 Hz), 30.9 (d, *J* = 1.1 Hz), 30.7 (d, *J* = 21.0 Hz), 30.0 (s), 28.1 (d, *J* = 21.2 Hz), 21.1 (s), 21.1 (s), 21.0 (s), 9.2 (d, *J* = 5.6 Hz) ppm; ^{19}F NMR (377 MHz, $CDCl_3$) δ -176.1 – -176.8 (m), −182.1 → −182.7 (m) ppm; HRMS (EI) (*m/z*) $[M]^+$: exact mass calcd. for $C_6H_{11}FO$: 118.0794; found: 118.0798.

Data are consistent with the literature.[4]

3 Advanced NMR-Spectroscopic Investigations

3.1 General

All NMR spectroscopic investigations were performed on a Bruker Avance III HD 600 (600.03 MHz) spectrometer with a 5 mm fluorine selective TBIF probe and a Bruker Avance III HD 400 with a 5 mm PABBO BB/19F-1H/D Z-GRD probe. NMR data were processed with TopSpin 4.1.1 and evaluated and plotted with TopSpin 3.2 and Topspin 4.1.1 software. ^{1}H spectra without TMS as internal standard were calibrated on the solvent residual peak of CD_3CN at $\delta(^{1}H)$ = 1.94 ppm or in the CD_3CN / D_2O (1:1) mixture on the CD_3CN signal at $\delta(^{1}H)$ = 2.50 ppm; otherwise, spectra were referenced to TMS (tetramethylsilane) at $\delta(^{1}H)$ = 0.00 ppm. Further calculations, plotting and preparation of the obtained data was performed with Origin 2022, Microsoft Excel 2019 and Corel Draw 2020 software. For *in situ* illumination NMR reaction monitoring the reported setup with 5 mm thin wall NMR tubes was used.[9]

3.2 Chemicals

All commercially available chemicals were purchased from major supplier Sigma Aldrich and were used without further purification. CD_3CN was distilled over CaH_2 and degassed using the freeze-pump-thaw method. After degassing, CD_3CN was stored in a flask with 3 Å molecular sieves under inert gas. TMS was degassed using the *Freeze-Pump-Thaw* method and stored inside the glove box.

3.3 General method for quantitative *in situ* illumination NMR reaction monitoring

The reaction yield/time of the NMR reactions are not directly comparable to the stirred reactions in batch due to the following limitations: i) the light intensity transmitted from the LED to the reaction is low (due to losses from using an optical fiber to transmit light inside the NMR spectrometer), ii) the lack of stirring (an unavoidable disadvantage of *in situ* NMR kinetics) and iii) **SF®** not being fully dissolved under these conditions. Reactions in NMR tubes do not reach as high yields/conversion rates as the batch reactions. Nevertheless, this approach was considered sufficient to interpret relative trends in reactions.

For *in situ* illumination of the NMR tube inside the NMR spectrometer an illumination setup as described in literature was used in combination with a Nichia NVSU233B(T) LED emitting at a peak wavelength of 405 nm.[9] The LED was run with forward current of 1.4 A. The kinetic studies were performed at 308 K (35 °C) or at rt (25 °C). The starting point of each kinetic (t = 0 s) was measured before starting the illumination and during illumination, ^{1}H and ^{19}F spectra were recorded alternately. The following ^{1}H and ^{19}F NMR experiments were measured to investigate the quantitative evaluation of the reaction. Due to the high concentration of the NMR samples a S/N of > 250:1 was already achieved in single scan experiments. Additional S/N enhancement was achieved by reaction monitoring with an NS (number of scans) of 4 for ^{1}H and ^{19}F experiments and heteronuclear $^{1}H - {}^{19}F$ coupling was suppressed by using a zgig pulse program (inverse gated decoupled). A relaxation delay d_1 = 6-8 s was applied to ensure full relaxation of all signals and pulse lengths for ^{1}H and ^{19}F experiments were calibrated for every experiment.

3.4 Reaction monitoring of fluorination reactions with Selectfluor® and protonated Selectfluor®

3.4.1 NMR sample preparation

The entire NMR sample preparation for each experiment was performed under inert conditions. The actual preparation for the reaction solutions were carried out inside the glove box under Argon (M. Braun

Inertgas-Systeme GmbH). The glass fiber for *in situ* illumination and the NMR tube with the reaction solution were merged by Schlenk line technique. In this process, the glass fiber (Thorlabs, fiber type: MM, FP1500URT, 0.50 NA, 300-1200 nm, 1500 µm core) was inserted into the NMR tube under inert gas and sealed with parafilm to prevent oxygen migration inside the NMR tube. In order to ensure identical reaction conditions for the reaction profiles, each reaction solution was prepared out of stock solutions. Therefore, the stock solutions of the substrates were combined in the NMR tube inside the glove box. Afterwards the reaction solution was shaken intensively to ensure a homogeneous sample.

3.4.2 Reaction monitoring of C(sp^3)-H fluorination reactions with an unprotected amino acid precursor, $AgNO_3$, Selectfluor® and **H-TEDA(BF_4)$_2$**

For a first insight into the reaction kinetics of the radical C-H fluorination reactions using unprotected amino acids as precursors, $AgNO_3$ as a silver(I) catalyst and Selectfluor® as fluorination reagent, a model reaction with and without **H-TEDA(BF_4)$_2$** loading was investigated (Scheme S1).

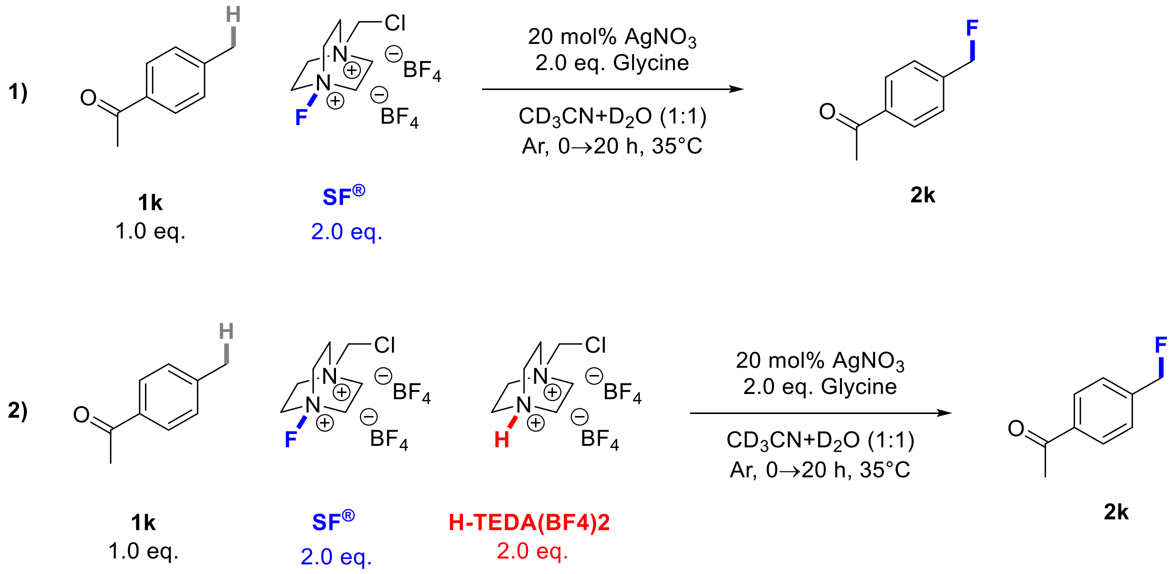

Scheme S1: Reaction conditions for reaction monitoring by NMR spectroscopy at 35 °C without addition of **H-TEDA(BF_4)$_2$** (1) and with 2.0 eq. **H-TEDA(BF_4)$_2$** loading (2).

The entire NMR sample preparation for both experiments were performed under inert gas conditions. The actual preparation for the reaction solutions were carried out inside the glove box. For monitoring the influence of **H-TEDA(BF_4)$_2$** in the model reaction, five stock solutions were prepared (see Table S14) in separate flasks. All stock solutions were degassed after dissolving the compounds in a solvent mixture of CD_3CN and D_2O (1:1) and stored inside the glove box. In reaction **1** without addition of **H-TEDA(BF_4)$_2$**, 0.1 ml of each stock solution was transferred into the NMR tube and additional 0.1 ml of the 1:1 solvent mixture was added (see Table S14). In reaction **2** with additional **H-TEDA(BF_4)$_2$**, 0.1 ml of each stock solution was transferred into the NMR tube.

Table S14: Calculated amounts of the compounds for the preparation of the stock solutions for the model reaction 1 and 2 (see Scheme S1).

compound	equivalents	concentration [mM]	amount of substrate [µmol][a]	mass [mg][a]
**1k**	1.0	50	420	12.75 (12.69 µl)
**SF®**	2.0	100	840	297.6
**H-TEDA(BF_4)$_2$**	2.0	100	840	282.5
$AgNO_3$	20 mol%	10	84	14.3
glycine	2.0	100	840	14.3

[a]The amounts are based on 700 µL stock solutions dissolved in D_2O/CD_3CN (1:1).

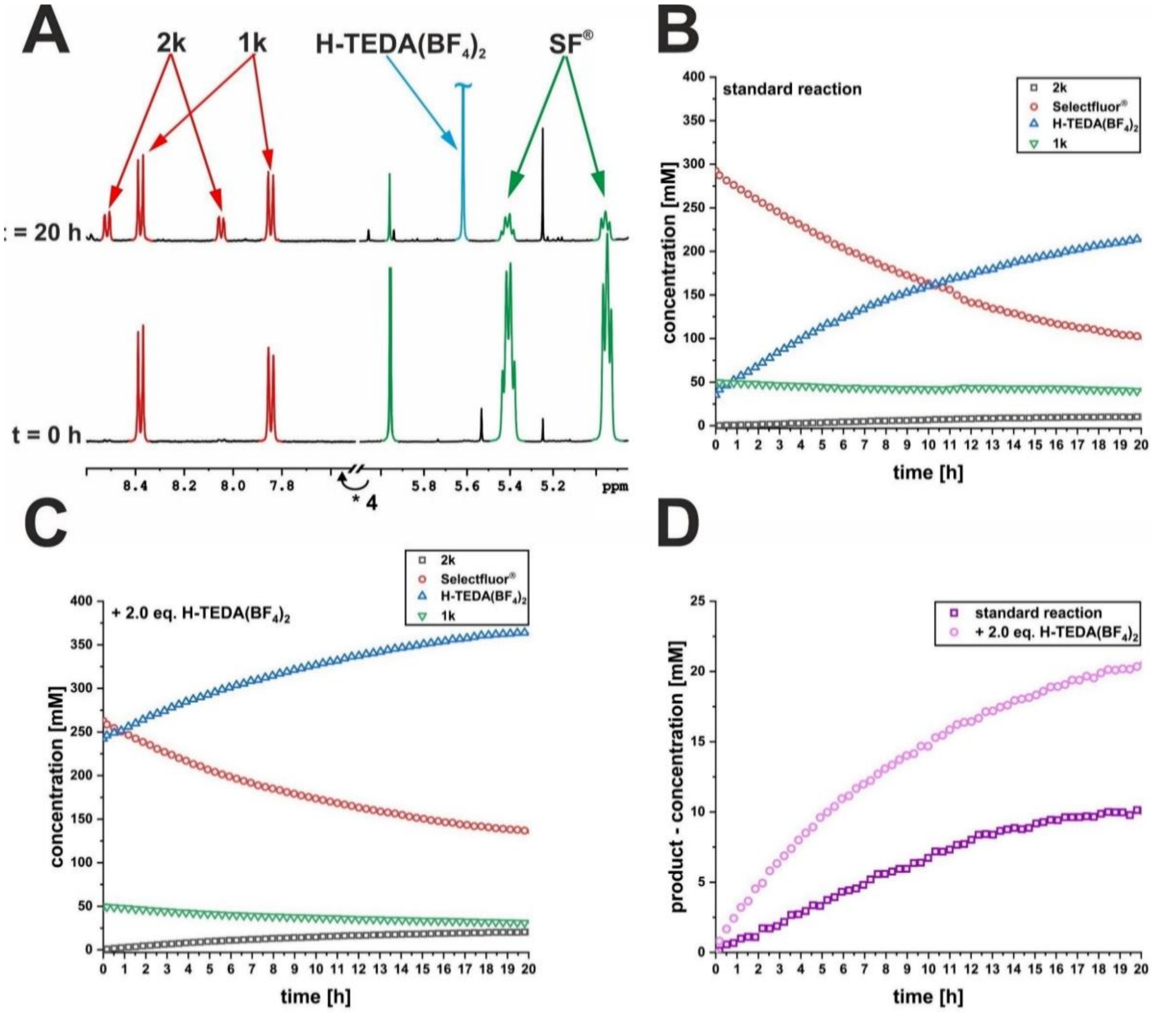

E

**standard reaction**		**+ 2.0 eq. H-TEDA(BF_4)$_2$**	
equation	y = A + B*x	equation	y = A + B*x
B	2.08408 ± 0.04366	B	0.7043 ± 0.0117
A	0	A	0
R^2 (cor.)	0.97268	R^2 (cor.)	0.98541

Figure S5: **A:** ^{1}H spectra of reaction 1 (Scheme S1) before illumination (t = 0 h) and after illumination for 20 h. The integrated and plotted signal regions for each compound is highlighted. **B:** Kinetic profile of reaction **1** (see Scheme S1, standard reaction) without **H-TEDA(BF_4)$_2$** loading. **C:** Kinetic profile of reaction **2** with 2.0 eq. **H-TEDA(BF_4)$_2$** loading. **D:** Comparison of the product concentration curves for reaction **1** (no **H-TEDA(BF_4)$_2$** loading) and reaction **2** (with 2.0 eq. **H-TEDA(BF_4)$_2$** loading). In reaction **1**, 10 mM product is formed while in reaction **2** appr. 20 mM product is generated after 20 h. Product yield is increased by a factor of 2 with 2.0 eq. of **H-TEDA(BF_4)$_2$** loading. **E:** Calculated initial rates for the standard reaction (left) and with 2.0 eq. **H-TEDA(BF_4)$_2$** (right).

3.4.3 Reaction monitoring of photocatalytic C(sp^3)-H fluorination with acetophenone, SF® and H-TEDA(BF_4)$_2$

The NMR samples for reaction monitoring were prepared under inert gas conditions of photosensitization auxiliary method as described in 3.4.1. For monitoring the influence of **H-TEDA(BF_4)$_2$**, stock solutions of **H-TEDA(BF_4)$_2$** and acetophenone (5 mol%) were prepared. Further components (substrate, **SF®**) were also prepared with stock solutions. The weights of the components for the stock solutions are given in Table S15.

Table S15: Calculated amounts of the compounds for reaction monitoring of photocatalytic C(sp^3)-H fluorination and **H-TEDA(BF_4)$_2$.**

compound	equivalents	concentration [mM]	amount of substrate [µmol][a]	mass [mg][a]
**1g**	1.5	150	480	41.3 (50.98 µl)
**SF®**	1.0	100	320	113.4
**H-TEDA(BF_4)$_2$**	2.0	200	640	215.2
acetophenone	5 mol%	5.0	16	1.92 (2.37 µl)

[a] The amounts are based on 800 µL stock solutions dissolved in dry and degassed CD_3CN.

The corresponding amounts of compounds (see Table S15) were transferred into flasks and then diluted with 800 µL anhydrous and degassed CD_3CN. Again, the stock solutions were degassed using the freeze-pump-thaw method and stored inside the glove box.

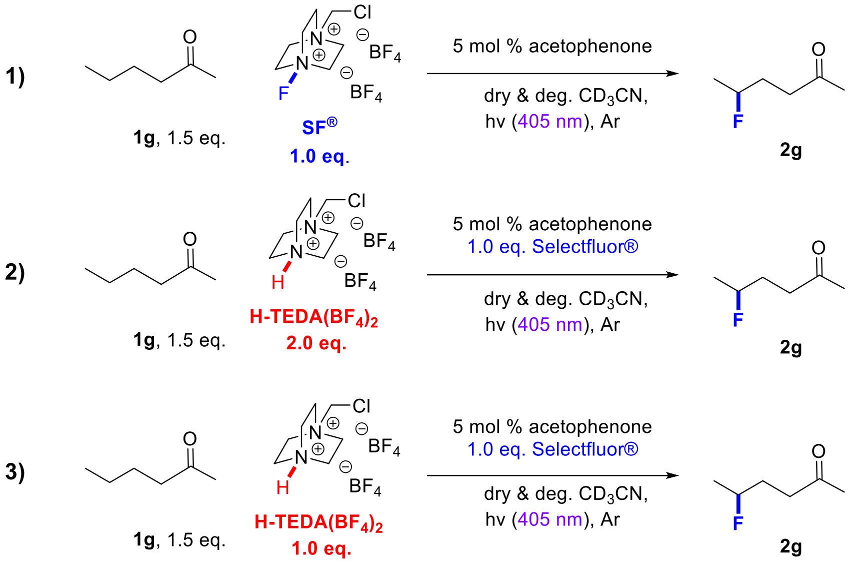

Scheme S2: Model reaction for monitoring of photocatalytic C(sp^3)-H fluorination by *in situ* illumination NMR spectroscopy. Reaction **1)** without **H-TEDA(BF$_4$)$_2$**; Reaction **2)** with 2.0 eq. **H-TEDA(BF$_4$)$_2$** loading; Reaction **3)** with 1.0 eq. **H-TEDA(BF$_4$)$_2$** loading.

Depending on the reaction conditions (see Scheme S2), different amounts of **H-TEDA(BF$_4$)$_2$** were added to the reaction solution. In reaction **1**, 75 µL of each stock solution of **1g**, **SF®** and acetophenone were transferred into the NMR tube. In addition, 75 µL of CD_3CN (dry and degassed) was added to the reaction solution. In reaction **2**, 75 µL of each stock solution of **1g**, **SF®**, acetophenone and **H-TEDA(BF$_4$)$_2$** (2.0 eq.) were transferred into the NMR tube. No further solvent was added to the reaction solution. In reaction **3**, 75 µL of each stock solution of **1g**, **SF®** and acetophenone were transferred into the NMR tube. In addition, 37.5 µL of stock solution of **H-TEDA(BF$_4$)$_2$** (1.0 eq.) and 37.5 µL of CD_3CN (dry and degassed) were added to the reaction solution. The samples were shaken intensively to ensure a homogeneous reaction solution. Lastly, the *in situ* illumination insert was combined with the NMR tube as described in 3.4.1. Each reaction solution was illuminated at a wavelength range of 405 nm under the same reaction conditions.

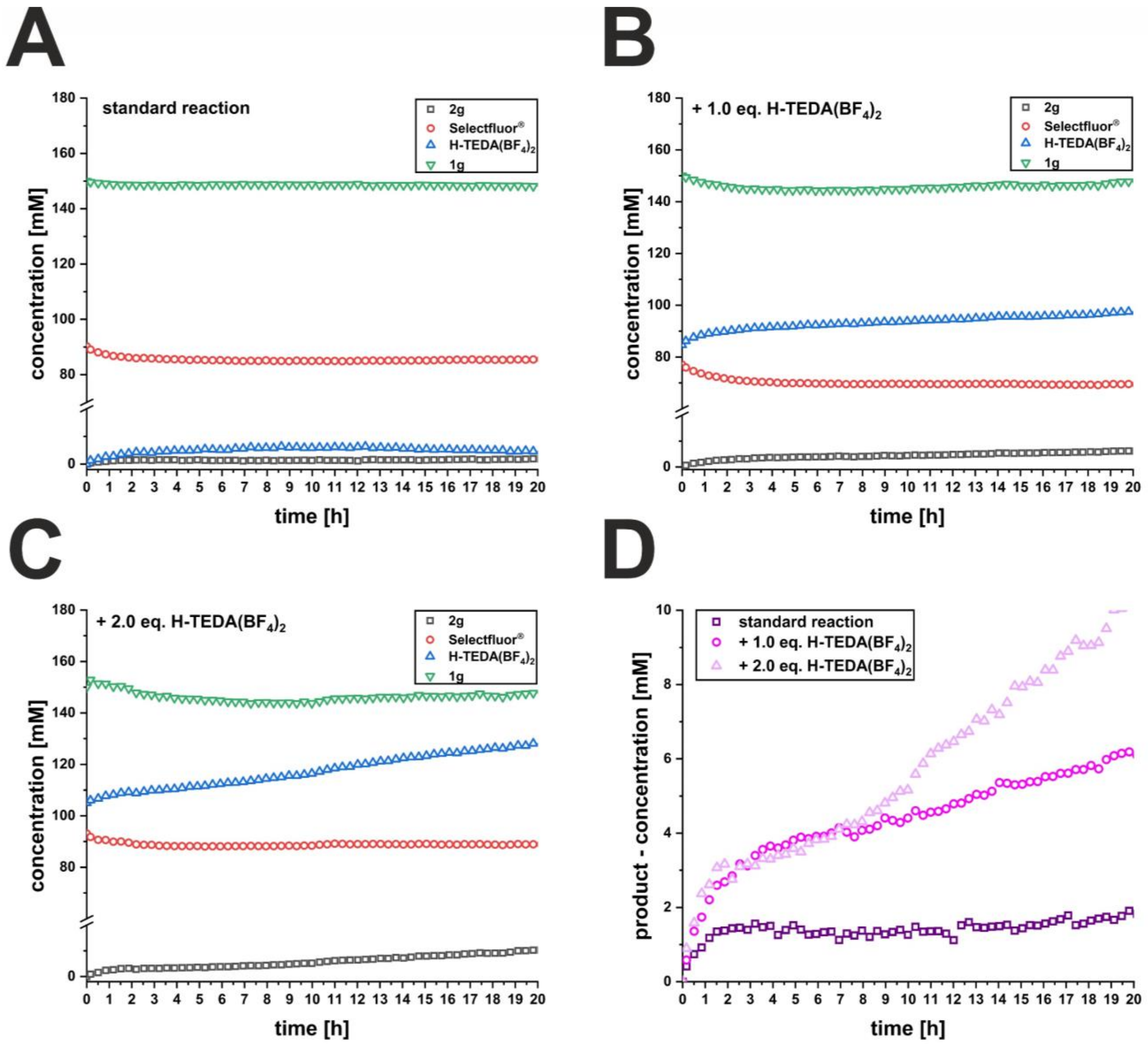

Figure S6: **A-C**: Kinetic profiles of the photosensitization auxiliary reaction with different loadings of **H-TEDA(BF$_4$)$_2$**. **D**: Comparison of the reaction profiles of product formation with different equivalents of **H-TEDA(BF$_4$)$_2$**. While the reaction without **H-TEDA(BF$_4$)$_2$** loading gave only 1mM of product formation after 20 h of *in situ* illumination, product concentrations of up to 6 mM or 10 mM could be obtained by the addition of 1.0 eq. or 2.0 eq. **H-TEDA(BF$_4$)$_2$**.

Plotting the absolute values of the product concentration after 7 h *in situ* illumination of the photocatalytic C(sp^3)-H fluorination reactions **1, 2** and **3** (see Scheme S2) without, with 1.0 eq. and with 2.0 eq. **H-TEDA(BF$_4$)$_2$** loading indicates increasing product formation rates by increasing the amount of **H-TEDA(BF$_4$)$_2$**. While the standard reaction (no **H-TEDA(BF$_4$)$_2$** loading) shows product formation at a rate of 1.39E-5 mM/s, the product formation rate was increased to 3.97E-5 mM/s by addition of 1.0 eq. of **H-TEDA(BF$_4$)$_2$**. An even higher product formation rate (12.3 E-5 mM/s) was obtained by addition of 2.0 eq. of **H-TEDA(BF$_4$)$_2$**. Overall, increased **H-TEDA(BF$_4$)$_2$** loading has a positive effect on the product formation rate of the photocatalytic fluorination reaction. Furthermore, in contrast to the standard reaction where only 1 mM of product is generated after 20 h of *in situ* illumination, 1.0 eq. and 2.0 eq. of **H-TEDA(BF$_4$)$_2$** loading increased the product formation up to 6 mM and 10 mM respectively. In summary, the kinetics of the photocatalytic C(sp^3)-H fluorination reaction indicated that **H-TEDA(BF$_4$)$_2$** loading improves the reaction rate and product formation.

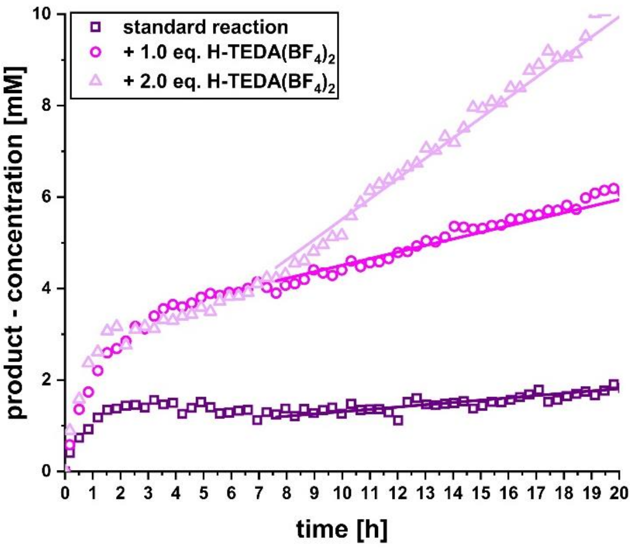

equivalents of H-TEDA(BF$_4$)$_2$ added	slope (yEr±) [mM/s]	R^2 (cor.)
0	1.39e-5 ± 0.0037	0.801
1.0	3.97e-5 ± 0.0054	0.937
2.0	12.3e-5 ± 0.0086	0.982

Figure S7: Reaction kinetics (left) and product formation rates (right) for the photocatalytic C(sp^3)-H fluorination reaction with different **H-TEDA(BF$_4$)$_2$** loadings.

3.4.4 Reaction monitoring of photochemical C(sp^3)-H fluorination with **H-TEDA(BF$_4$)$_2$**

The entire NMR sample preparation for monitoring of photochemical C(sp^3)-H fluorination reaction was executed under inert gas conditions. The actual preparation of the reaction solution was done inside the glove box. The insert of the glass fiber into the NMR tube for *in situ* illumination was done as described in 3.4.1. The weights of the components for each substrate are given in Table S16.

Table S16: Calculated amounts of the compounds for the reaction monitoring of photochemical C(sp^3)-H fluorination reaction.

compound [a]	equivalents	concentration [mM]	amount of substrate [µmol][a]	mass [mg][a]
**1b**	1.5	75.0	75.0	14.42 (14.39 µl)
**SF®**	1.0	50.0	50.0	17.71
**H-TEDA(BF$_4$)$_2$**	2.0	100.0	100.0	33.63

[a] The amounts are dissolved in 1.0 ml dry and degassed CD_3CN.

The corresponding amounts of compounds (see Table S16) were transferred into flasks and then diluted with 1.0 ml anhydrous and degassed CD_3CN.

1) 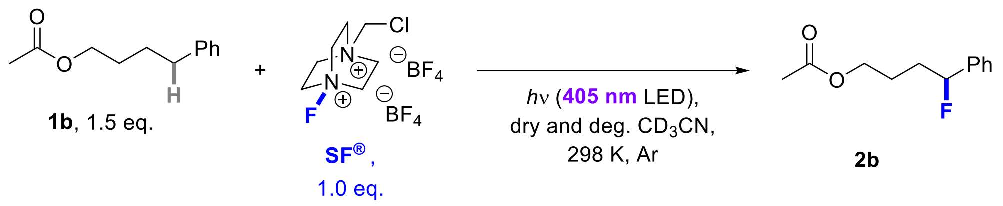

2) 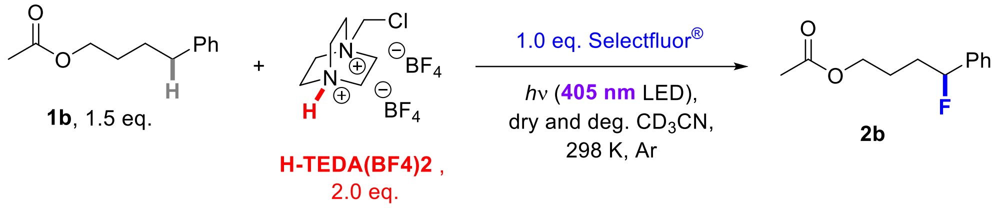

Scheme S3: Model reaction for monitoring of photochemical C(sp^3)-H fluorination by *in situ* illumination monitoring. Reaction **1)** under standard conditions and reaction **2)** with 2.0 eq. **H-TEDA(BF$_4$)$_2$** loading.

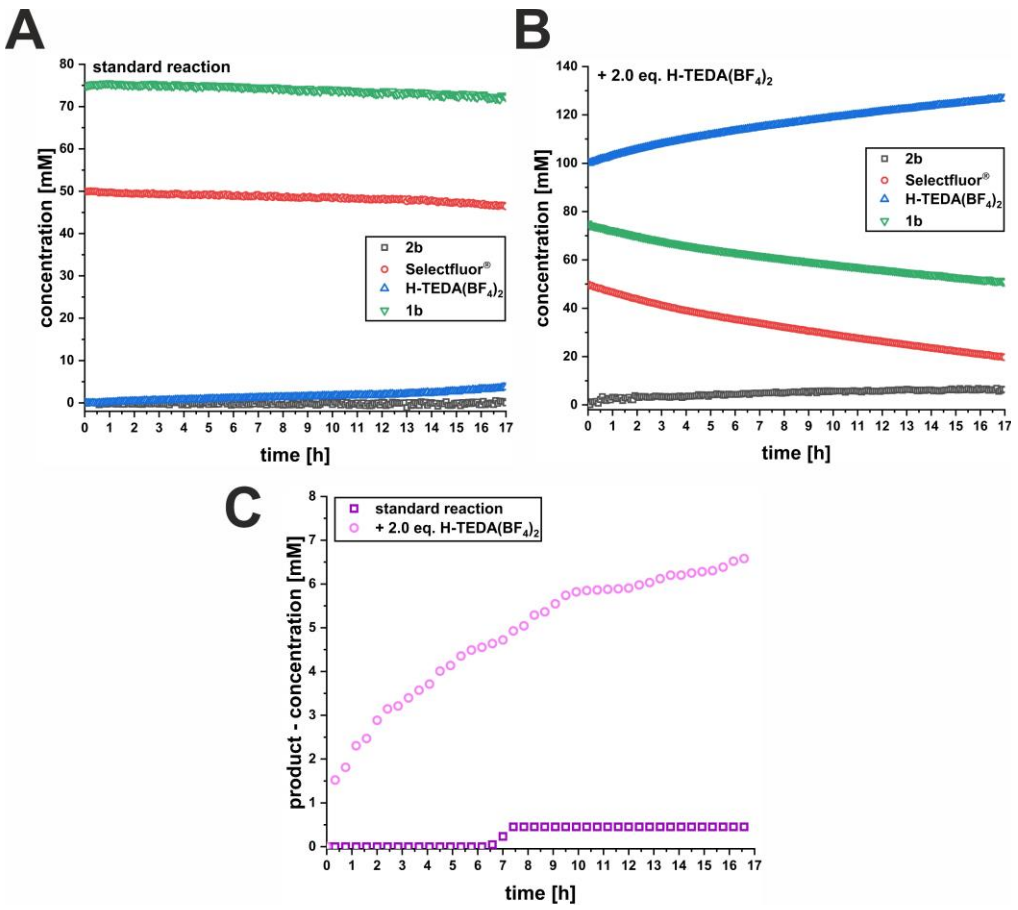

Figure S8: **A and B**: Kinetic profiles of the photochemical fluorination reaction under standard conditions (**A**, no **H-TEDA(BF$_4$)$_2$** loading) and with 2.0 eq. **H-TEDA(BF$_4$)$_2$** loading (**B**). **C**: Comparison of the reaction profiles of product formation with different amounts of **H-TEDA(BF$_4$)$_2$** loading.

In reaction **1** and **2**, 0.3 mL of the solutions were transferred into the NMR tube under inert conditions. Lastly, the *in situ* illumination insert was combined with the NMR tube as described in 3.4.1. Each reaction solution was illuminated at a wavelength range of 405 nm under identical conditions at 298 K. Scarcely

any product is formed under standard conditions. When repeating the standard reaction with 2.0 eq. **H-TEDA(BF_4)$_2$** loading, the product formation starts at the beginning of *in situ* illumination. In comparison, 0.12 mM of product was formed in the standard reaction whereas 6.6 mM of product was generated by addition of 2.0 eq. **H-TEDA(BF_4)$_2$** after 17 h of *in situ* illumination.

4 Diffusion ordered spectroscopy (DOSY)

4.1 Diffusion ordered spectroscopy (DOSY) measurements for aggregate-investigations in C(sp^3)-H fluorination reactions with **SF®** and **H-TEDA(BF_4)$_2$** loading

4.1.1 Introduction

^{1}H-NMR spectra of *in situ* illumination-NMR experiments of photochemical C(sp^3)-H fluorination of substrate **1a** (without **H-TEDA(BF_4)$_2$**) indicated a strong downfield shift of the **H-TEDA(BF_4)$_2$** signals during the reaction (see yellow box in Figure S9i)-**A**). Contrary, no downfield shift of **H-TEDA(BF_4)$_2$** signals were observed in the ^{1}H-NMR spectra (yellow box in Figure S9i)-**B**) for fluorination of **1a** with 1.0 eq. **H-TEDA(BF_4)$_2$** loading**.** Aggregation of **H-TEDA(BF_4)$_2$** leads to enhanced intermolecular interactions with Selectfluor® resulting in faster product formation (see Figure S9ii)-**B**) and higher reaction rates. The end of the induction period was accompanied with a decrease in the pH of the reaction (Figure S9iii), converging on a value of pH = 2.3, as expected from the formation of nascent **H-TEDA(BF_4)$_2$**.

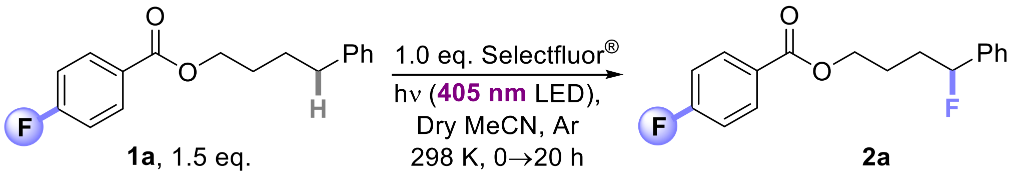

i)

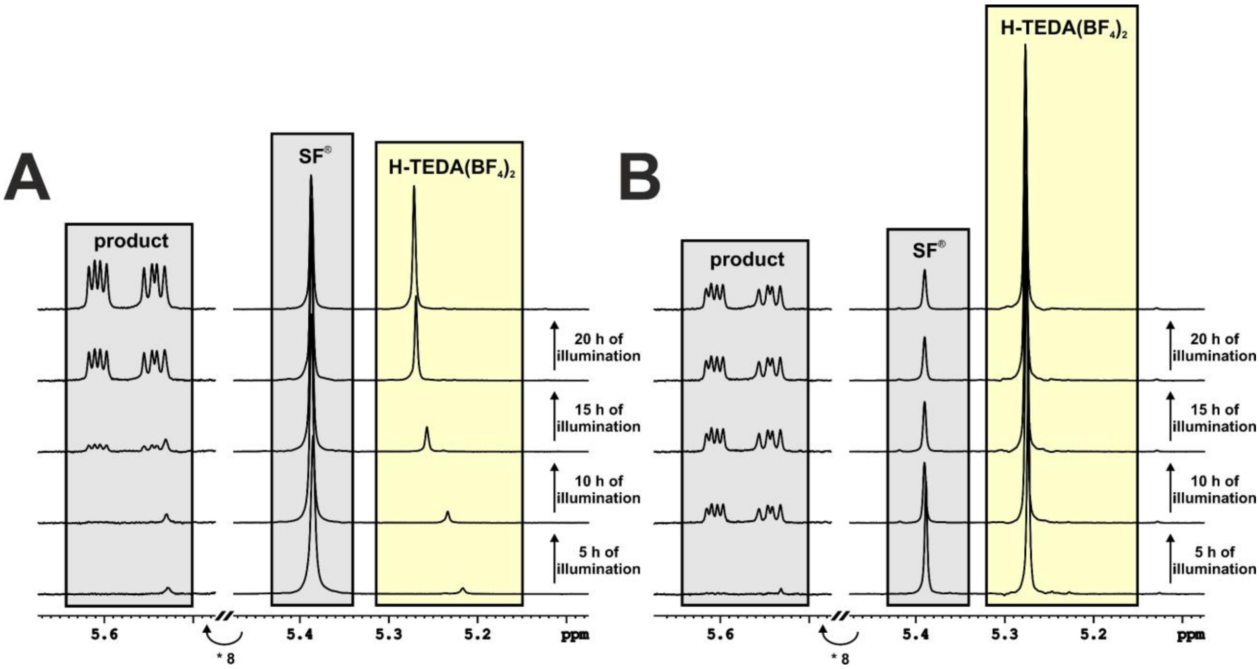

ii)

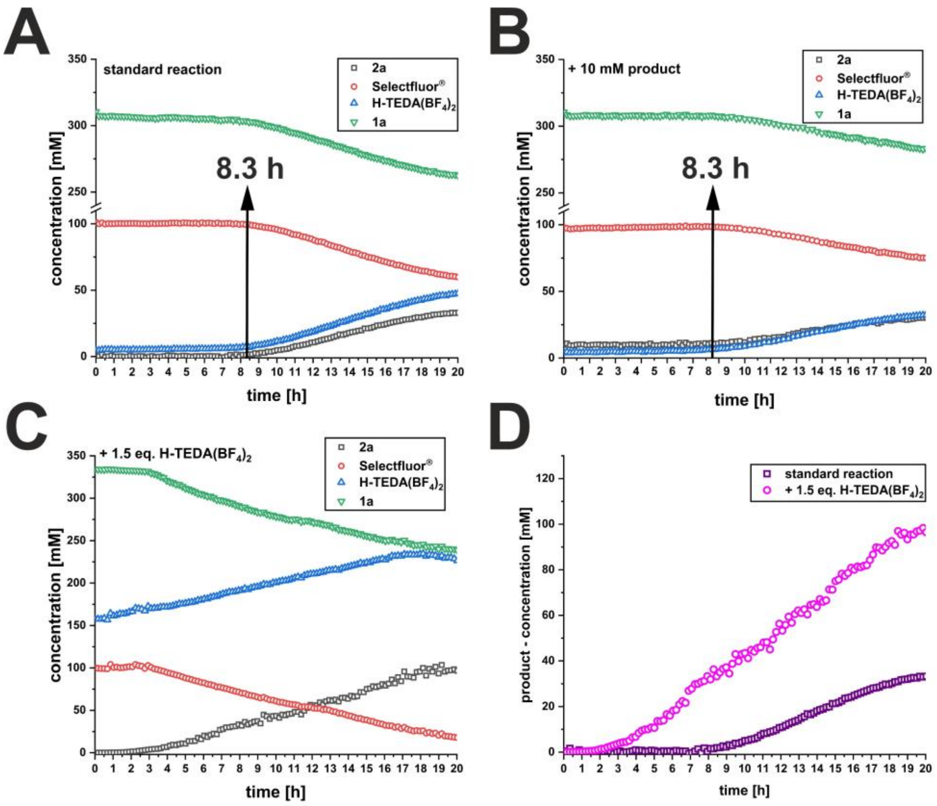

iii)

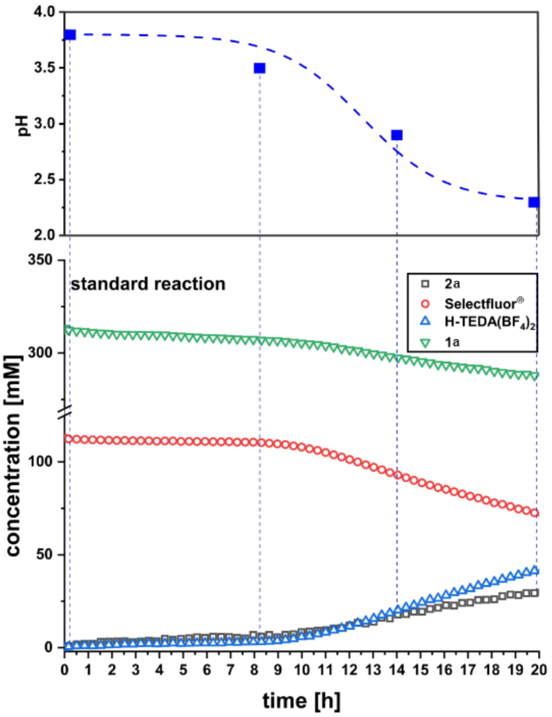

Figure S9: A: i) Stacking of ^{1}H-NMR spectra of photochemical C(sp^{3})-H fluorination of substrate **1a** (without **H-TEDA(BF$_4$)$_2$**). The spectra were recorded periodically after 5 h of *in situ* illumination. A downfield shift of the **H-TEDA(BF$_4$)$_2$** signals is observed in the ^{1}H-NMR spectra during monitoring fluorination of **1a** (yellow box). **B:** Stacking of ^{1}H-NMR spectra of photochemical C(sp^{3})-H fluorination of **1a** with 1.0 eq. of **H-TEDA(BF$_4$)$_2$.** The ^{1}H-NMR spectra were recorded periodically after 5 h of *in situ* illumination. No downfield shift of the **H-TEDA(BF$_4$)$_2$** signals was observed during reaction monitoring. **ii)**

^1H *in situ* illumination NMR reaction monitoring of the **H−TEDA(BF$_4$)$_2$** promotionary effect on the photosensitized auxiliary fluorination of **1a**. (A) Kinetic profiles of the photochemical reaction under standard conditions, (B) with 10 mM product at the start, (C) with 1.5 eq. **H−TEDA(BF$_4$)$_2$** at the start. **D**) Detailed comparison of the product formation profiles without and with 1.5 eq. of **H−TEDA(BF$_4$)$_2$**. **iii)** Change of pH during the reaction.

4.1.2 General

^1H-diffusion measurements were performed with the convection suppressing 2D double-stimulated echo experiment DSTE (dstebpgp3s) developed by Müller and Jerschow in a pseudo 2D mode.[10] An effective diffusion time of 50 ms was applied. A set of 16 dummy scans/32 scans, respectively, were used with relaxation delays of 3-4 s. SMSQ10.100 as gradient program and a linear ramp with 32 increments between 5% and 95% of the maximum gradient strength were applied. For z-only gradients −100%, −13.17%, −17.13% and −15.17% were used.

DOSY kinetic measurements were performed with the convection suppressing 1D double-stimulated echo experiment DSTE using bipolar gradients (dstebpgp3s1d) developed by Müller and Jerschow. [10] An effective diffusion time of 50 ms was applied. A set of 4 dummy scans/16 scans, respectively, were used with relaxation delays of 4 s. SMSQ10.100 as gradient program and a linear ramp with 32 increments between 5% and 95% of the maximum gradient strength were applied. For z-only gradients 5−95 %, −13.17%, −17.13% and −15.17% were used. The gradient pulses were adjusted for each compound in order to obtain optimized diffusion times. NMR spectra were processed with TopSpin 4.1.1 and the diffusion coefficients and average volume data measured with 1D and 2D double-stimulated echo pulse programs were determined according to Müller and Jerschow.[10] The gradient pulses were adjusted for each compound in order to obtain optimized diffusion times. For the size estimation with DOSY, the experimental translational self-diffusion coefficients D of the molecules in solution were determined according to the Stejskal-Tanner equation.[11] The correction factor *c* of the Stokes-Einstein equation was calculated according to the semi-empirical modification by Chen using literature known values for the solvent (r_{CD_3CN} = 2.86 Å).[12,13]

4.1.3 NMR sample preparation

The entire NMR sample preparation for 1D and 2D DSTE DOSY experiments was executed similar as for reaction monitoring in 3.4.2, 3.4.3 and 3.4.4. The entire NMR sample preparation for each experiment was performed under inert gas conditions and prepared inside the glove box. Anhydrous and degassed TMS was stored inside the fridge of the glove box and added inside of the glove box. The glass fiber in case for simultaneous *in situ* illumination and DOSY reaction monitoring was added into the NMR tube by Schlenk line technique as described in 3.4.1. Each NMR sample was fastened with parafilm to prevent oxygen and moisture during the measurement.

4.1.4 DOSY studies of **SF®** and **H-TEDA(BF$_4$)$_2$** using pure compounds at different concentrations and reaction mixtures with different **H-TEDA(BF$_4$)$_2$** loadings

In order to prove whether pure **H-TEDA(BF$_4$)$_2$** aggregates, mean diffusion coefficients for **H-TEDA(BF$_4$)$_2$** at different concentrations were determined. For the investigation of **H-TEDA(BF$_4$)$_2$**, a stock solution for the NMR probes with concentrations of 90 mM, 50 mM, 20 mM and 1 mM was prepared.

To compare the aggregation of **H-TEDA(BF$_4$)$_2$** under synthetic reaction conditions, a further probe was prepared at a concentration of 209 mM. Due to moderate solubility of **H-TEDA(BF$_4$)$_2$** at higher concentrations of 90 mM, the NMR probe was prepared separately. **H-TEDA(BF$_4$)$_2$** was not fully dissolved under these concentrations (c = 209 mM), meaning the concentration in solution is lower and therefore the same volume of **H-TEDA(BF$_4$)$_2$** is not reached as under synthetic reaction conditions.

Table S17 Volumes of **SF®** and **H-TEDA(BF$_4$)$_2$**, pure and with different **H-TEDA(BF$_4$)$_2$** loading in anhydrous and degassed CD_3CN at 308 K, measured by DOSY NMR experiments (details see ESI chapter 2.1.4).

**Entry**	**Compound(s)**	**C [mM]**	**Mean diffusion coefficient with SD [E-9 m^2/s]**	**Average volume with SD [Å^3]**
**1**	**H-TEDA(BF$_4$)$_2$** (precipitation)	209	1.43 ± 0.00928	973.8 ± 16.47
**2**	**H-TEDA(BF$_4$)$_2$**	90	1.47 ± 0.00426	934.9 ± 7.08
**3**	**H-TEDA(BF$_4$)$_2$**	50	1.56 ± 0.00817	878.1 ± 11.90
**4**	**H-TEDA(BF$_4$)$_2$**	20	1.68 ± 0.0200	796.7 ± 24.08
**5**	**H-TEDA(BF$_4$)$_2$**	1.0	2.04 ± 0.0388	506.2 ± 22.93
**6**	**SF®**	90	1.49 ± 0.00971	718.0 ± 11.80
**7**	**SF®** / **H-TEDA(BF$_4$)$_2$**	90 / 90	**SF®**: 1.35 ± 0.00452<br>**H-TEDA(BF$_4$)$_2$**: 1.35 ± 0.0144	**SF®**: 1054.9 ± 9.13<br>**H-TEDA(BF$_4$)$_2$**: 1111.1 ± 30.87
**8**	**SF®** / **H-TEDA(BF$_4$)$_2$**	40 / 40	**SF®**: 1.54 ± 0.0359<br>**H-TEDA(BF$_4$)$_2$**: 1.54 ± 0.0295	**SF®**: 1045.2 ± 61.8<br>**H-TEDA(BF$_4$)$_2$**: 1040.1 ± 50.8
**9**	**SF®** / **H-TEDA(BF$_4$)$_2$**	40 / 30	**SF®**: 1.62 ± 0.0246<br>**H-TEDA(BF$_4$)$_2$**: 1.64 ± 0.0171	**SF®**: 812.9 ± 31.2<br>**H-TEDA(BF$_4$)$_2$**: 791.8 ± 21.0
**10**	**SF®** / **H-TEDA(BF$_4$)$_2$**	40 / 20	**SF®**: 1.60 ± 0.0176<br>**H-TEDA(BF$_4$)$_2$**: 1.65 ± 0.0197	**SF®**: 762.7 ± 21.3<br>**H-TEDA(BF$_4$)$_2$**: 709.6 ± 21.2
**11**	**SF®** / **H-TEDA(BF$_4$)$_2$**	40 / 10	**SF®**: 1.65 ± 0.0187<br>**H-TEDA(BF$_4$)$_2$**: 1.71 ± 0.0021	**SF®**: 765.5 ± 21.9<br>**H-TEDA(BF$_4$)$_2$**: 695.6 ± 21.5
**12**	BF_4^- of **SF®**	90	1.78 ± 0.0114	461.1 ± 3.41
**13**	BF_4^- of **H-TEDA(BF$_4$)$_2$**	90	1.80 ± 0.0245	503.9 ± 7.79
**14**	BF_4^- of **SF®** / **H-TEDA(BF$_4$)$_2$** complex	90	1.68 ± 0.0348	620.2 ± 22.42

Table S18: Mean diffusion coefficients and calculated average volumes of **SF®**, **H-TEDA(BF$_4$)$_2$** as pure compounds and in mixture at different concentrations in anhydrous and degassed CD_3CN at 308 K.

entry	compound	concentration [mM]	mean diffusion coefficient [E-9 m^2/s] with SD	average volume with SD [Å^3]
**1**	**H-TEDA(BF$_4$)$_2$**	208.9 [a]	1.43 ± 0.00928	973.8 ± 16.47
**2**	**H-TEDA(BF$_4$)$_2$**	90 [b]	1.47 ± 0.00426	934.9 ± 7.08
**3**		50 [b]	1.56 ± 0.00817	878.1 ± 11.90
**4**		20 [b]	1.68 ± 0.0200	796.7 ± 24.08
**5**		1.0 [b]	2.04 ± 0.0388	506.2 ± 22.93

[a] 70.25 mg of **H-TEDA(BF$_4$)$_2$**was transferred into a flask and then diluted with 1000 µL anhydrous and degassed CD_3CN. Due to moderate solubility, the concentration in CD_3CN is lower. [b] The concentrations are based on a 2000 µL stock solution dissolved in CD_3CN. 67.25 mg of **H-TEDA(BF$_4$)$_2$**was transferred into a flask and then diluted with 2000 µL anhydrous and degassed CD_3CN.

Based on the calculated volumes of **H-TEDA(BF$_4$)$_2$**, it can be determined that the volumes of **H-TEDA(BF$_4$)$_2$** increase significantly at higher concentrations. At the concentration for synthetic reactions conditions of 209 mM, **H-TEDA(BF$_4$)$_2$** has a volume of 973.8 ± 16.47 Å^3 (Table S18 entry 1). If the concentration is reduced to 1 mM, the volume is reduced to 506.2 ± 22.93 Å^3 (Table S18 entry 5). This corresponds to a decrease of approx. 48 % in **H-TEDA(BF$_4$)$_2$** volume. The increase in volume at elevated concentrations verifies the aggregation of **H-TEDA(BF$_4$)$_2$**in CD_3CN. In 2022, a study from our group[1] revealed that **SF®** aggregates as pure compound in CD_3CN *via* DOSY experiments. To verify whether an increased concentration of **H-TEDA(BF$_4$)$_2$** leads to aggregation or de-aggregation of **SF®** in the reaction mixture, the volumes of pure **SF®** and the volumes of a 1:1 mixture of **SF®** and **H-TEDA(BF$_4$)$_2$** were investigated. Concentrations of 90 mM were examined to ensure complete dissolution of the components in CD_3CN.

Table S19: Amounts of **SF®** and **H-TEDA(BF$_4$)$_2$**for DOSY studies of pure **SF®** and in a 1:1 reaction mixture in anhydrous and degassed CD_3CN at 308 K.

entry	compound(s)	concentration [mM]	amount of substrate [µmol]	mass [mg][a]
**6**	**SF®**	90	90	31.88
**7**	**SF®** / **H-TEDA(BF$_4$)$_2$**	90 / 90	SF®: 90<br>H-TEDA(BF$_4$)$_2$: 90	SF®: 31.88<br>H-TEDA(BF$_4$)$_2$: 30.26

[a] The amounts are dissolved in 1000 µL dry and degassed CD_3CN.

Table S20: Mean diffusion coefficients and calculated average volumes of **SF®** and **SF-H** as pure compounds and in a 1:1 reaction mixture at concentrations of 90 mM in anhydrous and degassed CD_3CN at 308 K.

entry	compound(s)	concentration [mM]	mean diffusion coefficient [E-9 m^2/s] with SD	average volume with SD [$Å^3$]
**6**	**SF®**	90	1.49 ± 0.00971	718.0 ± 11.80
**2**[a]	**H-TEDA(BF_4)$_2$**	90	1.47 ± 0.00426	934.9 ± 7.08
**7**	**SF® / H-TEDA(BF_4)$_2$**	90 / 90	SF®: 1.35 ± 0.00452<br>H-TEDA(BF_4)$_2$: 1.35 ± 0.0144	SF®: 1054.9 ± 9.13<br>H-TEDA(BF_4)$_2$: 1111.1 ± 30.87

[a] The values of **H-TEDA(BF_4)$_2$** are based on Table S18 entry 2.

SF® and **H-TEDA(BF_4)$_2$** were found to have smaller volumes as pure compounds than in 1:1 mixture. While **SF®** has a volume of 718.0 ± 11.80 $Å^3$ (see Table S17 entry 6) as pure compound in CD_3CN, the volume of **SF®** is increased to 1054.9 ± 9.13$Å^3$ (see Table S17 entry 8) in the reaction mixture. The same trend can be seen by **H-TEDA(BF_4)$_2$**. While **H-TEDA(BF_4)$_2$** has a volume of 934.9 ± 7.08 $Å^3$ (see Table S17 entry 2) as pure compound in CD_3CN, the volume of **H-TEDA(BF_4)$_2$** is increased to 1111.09 ± 30.87 $Å^3$ (see Table S17 entry 7) in the reaction mixture. It can be hypothesized that intermolecular interactions between **SF®** and

H-TEDA(BF_4)$_2$ lead to an increase in volume. In order to support this assumption, further DOSY experiments were performed in which the concentration of **SF®** remained constant while the concentration of

H-TEDA(BF_4)$_2$ was gradually increased.

Table S21: Calculated amounts of **SF®** and **H-TEDA(BF_4)$_2$** for 80 mM stock solutions.

entry	compound	concentration [mM]	amount of substrate [µmol]	mass [mg][a]
**8-11**	**SF®**	80	200	70.85
	**H-TEDA(BF_4)$_2$**	80	200	67.25

[a] The amounts are dissolved in 2500 µL dry and degassed CD_3CN.

Table S22: Mean diffusion coefficients and calculated average volumes of **SF®** and **H-TEDA(BF$_4$)$_2$** in a reaction mixture with different concentrations of **H-TEDA(BF$_4$)$_2$** in anhydrous and degassed CD_3CN at 308 K.

entry	compounds	concentrations of SF®: H-TEDA(BF$_4$)$_2$ [mM][b]	mean diffusion coefficients of SF® and H-TEDA(BF$_4$)$_2$ with SD [E-9 m^2/s]	average volume with SD [Å^3]
**8**	**SF®** / **H-TEDA(BF$_4$)$_2$**	40:40	**SF®**: 1.54 ± 0.0359<br>**H-TEDA(BF$_4$)$_2$**: 1.54 ± 0.0295	**SF®**: 1045.2 ± 61.8<br>**H-TEDA(BF$_4$)$_2$**: 1040.1 ± 50.8
**9**		40:30	**SF®**: 1.62 ± 0.0246<br>**H-TEDA(BF$_4$)$_2$**: 1.64 ± 0.0171	**SF®**: 812.9 ± 31.2<br>**H-TEDA(BF$_4$)$_2$**: 791.8 ± 21.0
**10**		40:20	**SF®**: 1.60 ± 0.0176<br>**H-TEDA(BF$_4$)$_2$**: 1.65 ± 0.0197	**SF®**: 762.7 ± 21.3<br>**H-TEDA(BF$_4$)$_2$**: 709.6 ± 21.2
**11**		40:10	**SF®**: 1.65 ± 0.0187<br>**H-TEDA(BF$_4$)$_2$**: 1.71 ± 0.0021	**SF®**: 765.5 ± 21.9<br>**H-TEDA(BF$_4$)$_2$**: 695.6 ± 21.5

[b] The amounts are based on 2500 µL stock solutions in Table S21.

To investigate the influence of **H-TEDA(BF$_4$)$_2$** loading in the reaction solution, the stock solutions of **SF®** and **H-TEDA(BF$_4$)$_2$** were prepared with concentrations of 80 mM in anhydrous and degassed CD_3CN. The corresponding amounts of the compounds (see Table S21) were transferred into flasks and then diluted with 2500 µL anhydrous and degassed CD_3CN. The stock solutions were degassed *via* freeze-pump-thaw method and stored inside the glove box. Depending on the **H-TEDA(BF$_4$)$_2$** loading (40 mM – 10 mM) to be examined (see Table S22), the corresponding volumes of **SF®** and **H-TEDA(BF$_4$)$_2$** were transferred into the NMR tube. Anhydrous and degassed TMS was added to the samples inside the glove box. By raising **H-TEDA(BF$_4$)$_2$** loading in 10 mM (see Table S22) steps, the calculated volumes of **SF®** and **H-TEDA(BF$_4$)$_2$** increase. While **SF®** has a volume of 765.5 ± 21.9 Å^3 (see Table S22 entry 11) in a 4:1 **SF®**: **H-TEDA(BF$_4$)$_2$** reaction mixture, the volume of **SF®** increases to 1045.2 ± 61.8 Å^3 (see Table S22 entry 8) in a 1:1 **SF®**: **H-TEDA(BF$_4$)$_2$** reaction mixture. The same trend can be observed for **H-TEDA(BF$_4$)$_2$**. While **H-TEDA(BF$_4$)$_2$** has a volume of 695.6 ± 21.5Å^3 (see Table S22 entry 11) in a 4:1 **SF®**: **H-TEDA(BF$_4$)$_2$** reaction mixture, the volume of **H-TEDA(BF$_4$)$_2$** increases to 1040.1 ± 50.8 Å^3 (see Table S22 entry 8) in a 1:1 **SF®**:**H-TEDA(BF$_4$)$_2$** reaction mixture.

To verify whether **H-TEDA(BF$_4$)$_2$** is already present as monomer in the 1.0 mM sample (see Table S18 entry 5), the monomer volume was calculated based on the known intermolecular van der Waals radii of the corresponding functional groups and atoms using *Chem3D 20.1.1* software. A monomer volume of *321.2 Å^3* was determined for **H-TEDA(BF$_4$)$_2$**. The calculated volume differs from the calculated DOSY value (compare Table S18 entry 5), indicating that **H-TEDA(BF$_4$)$_2$** aggregates even at low concentrations of 1.0 mM.

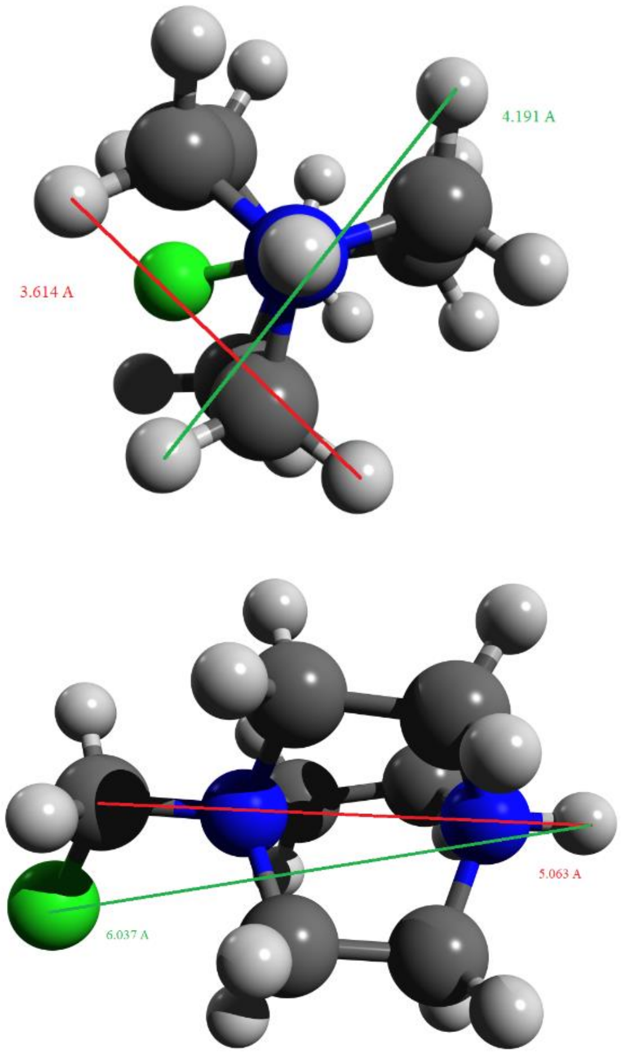

Figure S10: Distance calculations using *Chem3D 20.1.1* to calculate the monomer volume of **H-TEDA(BF_4)$_2$.**

4.1.5 DOSY studies of photochemical C(sp^3)-H fluorination reactions under standard conditions and with **H-TEDA(BF$_4$)$_2$** loading

In order to determine the volume change of the individual compounds during the reaction, a photochemical C(sp^3)-H fluorination reaction was measured under standard conditions and with **H-TEDA(BF$_4$)$_2$** loading (see Scheme S4). To investigate the volumes of **SF®**, substrate and **H-TEDA(BF$_4$)$_2$** during the photochemical reaction, suppressing 1D double-stimulated echo experiments (details see 4.1.2) and ^{1}H NMR experiments were performed alternately during *in situ* illumination of the reaction **1** and **2**.

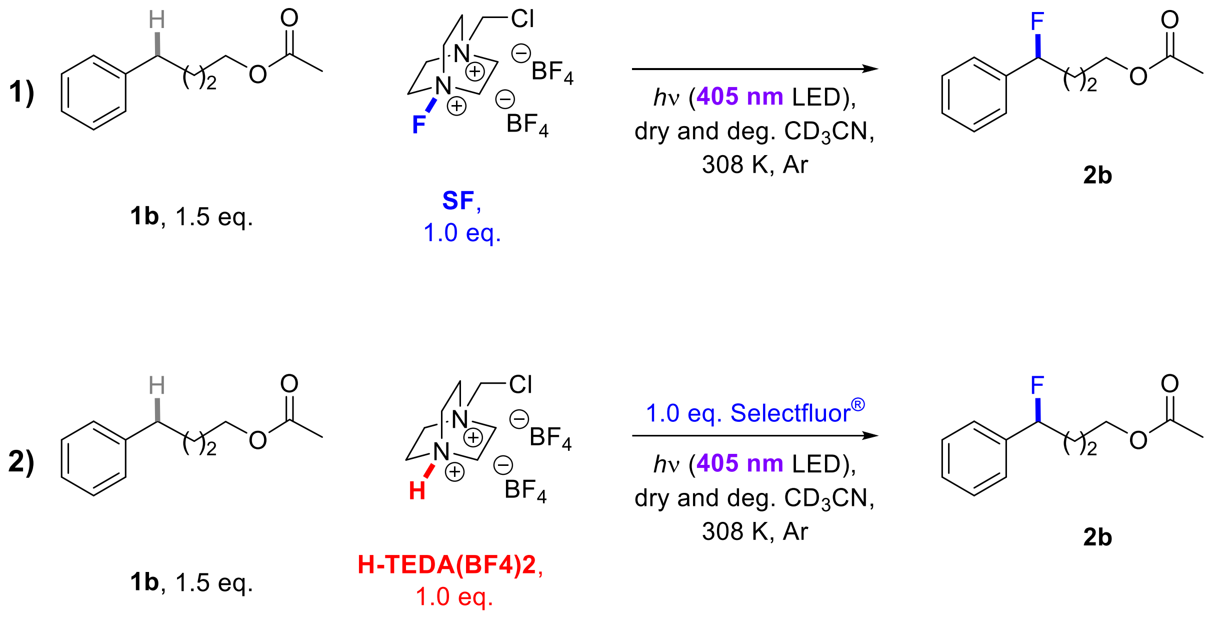

Scheme S4: Model reaction to determine the volumes of **SF®**, 1b and **H-TEDA(BF$_4$)$_2$** during the photochemical C(sp^3)-H fluorination *via in situ* illumination DOSY experiments. Reaction **1)** Photochemical fluorination under standard conditions (without **H-TEDA(BF$_4$)$_2$**); Reaction **2)** Photochemical fluorination reaction with 1.0 eq. **H-TEDA(BF$_4$)$_2$** loading.

In order to ensure identical reaction conditions for the DOSY experiments, the NMR samples were prepared out of stock solutions. The respective amounts for the stock solutions are given in Table S23. The entire NMR sample preparation for both experiments were performed under inert gas conditions. The actual preparation for the reaction solutions were carried out inside the glove box. Anhydrous and degassed TMS was added to the samples inside the glove box. Lastly, the *in situ* illumination insert was combined with the NMR tube as described in 1.4.1. Mean diffusion coefficients and calculated average volumes of the compounds are given in Table S24 and Table S25 for reaction **1** and **2**, respectively.

Table S23: Calculated amounts of **SF®**, **H-TEDA(BF$_4$)$_2$**and 1b for *in situ* illumination DOSY studies.

compound	equivalents	concentration [mM]	amount of substrate [µmol][a]	mass [mg]
**1b** [a]	1.5	45	67.5	12.98 (12.67 µl)
**SF®** [a]	1.0	30	45	15.94
**H-TEDA(BF$_4$)$_2$** [a]	1.0	30	45	15.13

[a] The amounts are based on 500 µl stock solutions in dry and degassed CD$_3$CN.

Table S24: Mean diffusion coefficients and calculated average volumes of **SF®**, **H-TEDA(BF$_4$)$_2$** and substrate 1b in the reaction mixture with 1.0 equivalent **H-TEDA(BF$_4$)$_2$** loading (see Scheme S4-2) at different times in anhydrous and degassed CD$_3$CN at 308 K.

entry	~ time [min]	compound	mean diffusion coefficients of SF®, H-TEDA(BF$_4$)$_2$ and 1b [E-9 m^2/s]	average volume [Å^3]
		**SF®**	1.54 ± 0.032	745.0
**1**	0 (no illumination)	**H-TEDA(BF$_4$)$_2$**	1.55 ± 0.042	726.5
		**1b**	2.33 ± 0.075	271.3
		**SF®**	1.54 ± 0.036	764.6
**2**	38	**H-TEDA(BF$_4$)$_2$**	1.56 ± 0.042	743.2
		**1b**	2.34 ± 0.067	277.0
		**SF®**	1.54 ± 0.034	771.6
**3**	96	**H-TEDA(BF$_4$)$_2$**	1.56 ± 0.041	752.7
		**1b**	2.39 ± 0.057	267.0
		**SF®**	1.54 ± 0.036	746.7
**4**	154	**H-TEDA(BF$_4$)$_2$**	1.56 ± 0.042	736.0
		**1b**	2.39 ± 0.57	264.1
		**SF®**	1.54 ± 0.036	752.4
**5**	211	**H-TEDA(BF$_4$)$_2$**	1.56 + 0.042	747.5
		**1b**	2.39 ± 0.058	275.0
		**SF®**	1.54 ± 0.035	752.4
**6**	269	**H-TEDA(BF$_4$)$_2$**	1.56 ± 0.041	733.9
		**1b**	2.33 ± 0.076	275.0
		**SF®**	1.54 ± 0.036	759.1
**7**	327	**H-TEDA(BF$_4$)$_2$**	1.55 ± 0.041	740.7
		**1b**	2.34 ± 0.234	273.3
		**SF®**	1.54 ± 0.035	758.7
**8**	385	**H-TEDA(BF$_4$)$_2$**	1.56 ± 0.041	742.5
		**1b**	2.38 ± 0.058	265.0
		**SF®**	1.54 ± 0.036	754.0
**9**	443	**H-TEDA(BF$_4$)$_2$**	1.55 ± 0.042	742.0
		**1b**	2.39 ± 0.059	261.0
		**SF®**	1.54 ± 0.035	762.5
**10**	501	**H-TEDA(BF$_4$)$_2$**	1.55 ± 0.042	751.2
		**1b**	2.34 ± 0.065	276.6
		**SF®**	1.54 ± 0.034	783.0
**11**	559	**H-TEDA(BF$_4$)$_2$**	1.55 ± 0.040	770.8
		**1b**	2.33 ± 0.067	284.8
		**SF®**	1.54 ± 0.035	785.9
**12**	617	**H-TEDA(BF$_4$)$_2$**	1.55 ± 0.041	772.4
		**1b**	2.34 ± 0.065	282.6
		**SF®**	1.54 ± 0.035	785.8
**13**	674	**H-TEDA(BF$_4$)$_2$**	1.55 ± 0.041	771.4
		**1b**	2.34 ± 0.074	282.7
		**SF®**	1.54 ± 0.036	775.3
**14**	731	**H-TEDA(BF$_4$)$_2$**	1.55 ± 0.041	760.8
		**1b**	2.33 ± 0.067	280.1

		**SF®**	1.54 ± 0.035	790.3
**15**	789	**H-TEDA(BF$_4$)$_2$**	1.55 ± 0.041	774.6
		**1b**	2.38 ± 0.057	272.0
		**SF®**	1.54 ± 0.036	794.0
**16**	847	**H-TEDA(BF$_4$)$_2$**	1.55 ± 0.041	774.8
		**1b**	2.34 ± 0.073	284.1

Table S25: Mean diffusion coefficients and calculated average volumes of **SF®**, **H-TEDA(BF$_4$)$_2$** and 1b in the reaction mixture under standard conditions (see Scheme S4-1) at different times in anhydrous and degassed CD_3CN at 308 K.

entry	~ time [min]	compound	mean diffusion coefficients of SF®, H-TEDA(BF$_4$) and substrate [E-9 m^2/s]	average volume [Å^3]
		**SF®**	1.61 ± 0.039	689.8
**1**	0 (no illumination)	**H-TEDA(BF$_4$)$_2$**	2.38 ± 0.084	267.3
		**1b**	2.37 ± 0.076	280.0
		**SF®**	1.62 ± 0.037	715.3
**2**	38	**H-TEDA(BF$_4$)$_2$**	2.36 ± 0.11	287.9
		**1b**	2.41 ± 0.050	275.1
		**SF®**	1.62 ± 0.038	764.3
**3**	96	**H-TEDA(BF$_4$)$_2$**	2.18 ± 0.11	366.7
		**1b**	2.41 ± 0.048	289.6
		**SF®**	1.61 ± 0.038	736.4
**4**	154	**H-TEDA(BF$_4$)$_2$**	2.13 ± 0.086	372.6
		**1b**	2.39 ± 0.074	284.6
		**SF®**	1.62 ± 0.037	730.4
**5**	211	**H-TEDA(BF$_4$)$_2$**	2.04 ± 0.091	408.1
		**1b**	2.40 ± 0.072	281.3
		**SF®**	1.61 ± 0.038	732.4
**6**	269	**H-TEDA(BF$_4$)$_2$**	2.05 ± 0.091	401.2
		**1b**	2.40 ± 0.047	293.9
		**SF®**	1.62 ± 0.039	725.0
**7**	327	**H-TEDA(BF$_4$)$_2$**	2.14 ± 0.079	386.4
		**1b**	2.40 ± 0.048	279.6
		**SF®**	1.61 ± 0.037	725.9
**8**	385	**H-TEDA(BF$_4$)$_2$**	1.99 ± 0.086	419.8
		**1b**	2.40 ± 0.067	279.6
		**SF®**	1.62 ± 0.038	713.1
**9**	443	**H-TEDA(BF$_4$)$_2$**	1.92 ± 0.070	466.1
		**1b**	2.42 ± 0.072	272.9
		**SF®**	1.62 ± 0.038	717.4
**10**	501	**H-TEDA(BF$_4$)$_2$**	2.02 ± 0.075	416.6
		**1b**	2.40 ± 0.074	277.4
		**SF®**	1.62 ± 0.038	724.8
**11**	559	**H-TEDA(BF$_4$)$_2$**	2.02 ± 0.065	419.8
		**1b**	2.44 ± 0.059	267.9

		**SF®**	1.62 ± 0.038	721.5
**12**	617	**H-TEDA(BF_4)$_2$**	1.93 ± 0.089	454.3
		**1b**	2.40 ± 0.072	280.3
		**SF®**	1.62 ± 0.039	735.7
**13**	674	**H-TEDA(BF_4)$_2$**	1.98 ± 0.072	441.9
		**1b**	2.39 ± 0.071	283.9
		**SF®**	1.62 ± 0.041	725.0
**14**	731	**H-TEDA(BF_4)$_2$**	1.91 ± 0.078	479.4
		**1b**	2.39 ± 0.071	280.5
		**SF®**	1.62 ± 0.037	721.4
**15**	789	**H-TEDA(BF_4)$_2$**	1.97 ± 0.063	464.9
		**1b**	2.39 ± 0.073	280.4
		**SF®**	1.62 ± 0.038	711.4
**16**	847	**H-TEDA(BF_4)$_2$**	1.90 ± 0.049	476.4
		**1b**	2.40 ± 0.070	275.0
		**SF®**	1.61 ± 0.038	741.8
**17**	905	**H-TEDA(BF_4)$_2$**	1.90 ± 0.063	496.7
		**1b**	2.40 ± 0.072	280.0
		**SF®**	1.61 ± 0.039	758.3
**18**	963	**H-TEDA(BF_4)$_2$**	1.91 ± 0.059	493.9
		**1b**	2.44 ± 0.066	276.6
		**SF®**	1.60 ± 0.028	754.9
**19**	1021	**H-TEDA(BF_4)$_2$**	1.86 ± 0.059	505.6
		**1b**	2.39 ± 0.075	283.7
		**SF®**	1.62 ± 0.037	762.3
**20**	1079	**H-TEDA(BF_4)$_2$**	1.89 ± 0.047	515.1
		**1b**	2.43 ± 0.05	283.3
		**SF®**	1.60 ± 0.038	750.8
**21**	1137	**H-TEDA(BF_4)$_2$**	1.87 ± 0.059	519.9
		**1b**	2.40 ± 0.074	274.5
		**SF®**	1.59 ± 0.038	746.6
**22**	1195	**H-TEDA(BF_4)$_2$**	1.86 ± 0.054	524.5
		**1b**	2.40 ± 0.071	273.3
		**SF®**	1.59 ± 0.028	743.7
**23**	1253	**H-TEDA(BF_4)$_2$**	1.85 ± 0.038	540.1
		**1b**	2.39 ± 0.070	273.3
		**SF®**	1.62 ± 0.039	740.8
**24**	1311	**H-TEDA(BF_4)$_2$**	1.80 ± 0.055	570.0
		**1b**	2.39 ± 0.067	286.1
		**SF®**	1.62 ± 0.038	737.7
**25**	1369	**H-TEDA(BF_4)$_2$**	1.80 ± 0.042	580.0
		**1b**	2.40 ± 0.073	283.5
		**SF®**	1.62 ± 0.037	762.2
**26**	1427	**H-TEDA(BF_4)$_2$**	1.80 ± 0.047	578.8
		**1b**	2.40 ± 0.074	293.3

In order to determine the change in volumes of the reagents during photochemical C(sp^3)-H fluorination reactions, mean diffusion coefficients were determined in one-hour steps during *in situ* illumination. The volumes were calculated on the basis of the mean diffusion coefficients and are summarized in Figure S11.

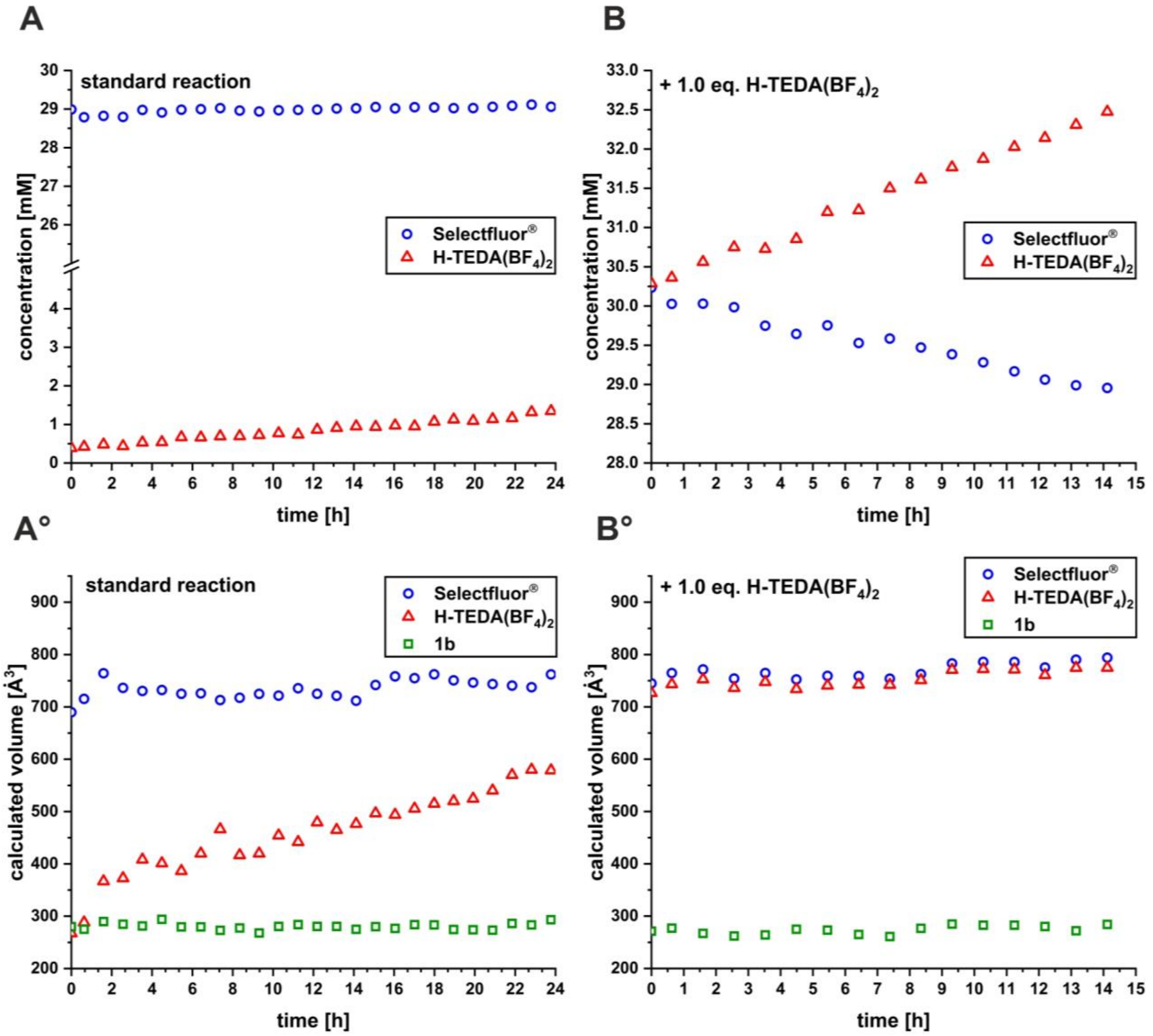

Figure S11: (A/B): Concentration monitoring of the photochemical C(sp^3)-H fluorination of **1b** (see Scheme S4) during *in situ* illumination with different **H-TEDA(BF_4)$_2$** loading. (A°/B°): Calculated average volumes of **SF®**, **H-TEDA(BF_4)$_2$** and **1b** without **H-TEDA(BF_4)$_2$** added (A°) and with 1.0 eq. of **H-TEDA(BF_4)$_2$** added (B°) before and during *in situ* illumination. A small quantity of **H-TEDA(BF_4)$_2$** is present prior to *in situ* illumination under standard conditions due to the fact that **SF®** as substance contains traces of **H-TEDA(BF_4)$_2$**.

SF® has a calculated volume of 689.8 Å and **H-TEDA(BF_4)$_2$** has a calculated volume of 267.3 Å before *in situ* illumination in the standard reaction (see Table S25, entry 1). Meanwhile, **SF®** and **H-TEDA(BF_4)$_2$** exhibit volumes of 745.0 Å and 726.5 Å before *in situ* illumination in the reaction mixture with 1.0 eq. **H-TEDA(BF_4)$_2$** loading (see Table S24, entry 1). The volume of **1b** remains almost unchanged in both reactions before and during *in situ* illumination. It is assumed that the substrate **1b** does not affect **SF®** and **H-TEDA(BF_4)$_2$** before and during reaction **1** and **2**. During the reaction with **H-TEDA(BF_4)$_2$** loading, the volumes of **SF®** and **H-TEDA(BF_4)$_2$** do not significantly change (see Figure S11-B°).

5 Mechanistic study

5.1 Characterization of intermolecular interactions between **SF®** and **H-TEDA(BF$_4$)$_2$** with 1D and 2D NMR experiments

For the elucidation of intermolecular interactions, **SF®**, **H-TEDA(BF$_4$)$_2$** and the 1:1 mixture of both components was investigated by low temperature NMR experiments at 230 K. Such low temperatures are necessary to slow down possible exchange processes and to monitor possible hydrogen-bonding interactions between **SF®** and **H-TEDA(BF$_4$)$_2$**. For this, we first determined the ^{1}H NMR spectra of the pure components and a 1:1 mixture of the components at 230 K in CD_3CN. The actual preparation of the NMR samples was done inside the glove box. The weights of the components for each experiment are given in Table S26.

Table S26: Calculated amounts for the investigation of of **SF®** and **H-TEDA(BF$_4$)$_2$**.

entry	compound(s)	equivalents	concentration [mM]	amount of substrate [µmol][a]	mass [mg][a]
**1**	**SF®**	1.0	90	63	22.32
**2**	**H-TEDA(BF$_4$)$_2$**	1.0	90	63	21.19
**3**	**SF®** / **H-TEDA(BF$_4$)$_2$**	1.0 / 1.0	90 / 90	63 / 63	22.32 / 21.19

[a]The amounts are dissolved in 700 µL dry and degassed CD_3CN.

The corresponding amounts of compounds (see Table S26) were transferred into flasks and then dissolved in 700 µL anhydrous and degassed CD_3CN. The samples were degassed using the *freeze-pump-thaw* method and stored inside the glove box. Entry 1-3 were transferred into separate NMR tubes inside the glove box and anhydrous and degassed TMS as internal standard was added.

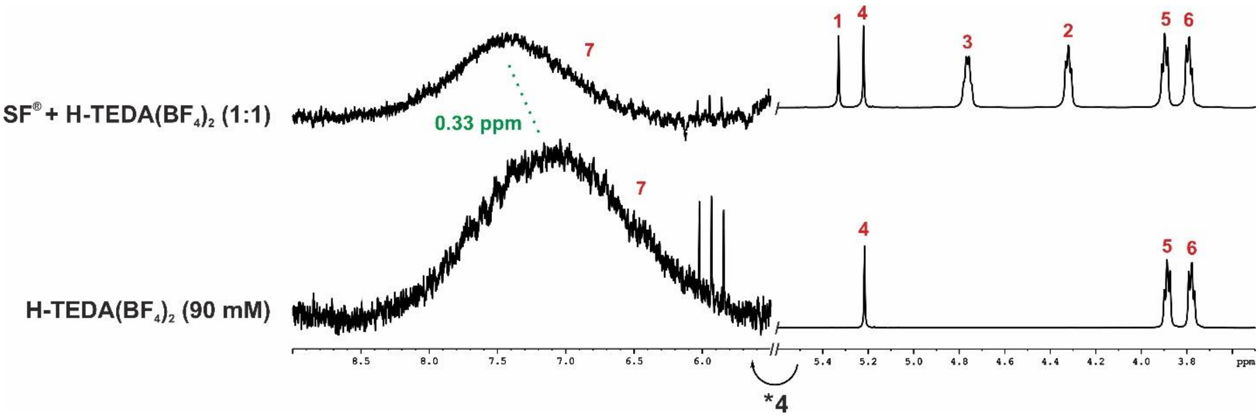

Figure S12: Comparison between the chemical shifts of the ^{1}H NMR spectra of pure **H-TEDA(BF$_4$)$_2$** (90 mM) and the 1:1 mixture (90:90 mM) at 298 K. The spectra were referenced to TMS (tetramethylsilane) at δ(^{1}H) = 0.00 ppm

It can be observed that the signals of **H-TEDA(BF$_4$)$_2$** change to lower chemical shifts in the reaction mixture. The R_3N^+-H signal (7) of pure **H-TEDA(BF$_4$)$_2$**, which is observed as broad singlet at 7.11 ppm shifts to 7.45 ppm in the reaction mixture. Further, the R_3N^+-H proton signal (7) is broadened in the mixture due to chemical exchange processes.[14] Therefore, it is assumed that the formation of a intermolecular hydrogen bond of **SF®** and **H-TEDA(BF$_4$)$_2$** leads to a downfield shift of signal 7 in the 1:1 reaction mixture

(see Figure S12). The next step was to characterize the intermolecular interactions between **SF®** and **H-TEDA(BF_4)$_2$** in CD_3CN. Initially, we carried out 2D ^{1}H−^{1}H NOESY, ^{1}H−^{1}H ROESY and ^{1}H−^{19}F HOESY NMR experiments to determine the configuration of the complex *via* NOE pattern of the 1:1 mixture.

Pulse sequences and aquistion parameters for 2D experiments:

Phase-sensitive 2D-^{1}H-^{1}H NOESY: pulse program: noesygpph; relaxation delay d1 = 2 s; NS = 18; mixing time (d8) = 700.00 ms; TD = 4096; increments = 1024

Phase-sensitive 2D-^{1}H-^{1}H ROESY: pulse program: roesyph; relaxation delay d1 = 2.6 s; NS = 56; mixing time (low-power spin lock pulse p15) = 700.00 ms; TD = 4096; increments = 1024

2D-^{1}H-^{19}F HOESY: pulse program: hoesygpph, relaxation delay d1 = 5.0 s, NS = 128; mixing time (d8) = 300.00 ms; TD = 4096; increments = 128-256

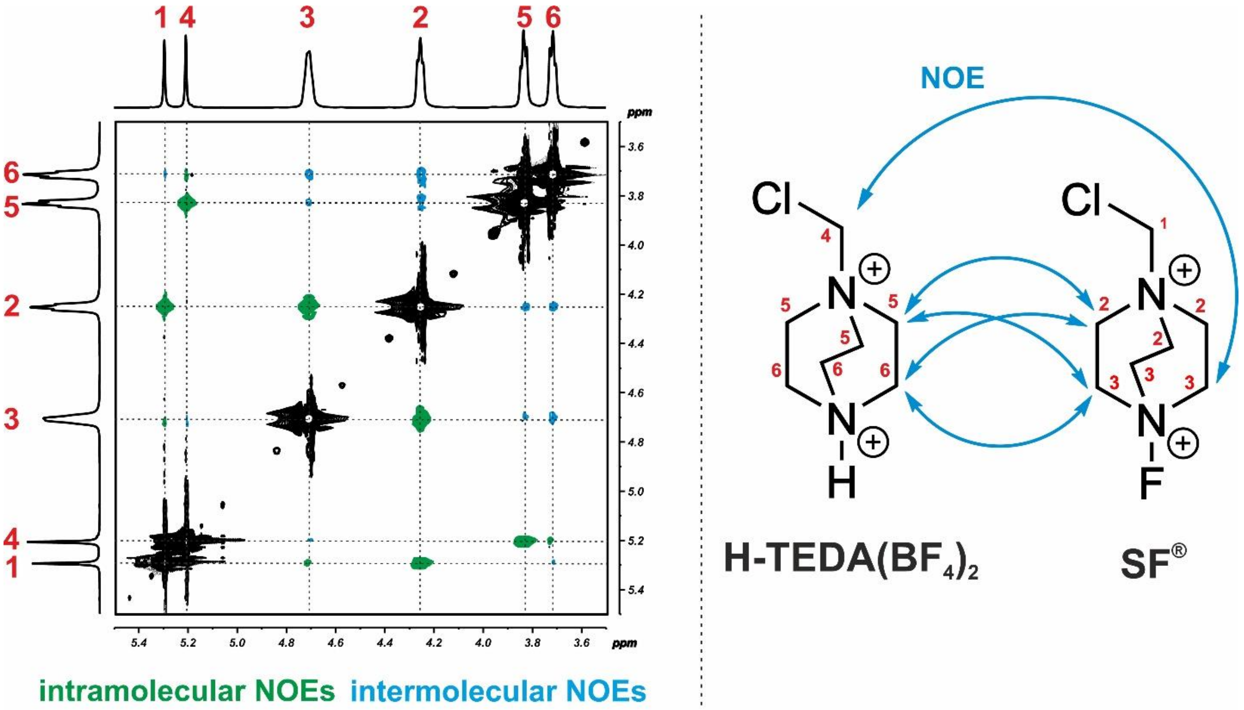

Figure S13: Section of the ^{1}H-^{1}H NOESY spectrum of **SF®** and **H-TEDA(BF_4)$_2$** at a 1:1 ratio with concentrations of 90 mM in dry and degassed CD_3CN at 230 K. Intramolecular NOE cross peaks of **SF®** and **H-TEDA(BF_4)$_2$** are marked in green and intermolecular NOE cross peaks (compare Figure S13-right) between both species are marked in blue. The spectra were referenced to TMS as internal standard.

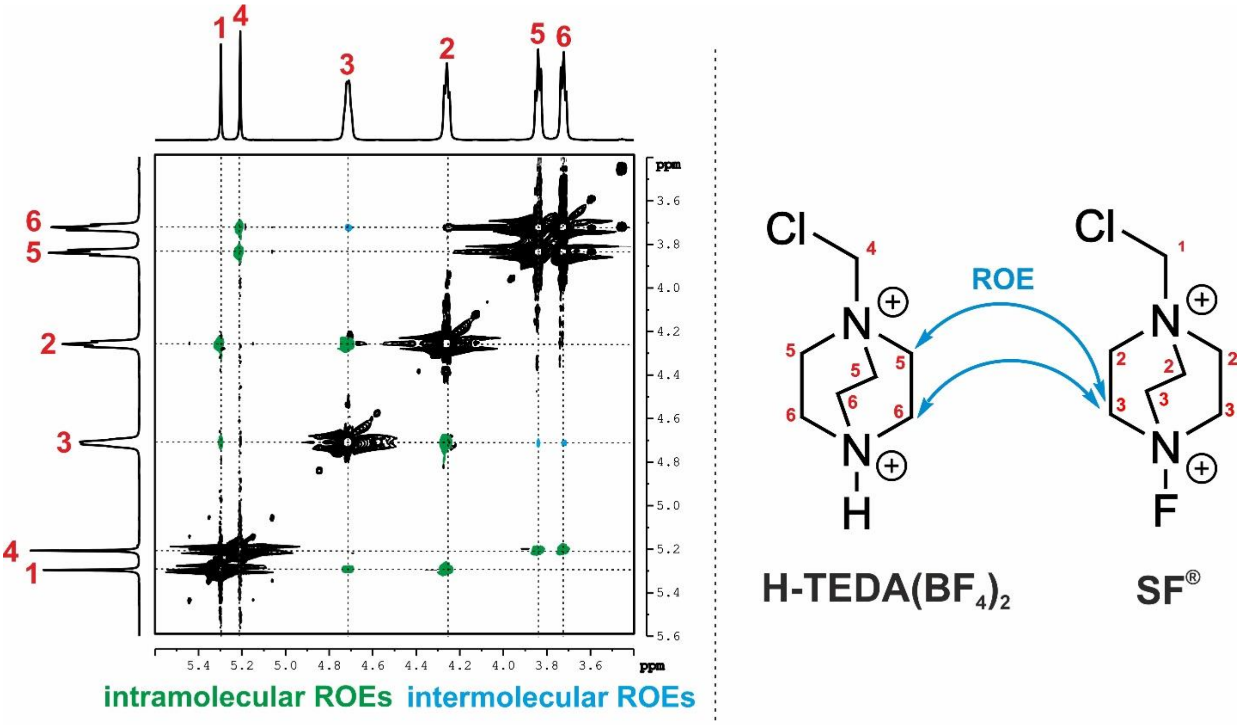

Figure S14: Section of the $^1H-^1H$ ROESY spectrum of **SF®** and **H-TEDA(BF$_4$)$_2$** at a 1:1 ratio with concentrations of 90 mM in dry and degassed CD_3CN at 230 K. Intramolecular NOE cross peaks of **SF®** and **H-TEDA(BF$_4$)$_2$** are marked in green and intermolecular NOE cross peaks (compare Figure S14-right) between **SF®** and **H-TEDA(BF$_4$)$_2$** are marked in blue. The spectra were referenced to TMS as internal standard.

Phase-sensitive 2D-$^1H-^1H$ NOESY and phase-sensitive 2D-$^1H-^1H$ ROESY experiments of the 1:1 mixture of **SF®** and **H-TEDA(BF$_4$)$_2$** reveale cross-peaks between the species. We assume hydrogen bond formation between **SF®** and **H-TEDA(BF$_4$)$_2$**. Cl and F are both potential H-bond acceptors for the formation of intermolecular hydrogen bonding (see Figure S15).

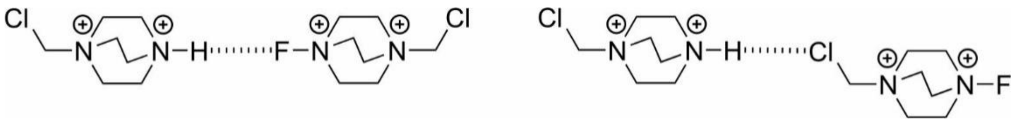

Figure S15: Possible hydrogen bond formations between **SF®** and **H-TEDA(BF$_4$)$_2$**.

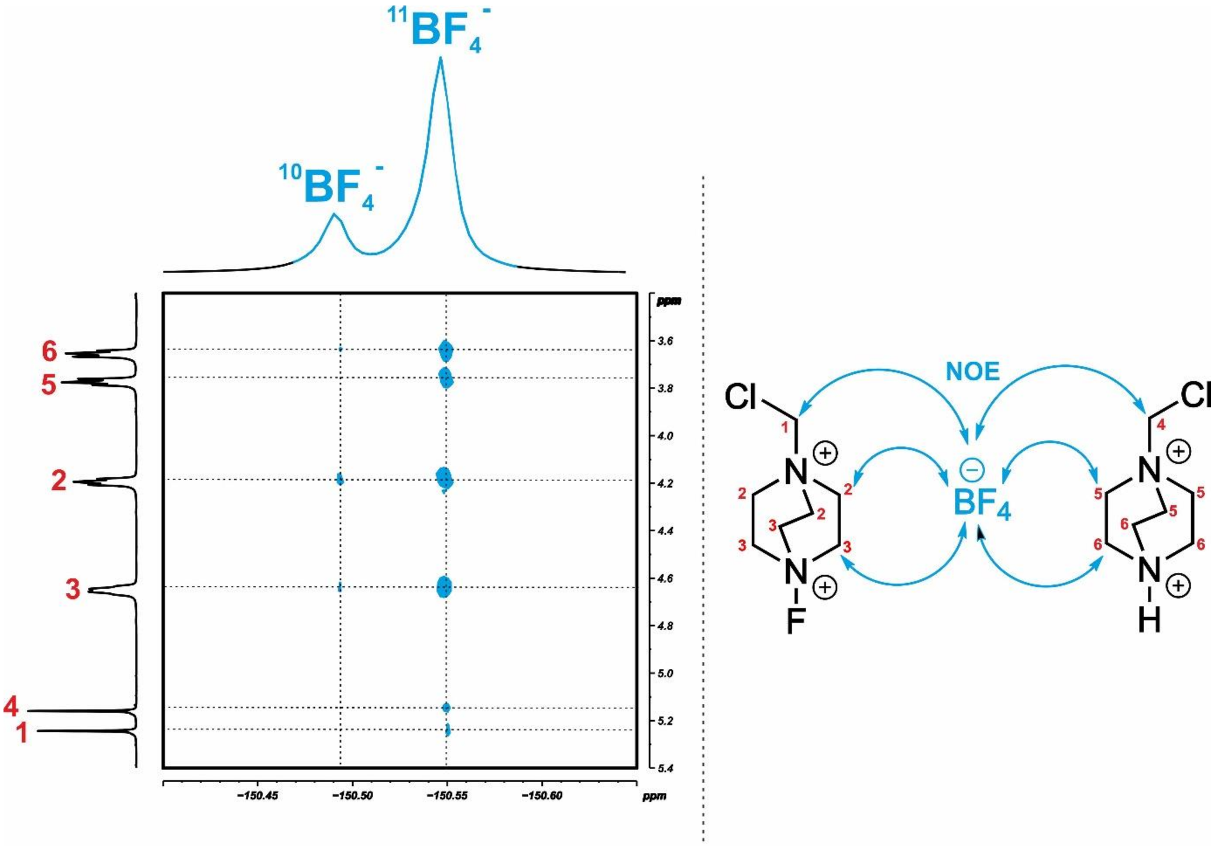

Figure S16: Section of the $^{1}H{-}^{19}F$ HOESY spectrum of **SF®** and **H-TEDA(BF$_4$)$_2$** at a 1:1 ratio with concentrations of 90 mM in dry and degassed CD_3CN at 230 K. Intermolecular NOE cross peaks between BF_4^- anion, **SF®** and **H-TEDA(BF$_4$)$_2$**. The spectra were referenced to TMS as internal standard.

The $^{1}H{-}^{19}F$ HOESY spectrum revealed NOE contacts between the anion (BF_4^-) and the cationic species of **SF®** and **H-TEDA(BF$_4$)$_2$**.

6 ^{1}H NMR, ^{13}C NMR and ^{19}F NMR Spectra

^{1}H NMR of compound **H-TEDA(BF$_4$)$_2$** in CD_3CN

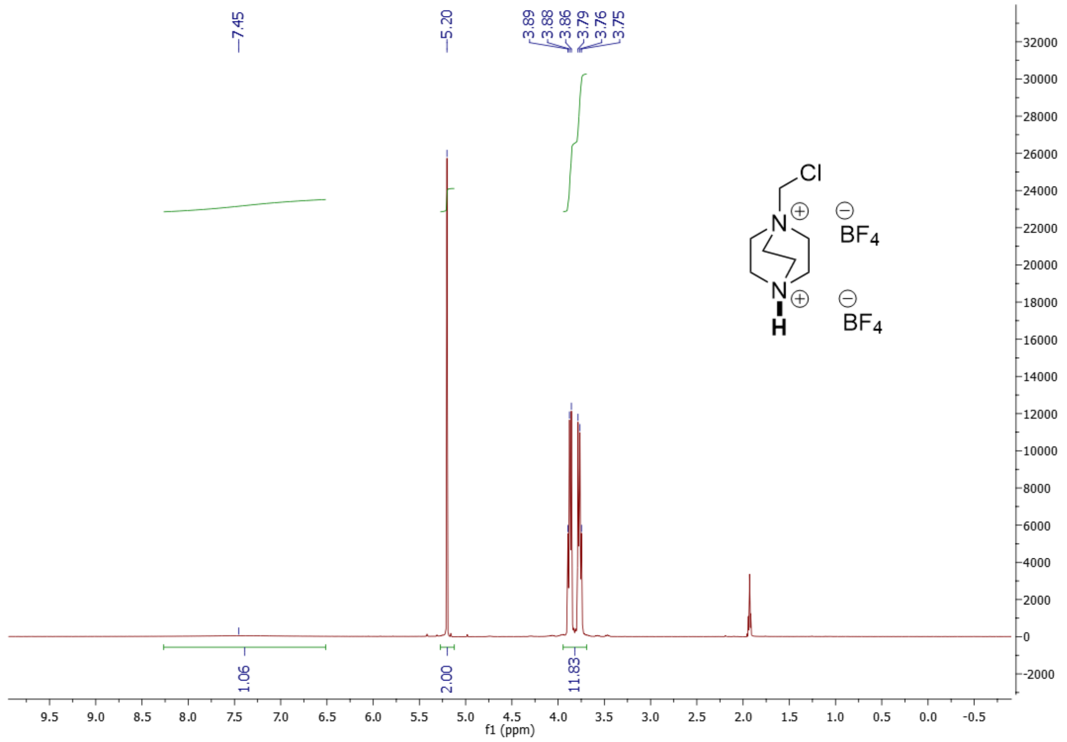

^{13}C NMR of compound **H-TEDA(BF$_4$)$_2$** in CD_3CN

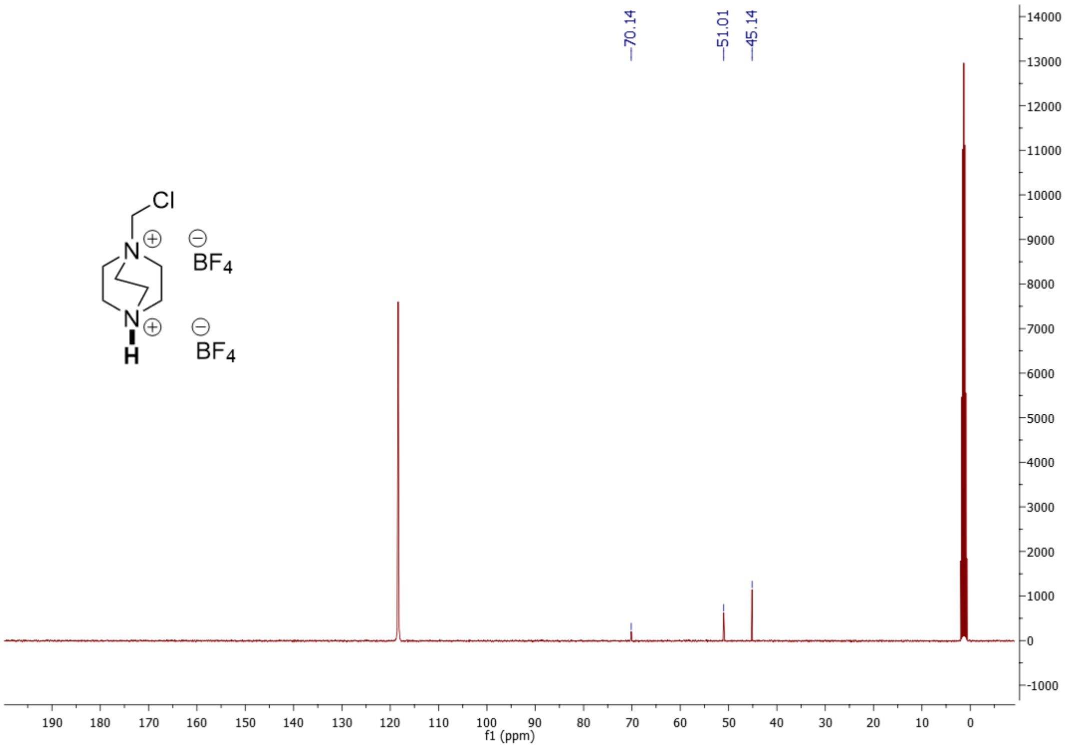

^{19}F NMR of compound **H-TEDA(BF4)2** in CD3CN

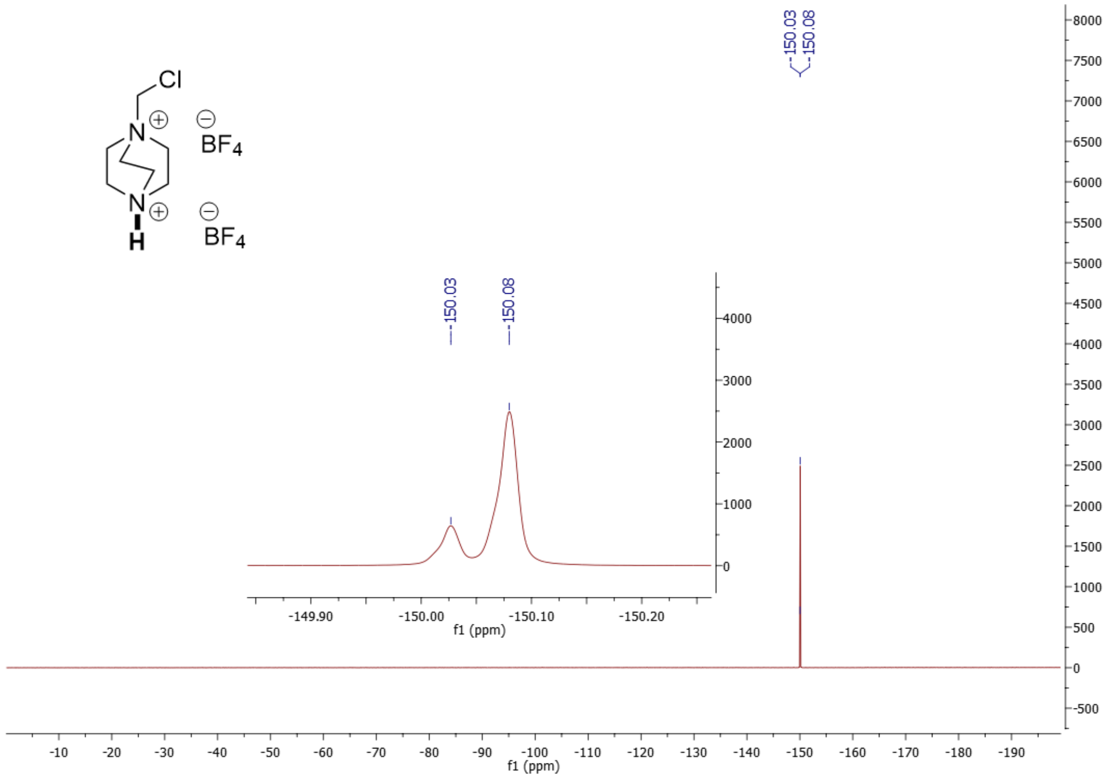

^{1}H NMR of compound **1d** in CDCl3

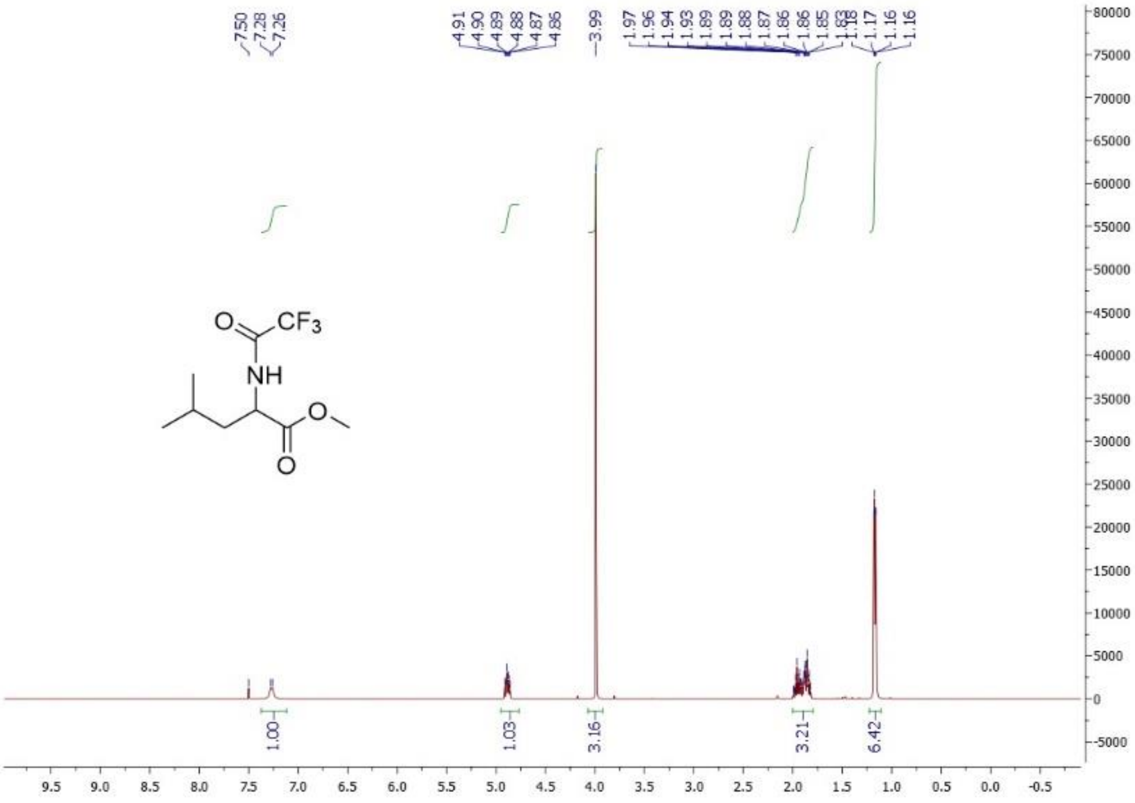

^{13}C NMR of compound **1d** in $CDCl_3$

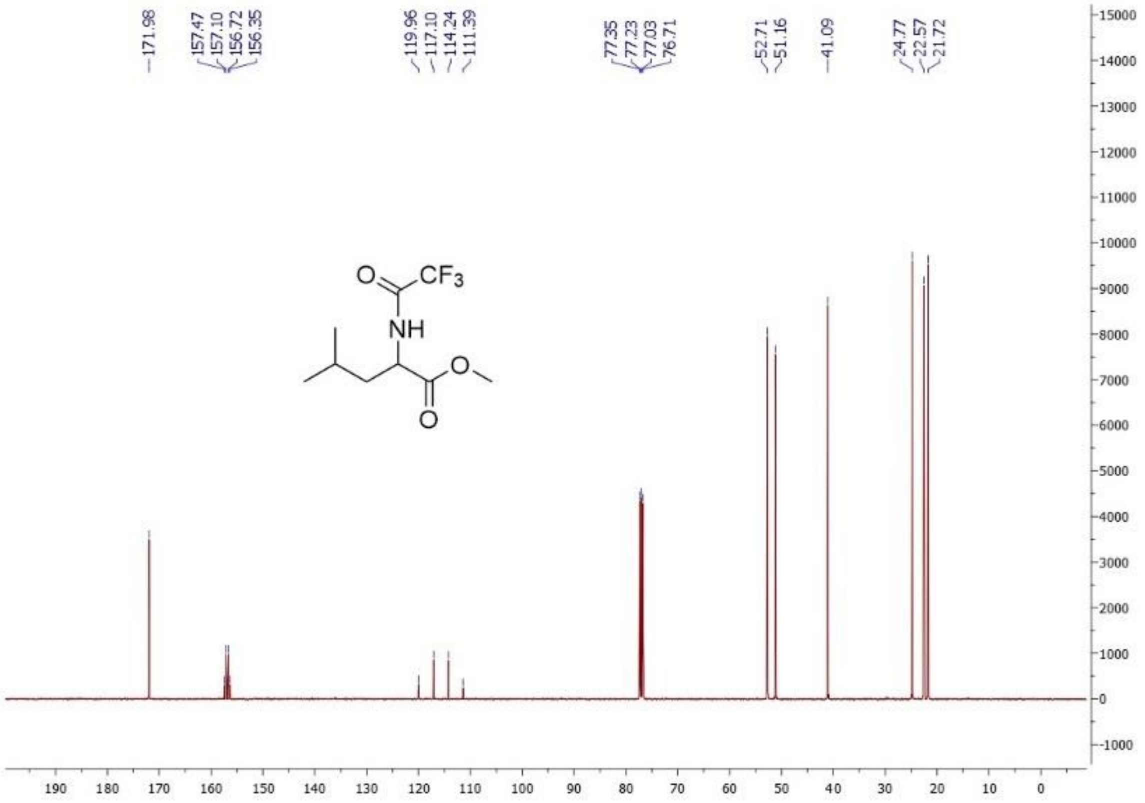

^{19}F NMR of compound **1d** in $CDCl_3$

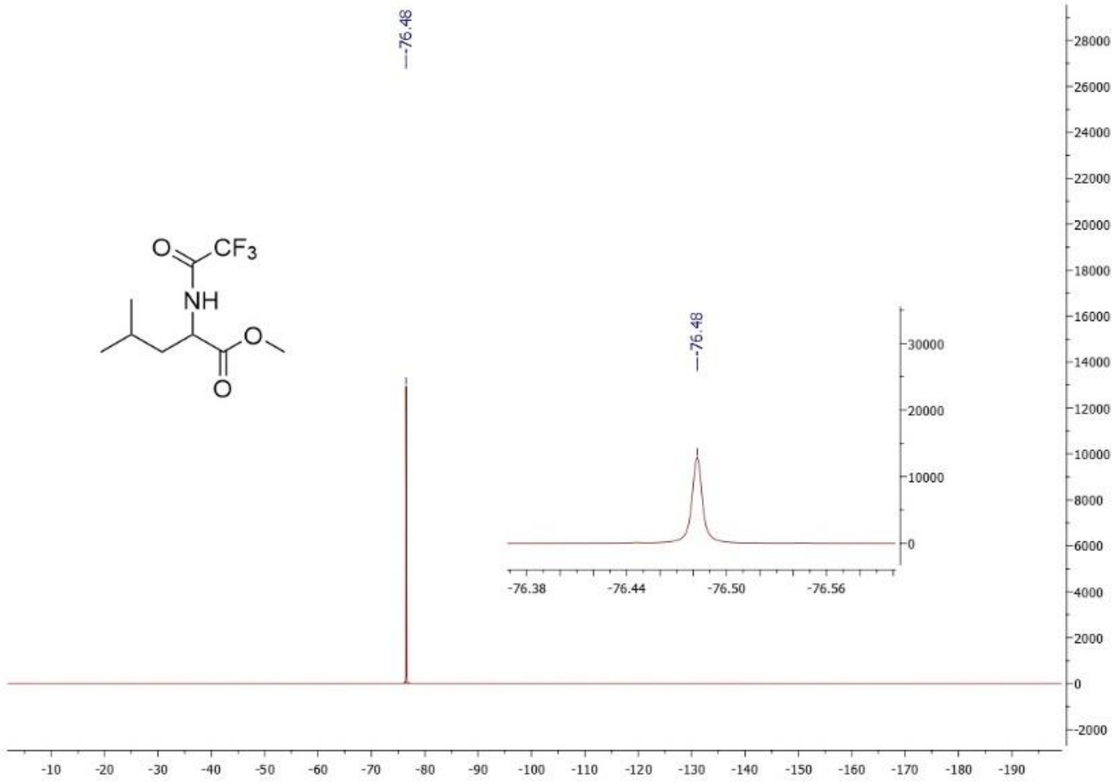

^{1}H NMR of compound **TEA-H·BF$_4$** in D_2O

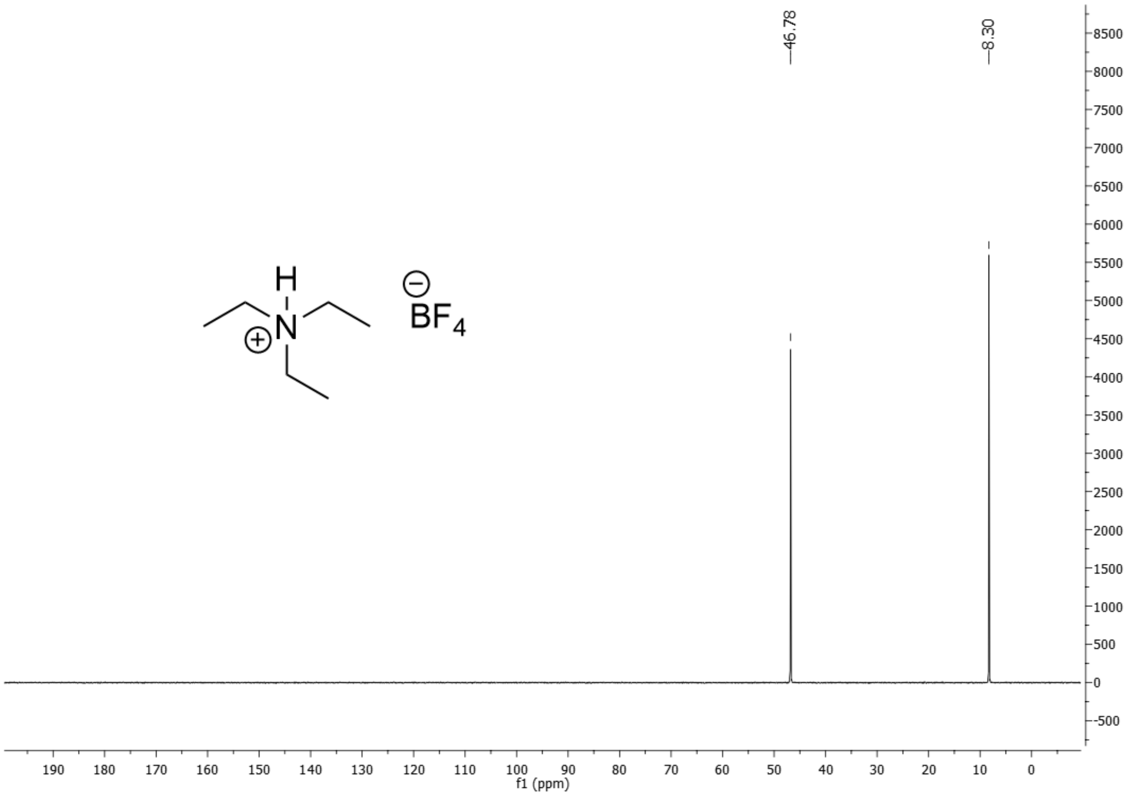

^{13}C NMR of compound **TEA-H·BF$_4$** in D_2O

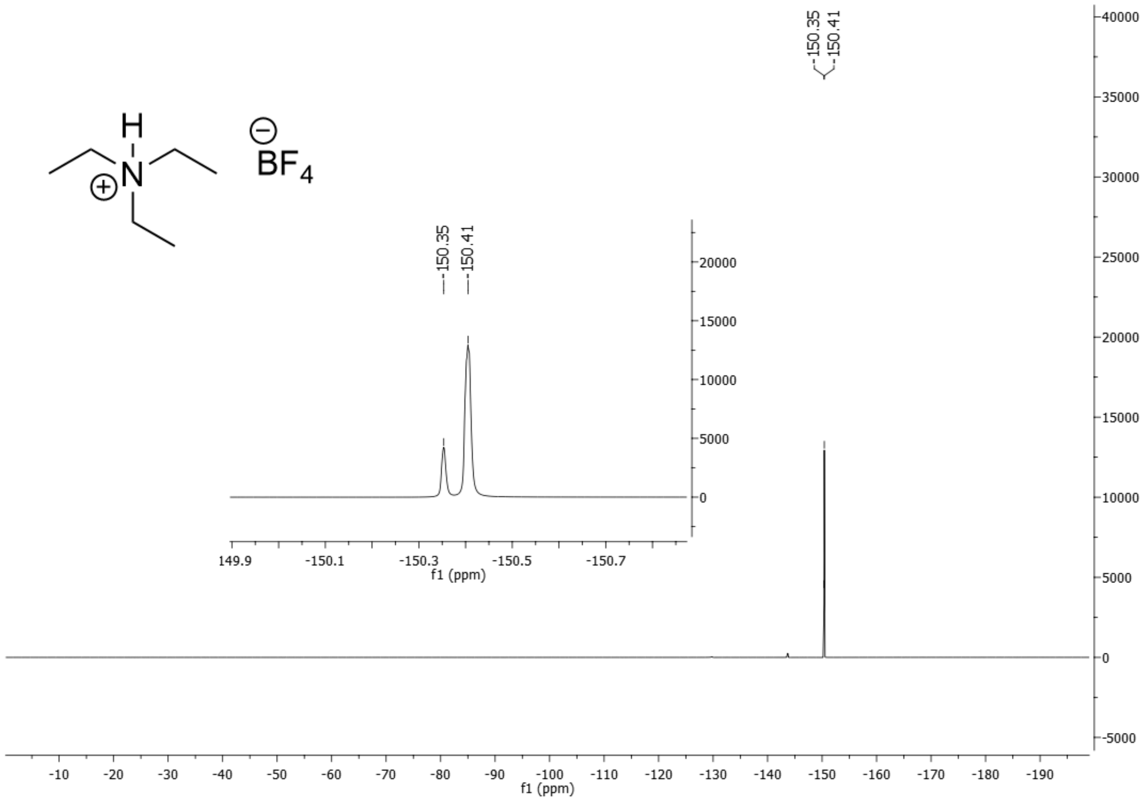

^{19}F NMR of compound **TEA-H·BF₄** in D_2O

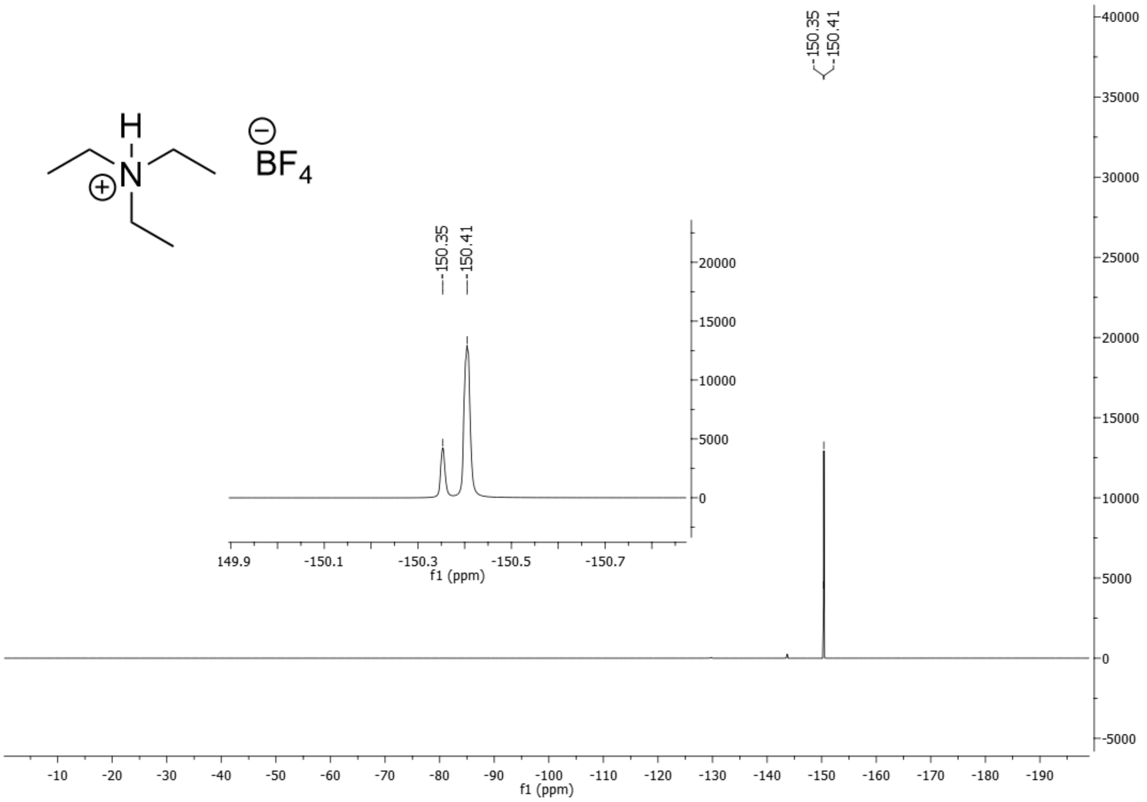

^{1}H NMR of compound **Py-H·BF₄** in D_2O

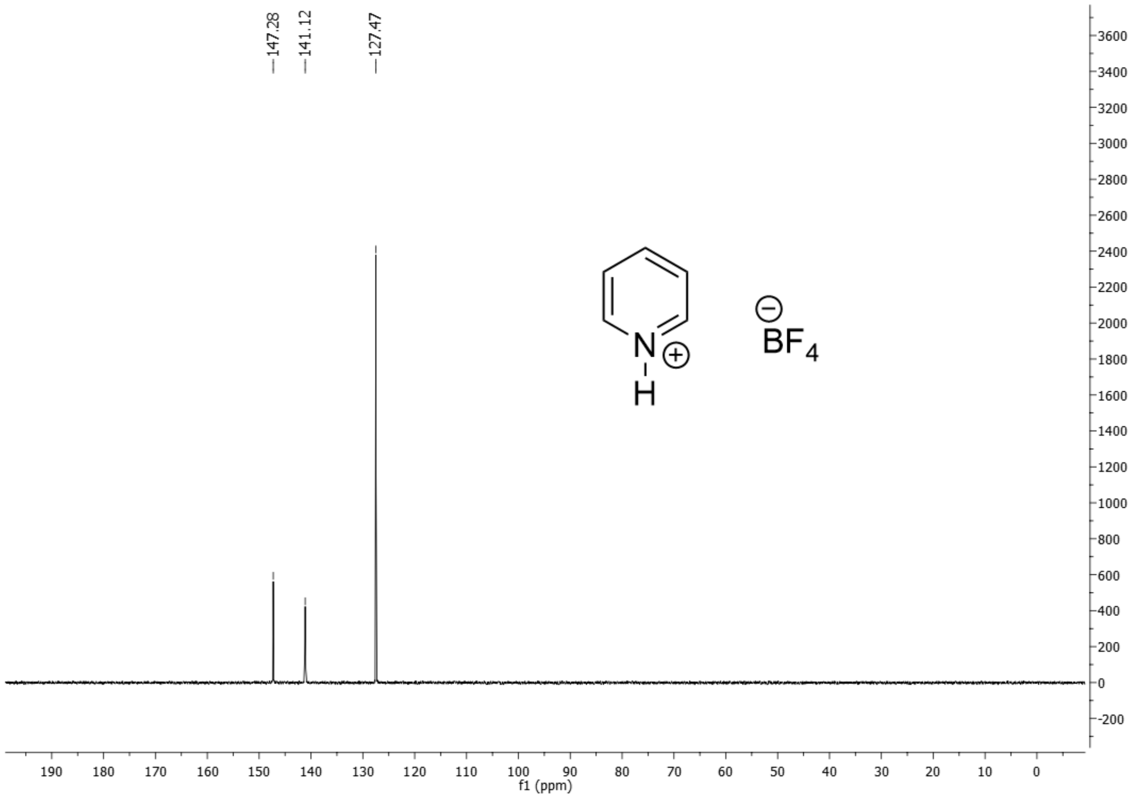

^{13}C NMR of compound **Py-H·BF₄** in D_2O

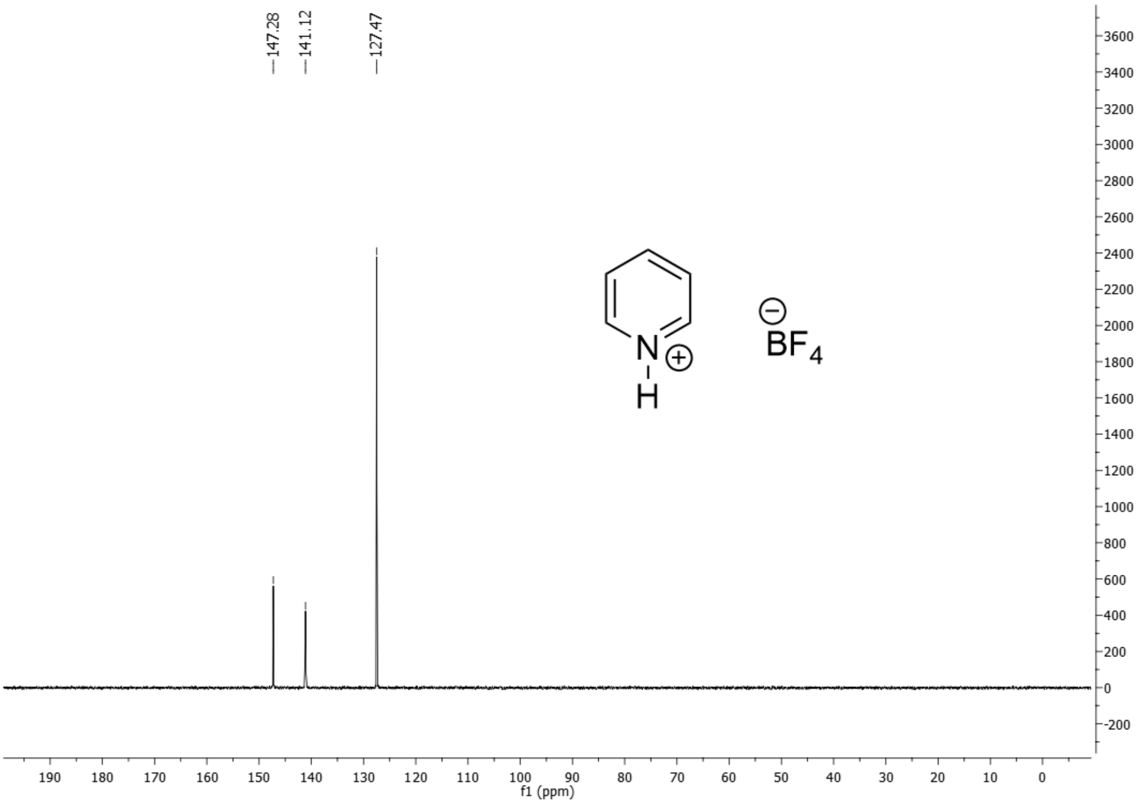

^{19}F NMR of compound **Py-H·BF₄** in D_2O

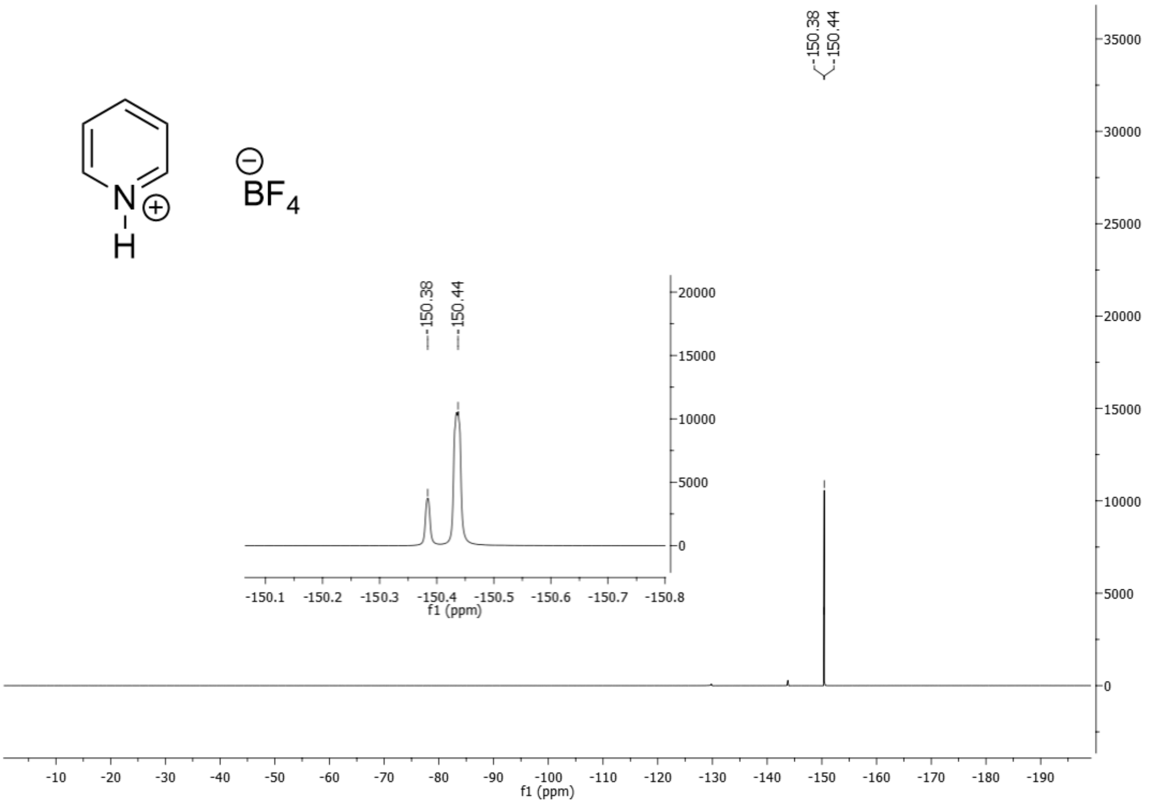

^{1}H NMR of compound **Imid-H·BF$_4$** in D_2O

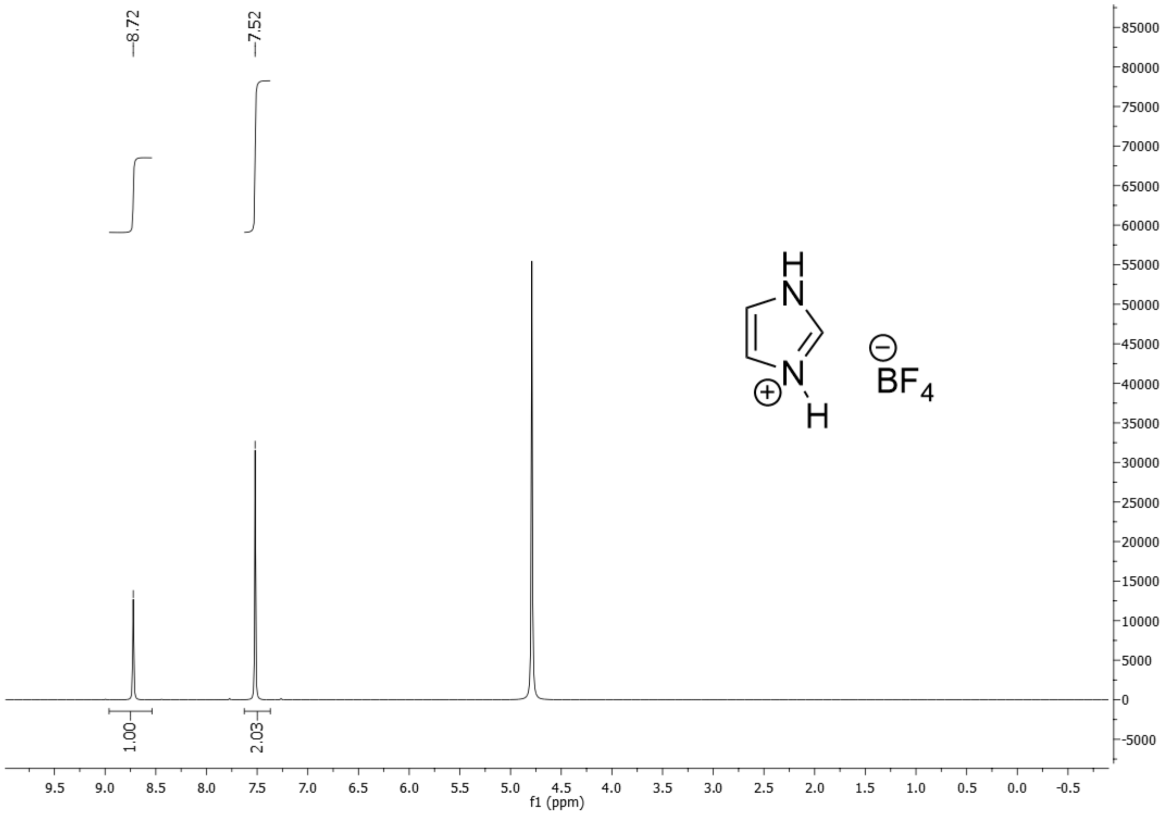

^{13}C NMR of compound **Imid-H·BF$_4$** in D_2O

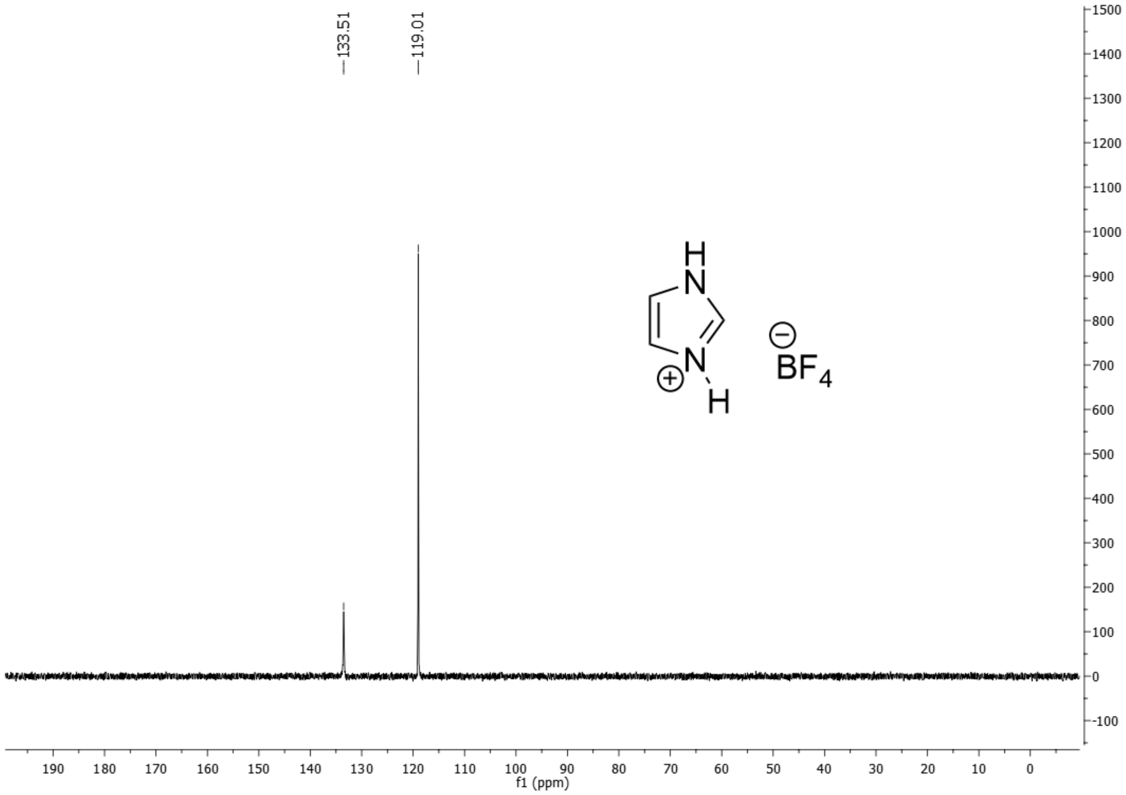

^{19}F NMR of compound **Imid-H·BF$_4$** in D_2O

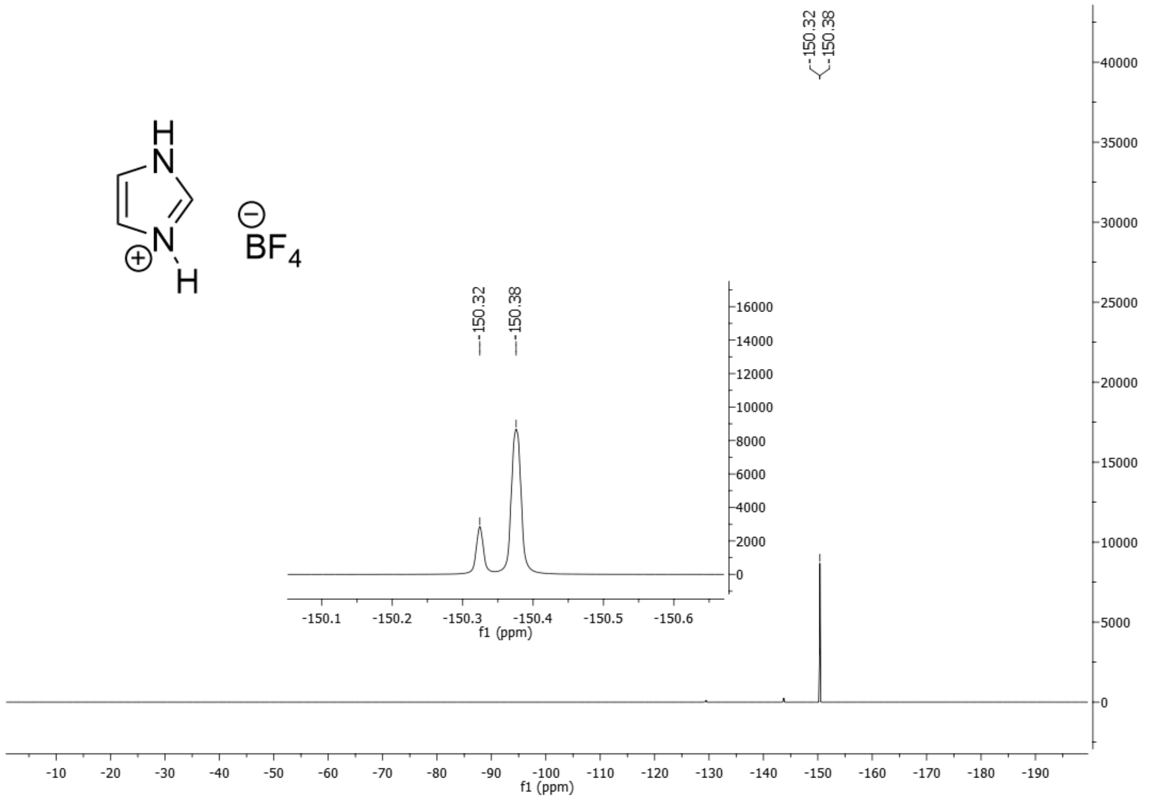

^{1}H NMR of compound **Anyl-H·BF$_4$** in D_2O

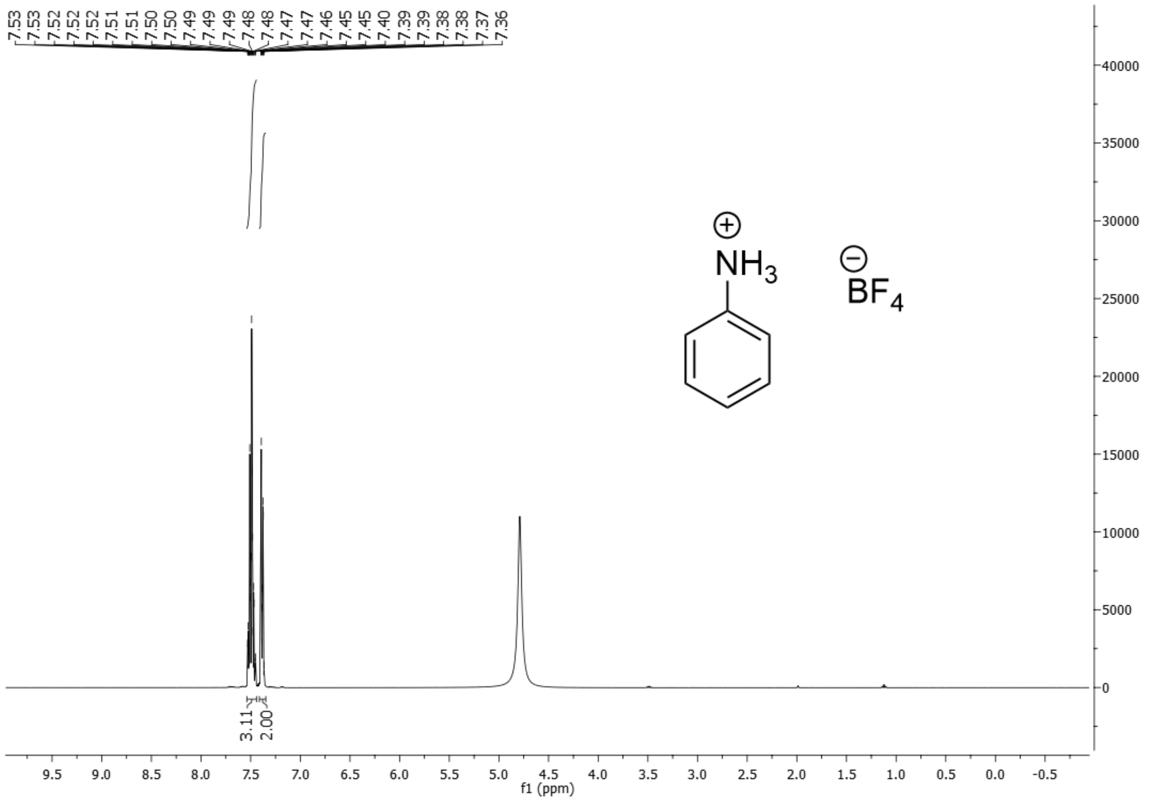

^{13}C NMR of compound **Anyl-H·BF₄** in D_2O

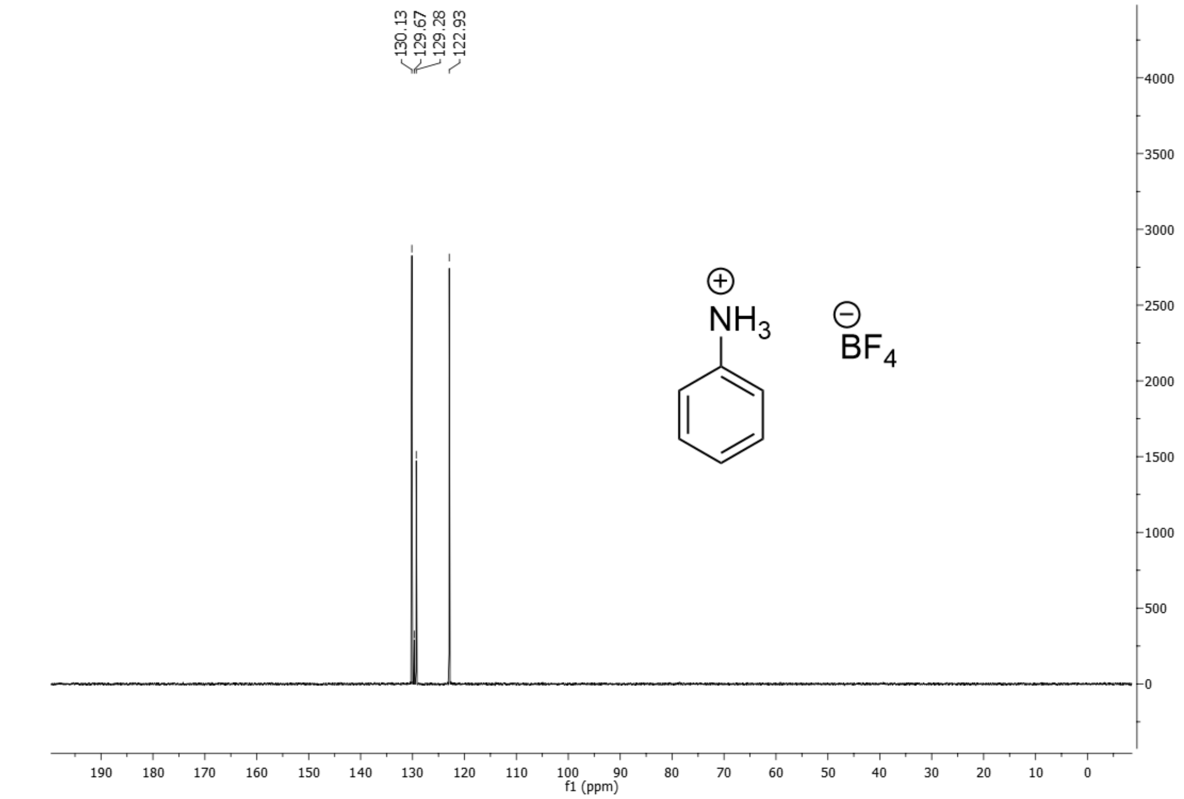

^{19}F NMR of compound **Anyl-H·BF₄** in D_2O

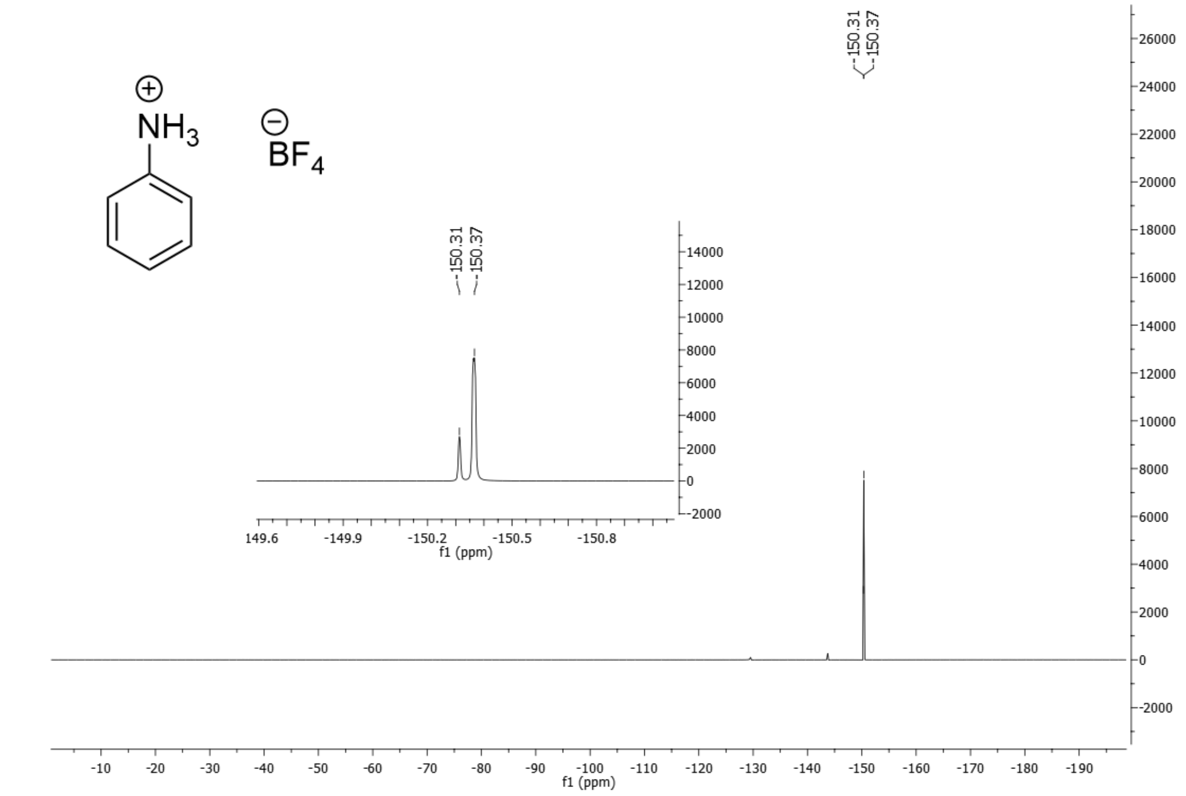

^{1}H NMR of compound **DMAP-H·BF₄** in D_2O

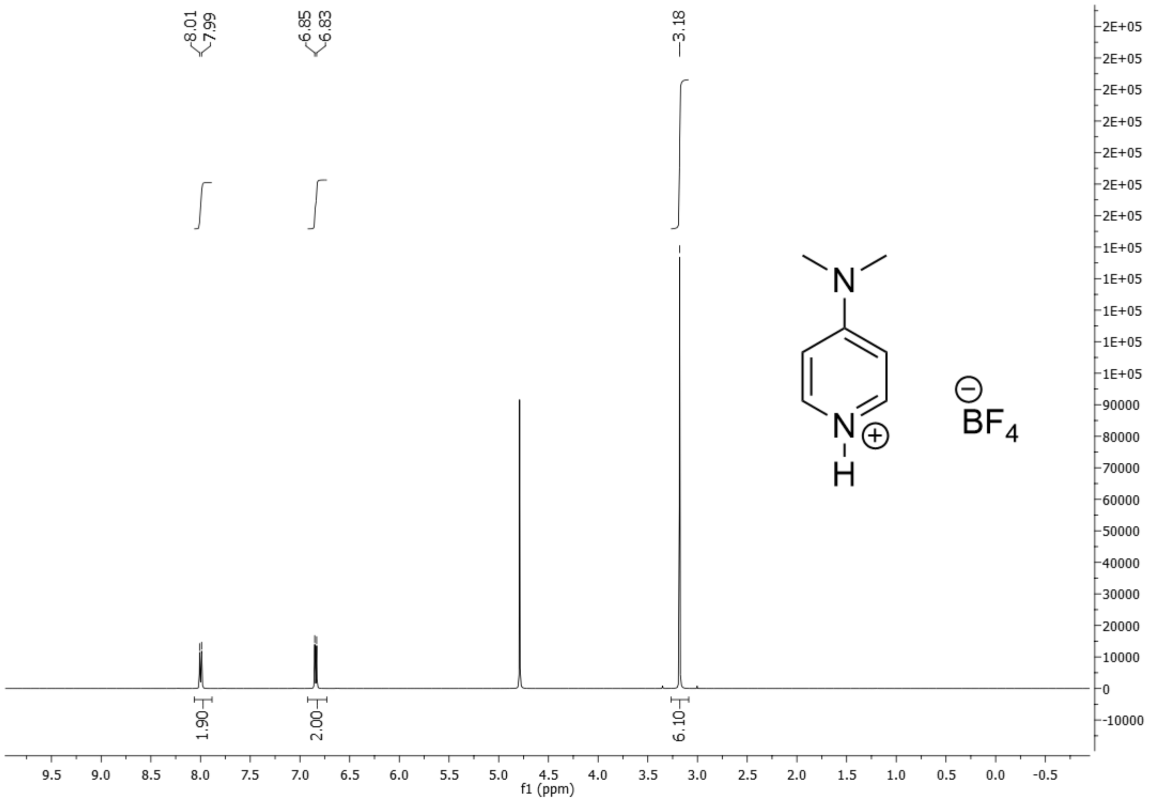

^{13}C NMR of compound **DMAP-H·BF₄** in D_2O

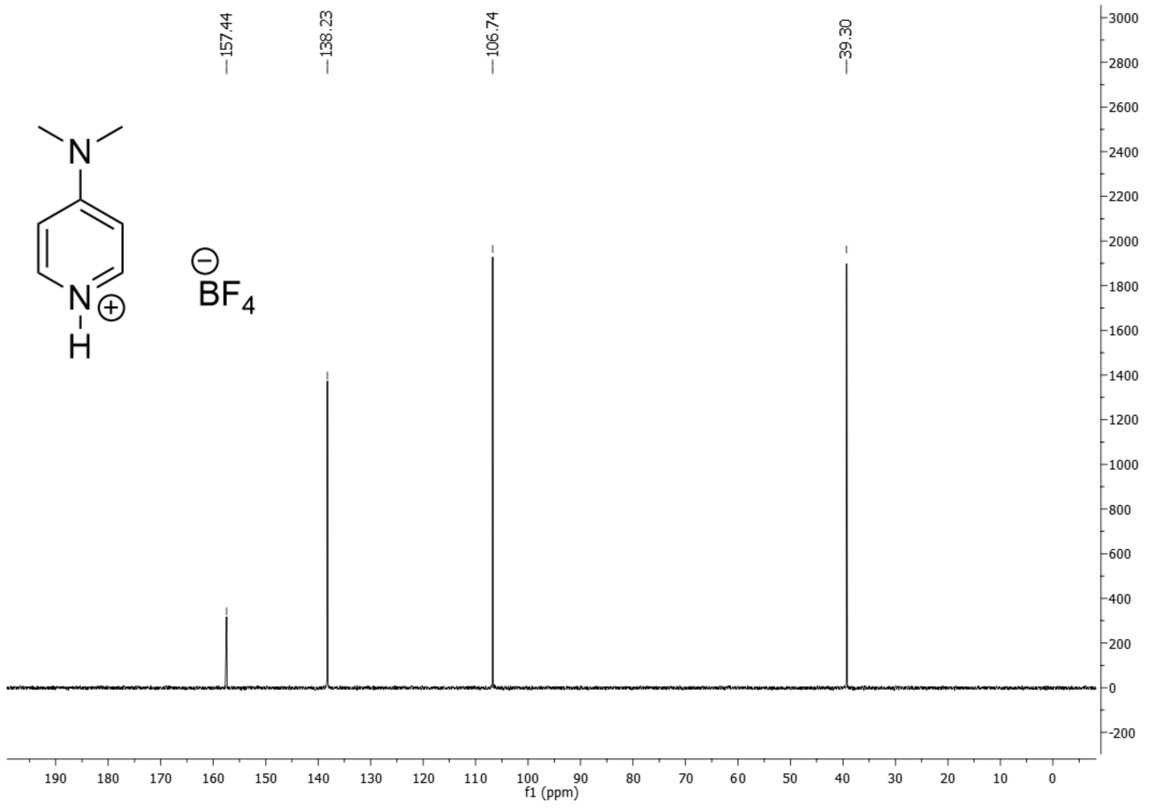

^{19}F NMR of compound **DMAP-H·BF₄** in D_2O

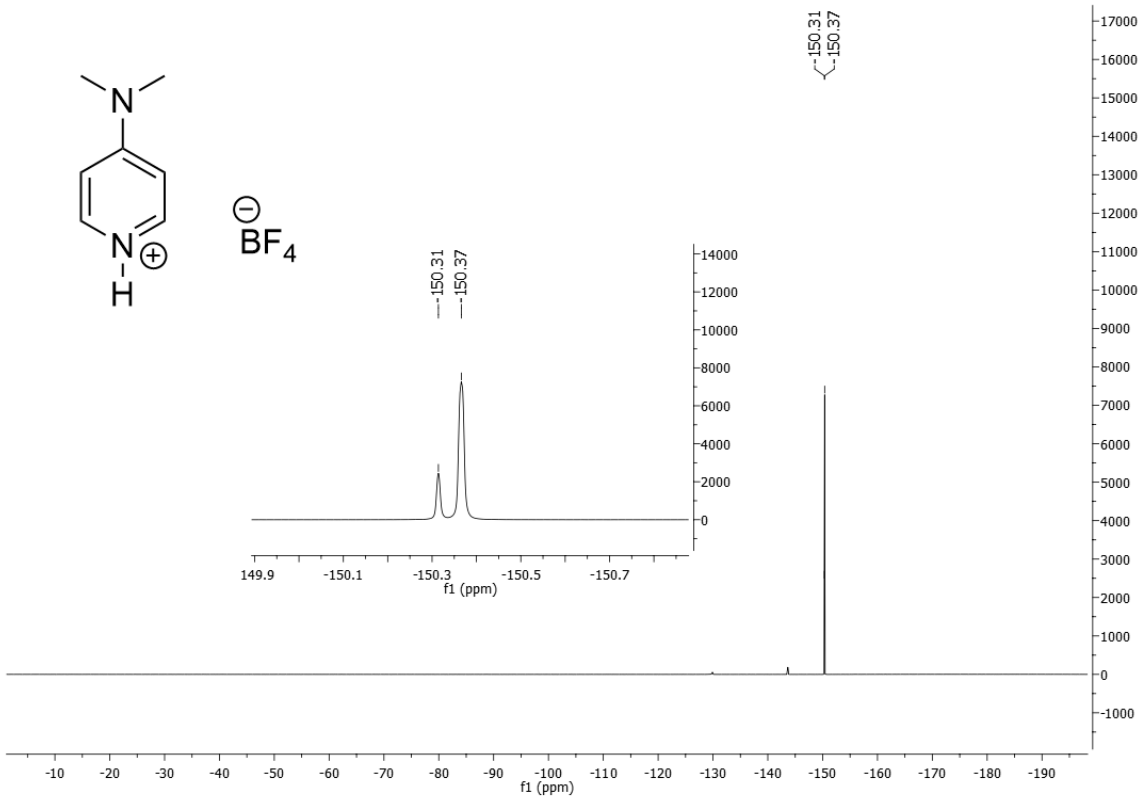

^{1}H NMR of compound **BnMIM-H·BF₄** in D_2O

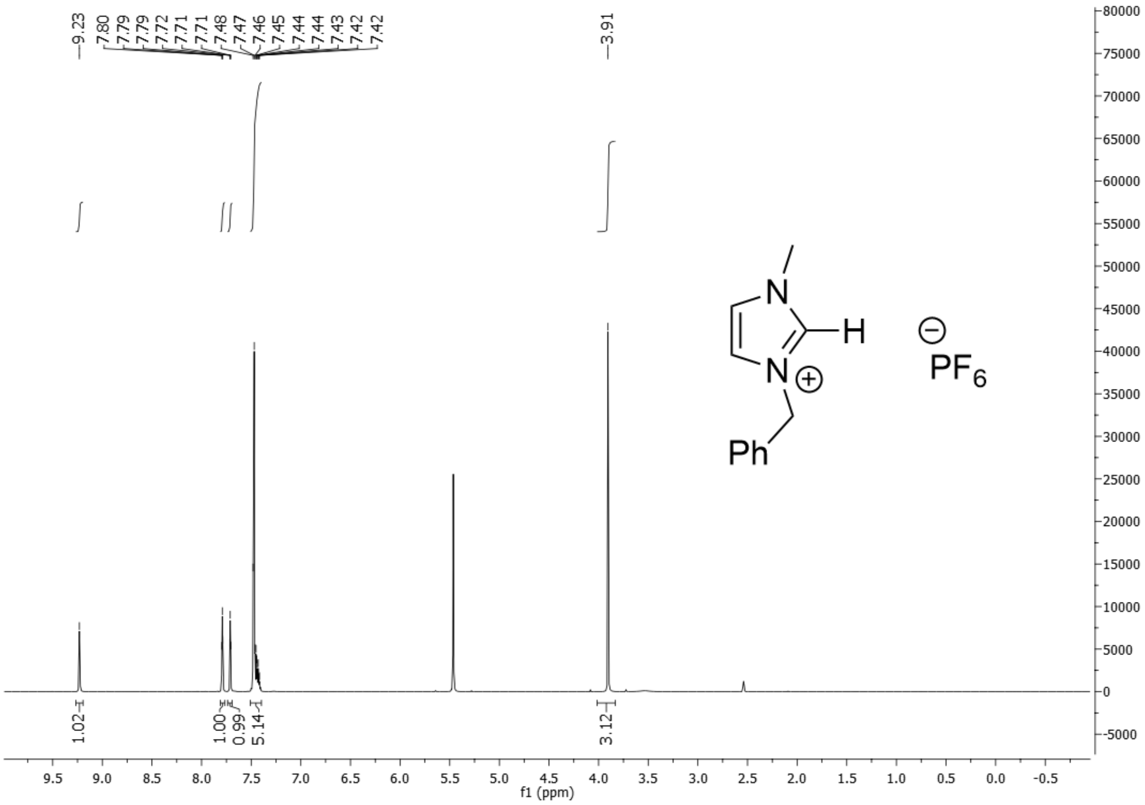

^{13}C NMR of compound **BnMIM-H·BF$_4$** in D_2O

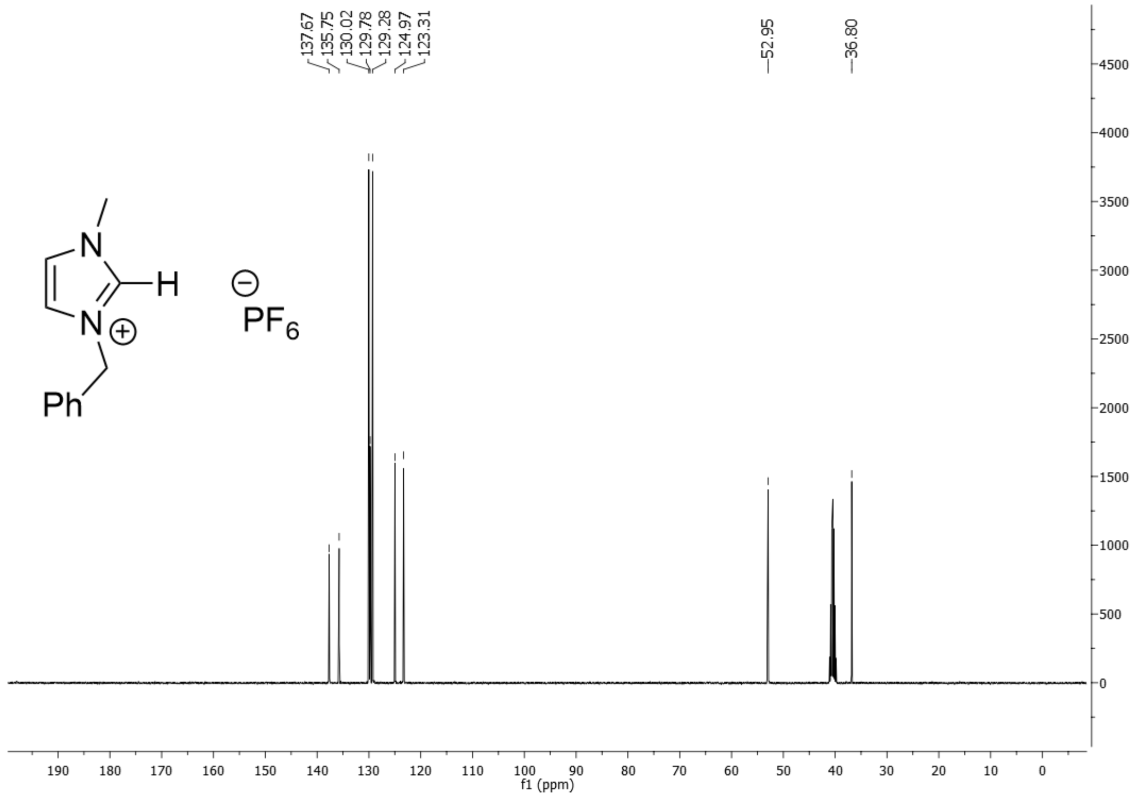

^{19}F NMR of compound **BnMIM-H·BF$_4$** in D_2O

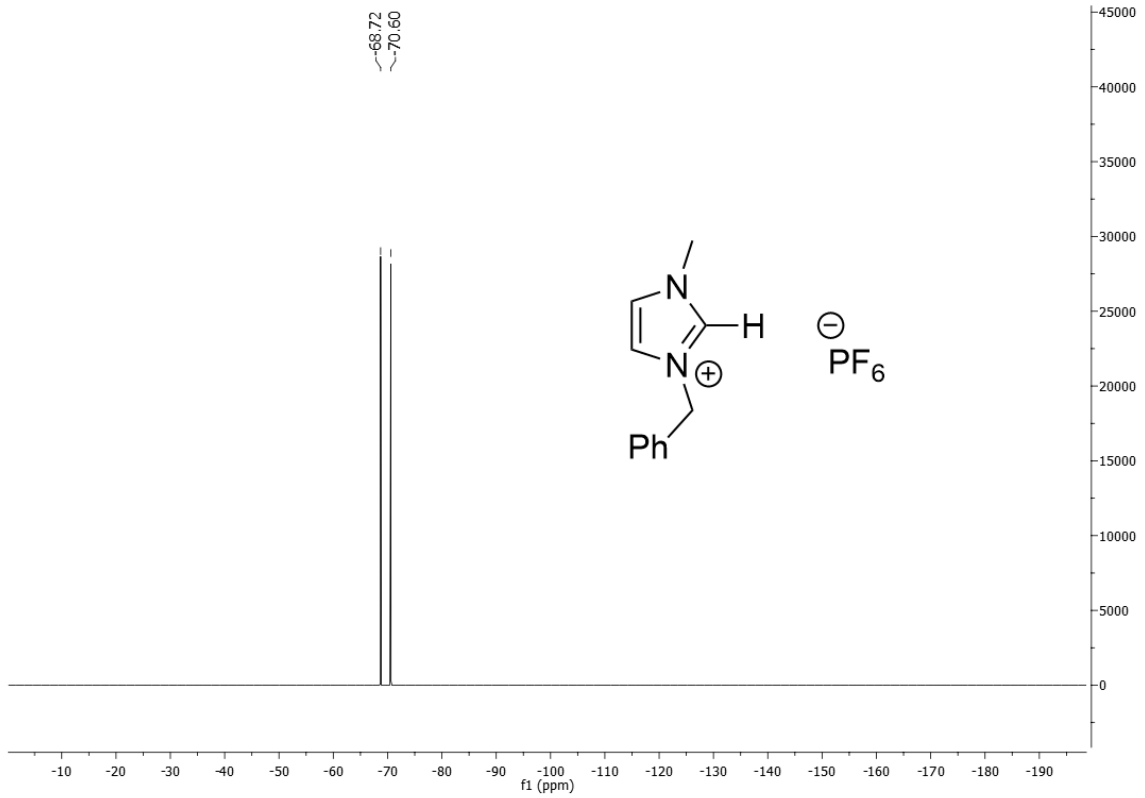

^{31}P NMR of compound **BnMIM-H·BF$_4$** in D$_2$O

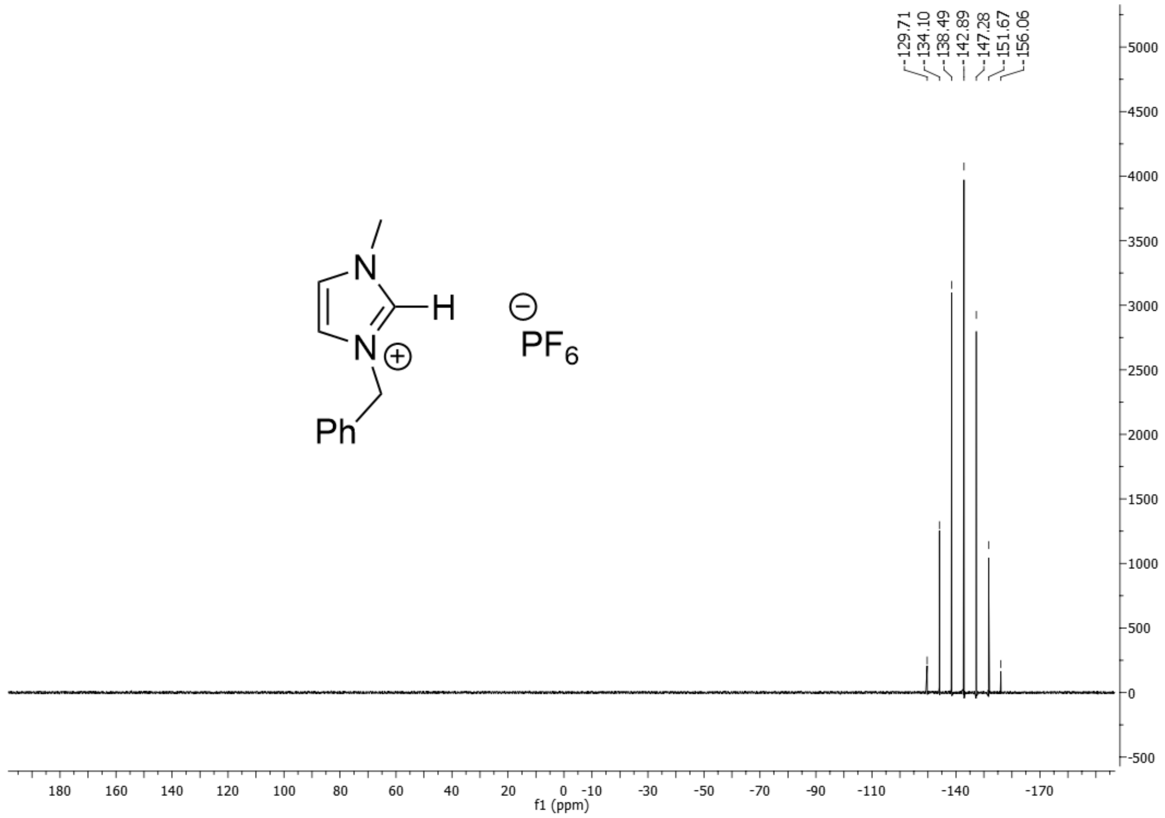

^{1}H NMR of compound **2k** in CDCl$_3$

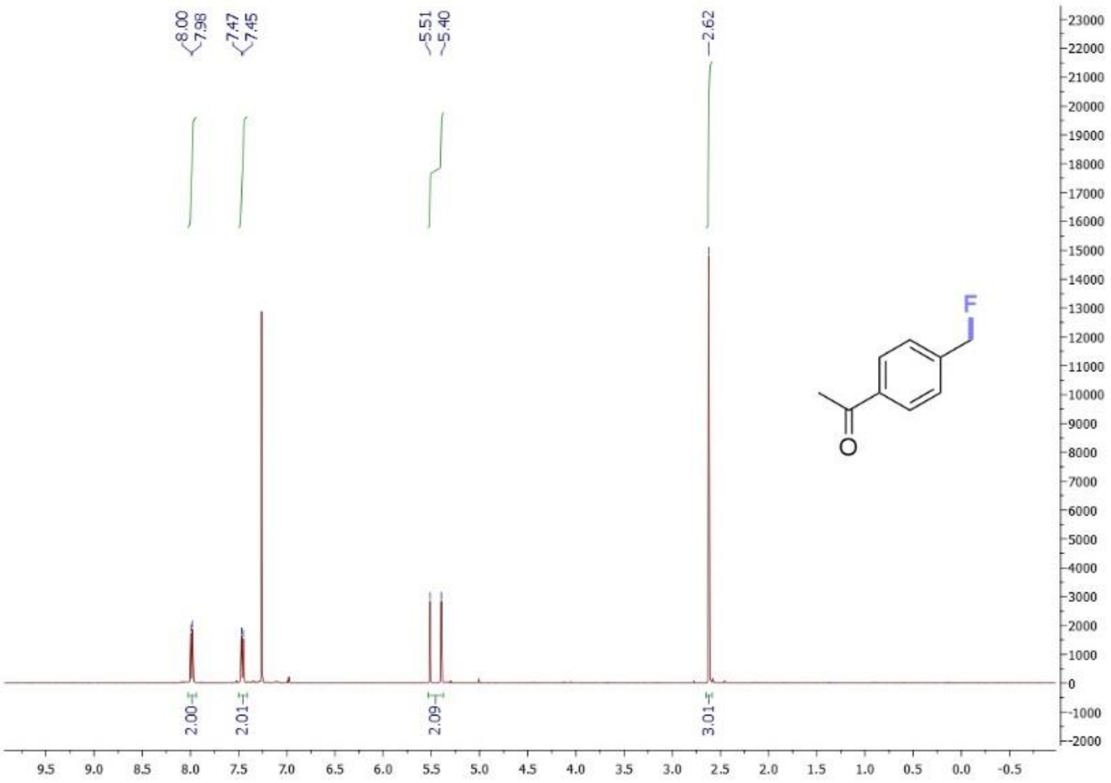

^{13}C NMR of compound **2k** in $CDCl_3$

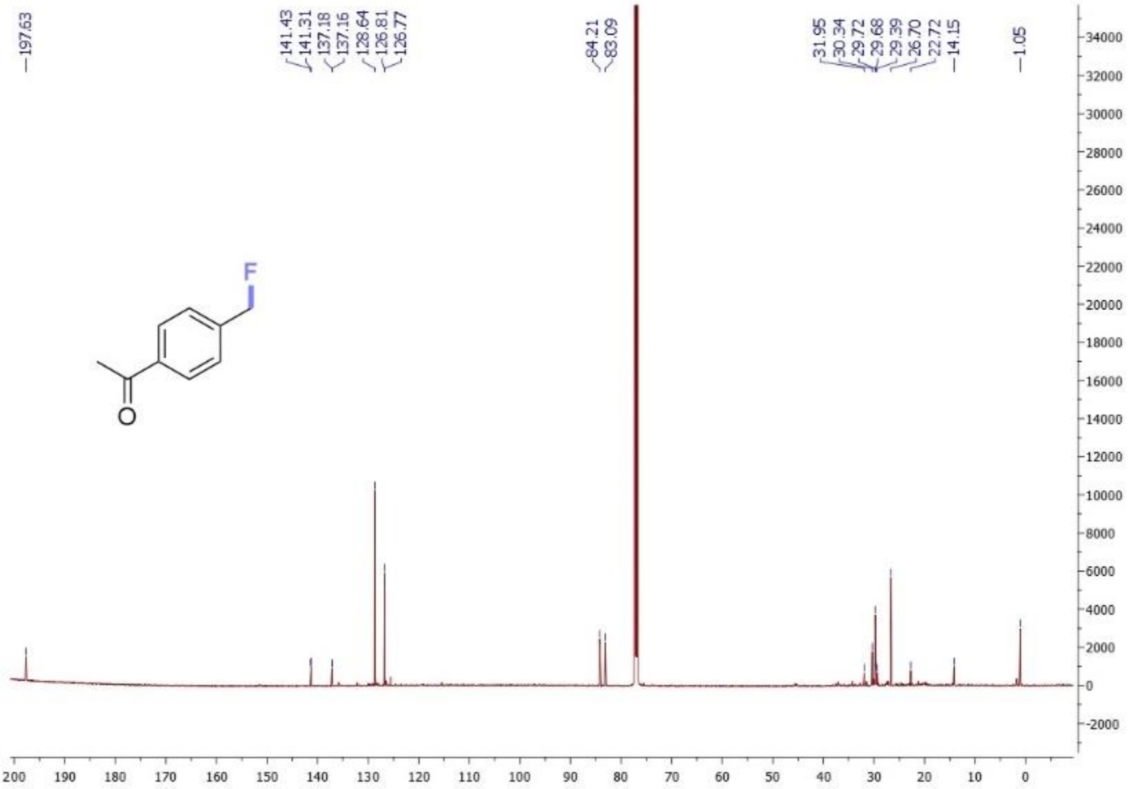

^{19}F NMR of compound **2k** in $CDCl_3$

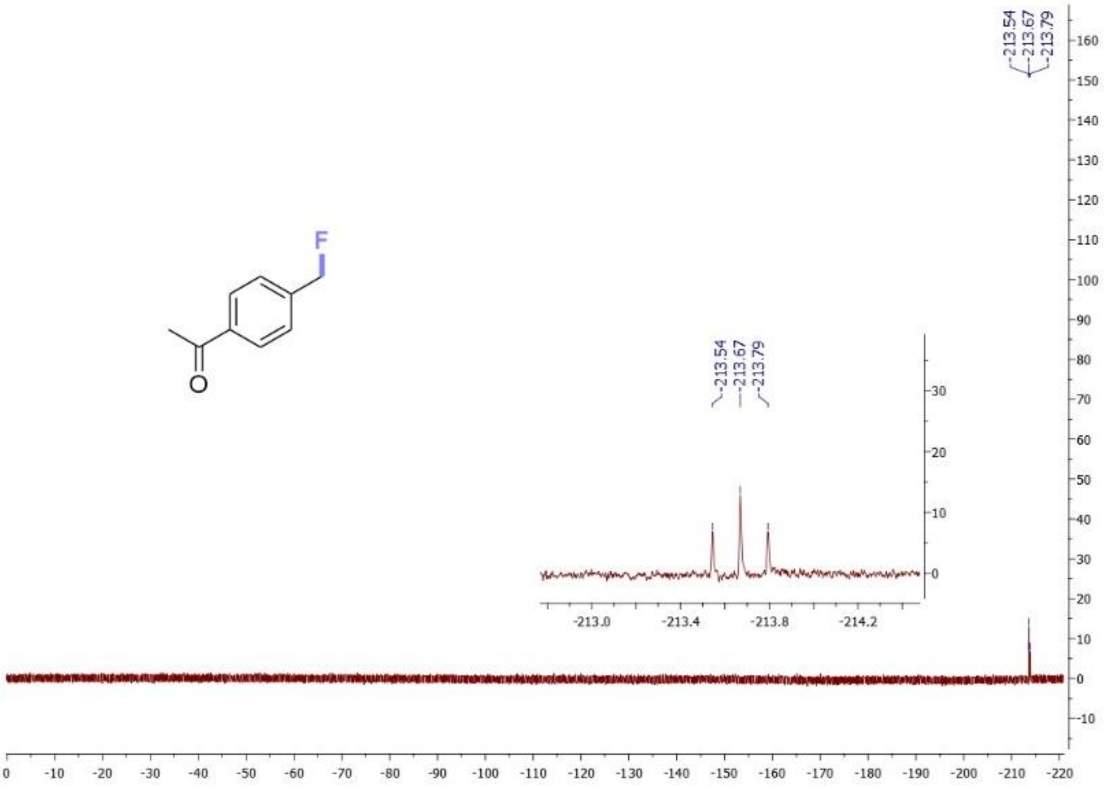

^{1}H NMR of compound **2d** in $CDCl_3$

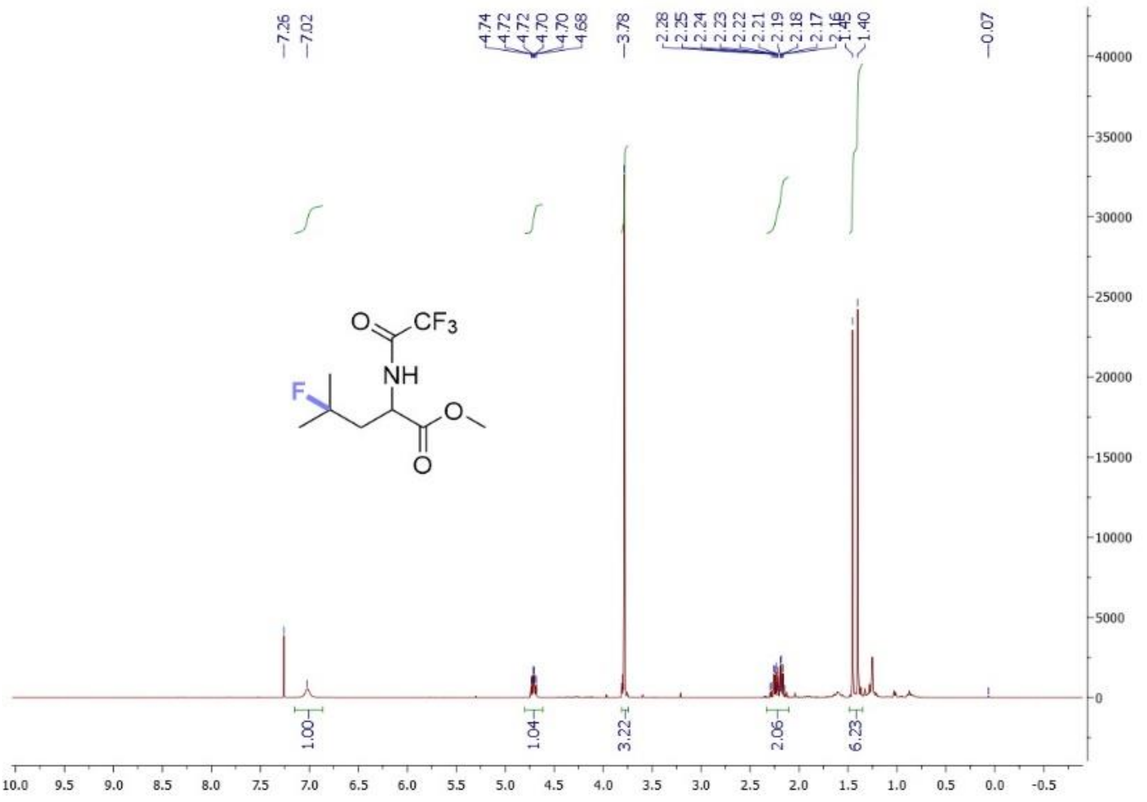

^{13}C NMR of compound **2d** in $CDCl_3$

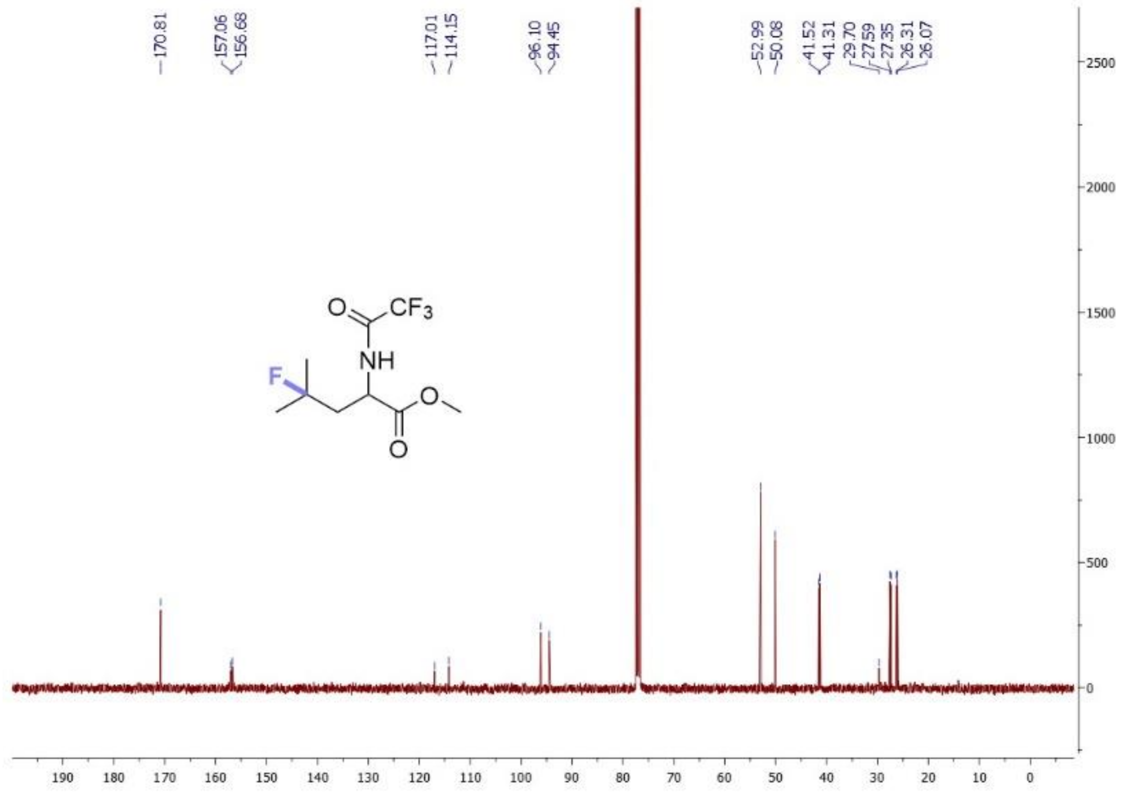

^{19}F NMR of compound **2d** in $CDCl_3$

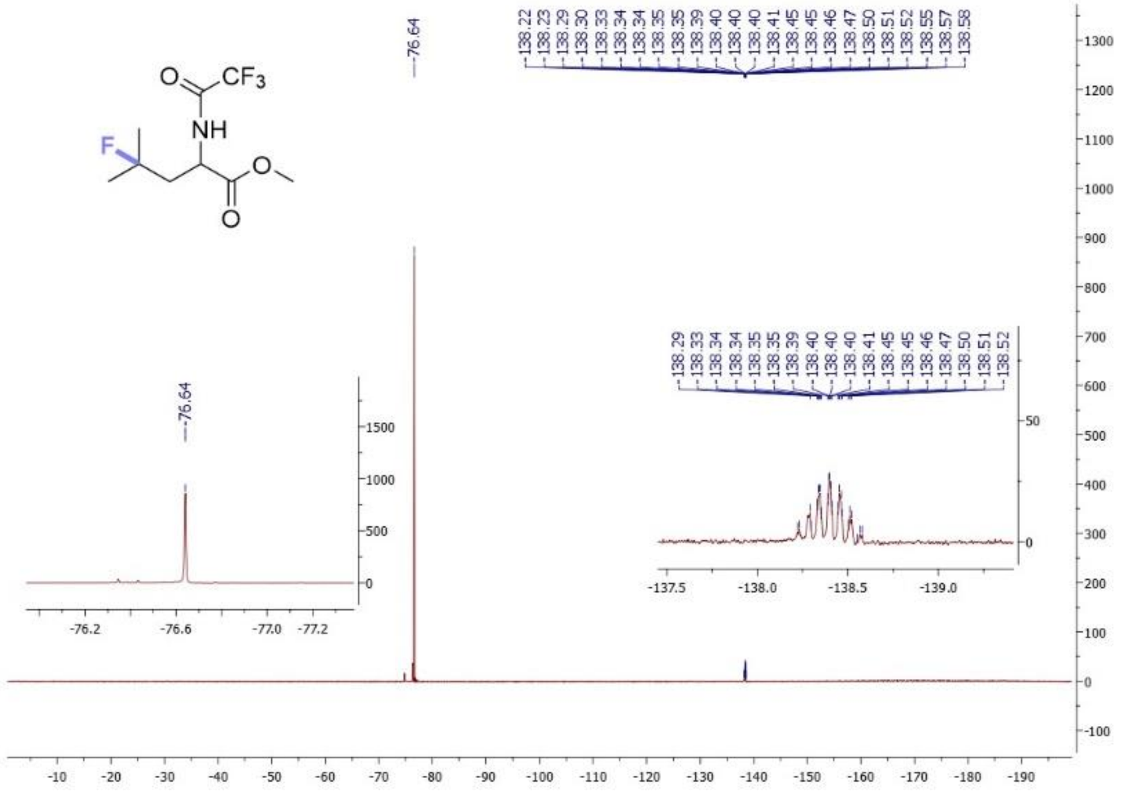

^{1}H NMR of compound **2i** in $CDCl_3$

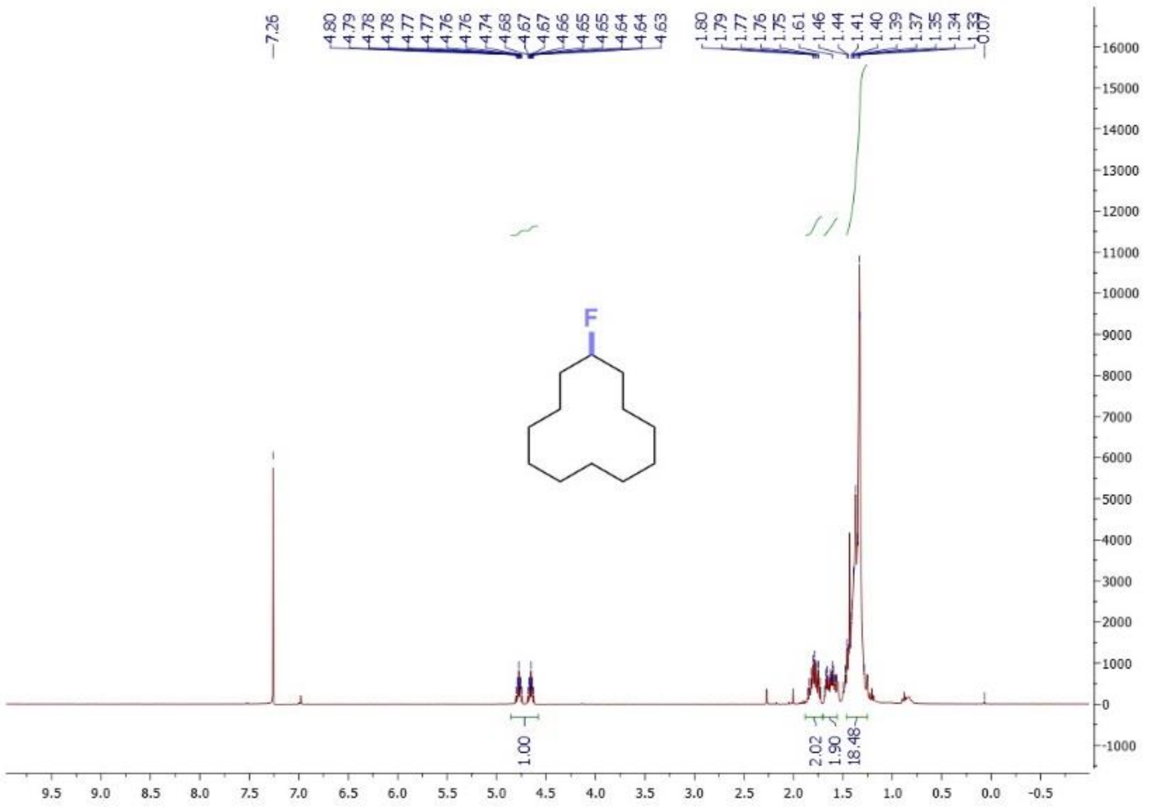

^{13}C NMR of compound **2i** in $CDCl_3$

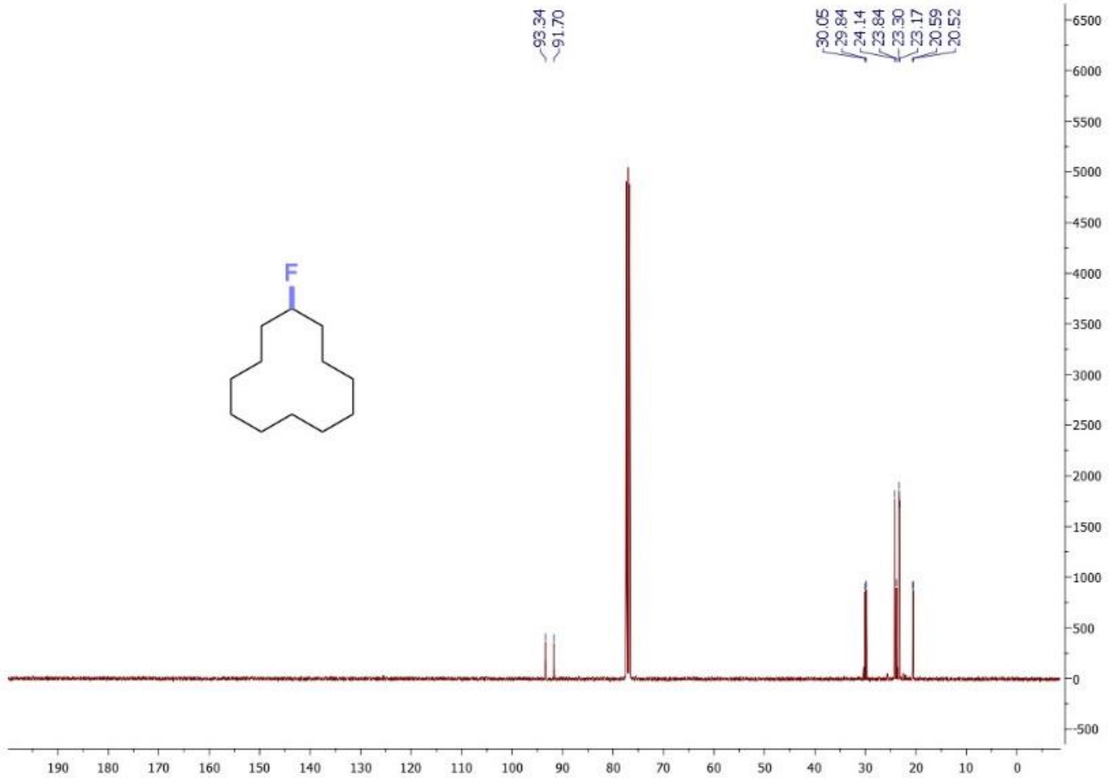

^{19}F NMR of compound **2i** in $CDCl_3$

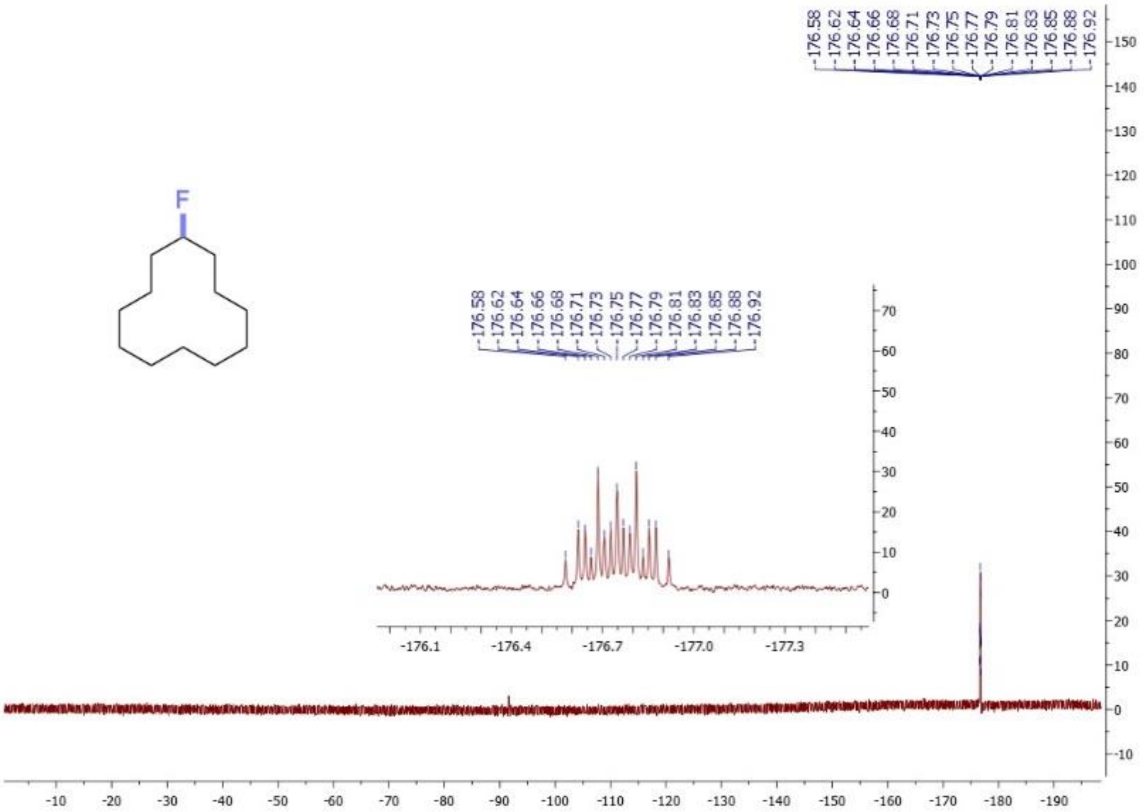

^{1}H NMR of compound **2g** in $CDCl_3$

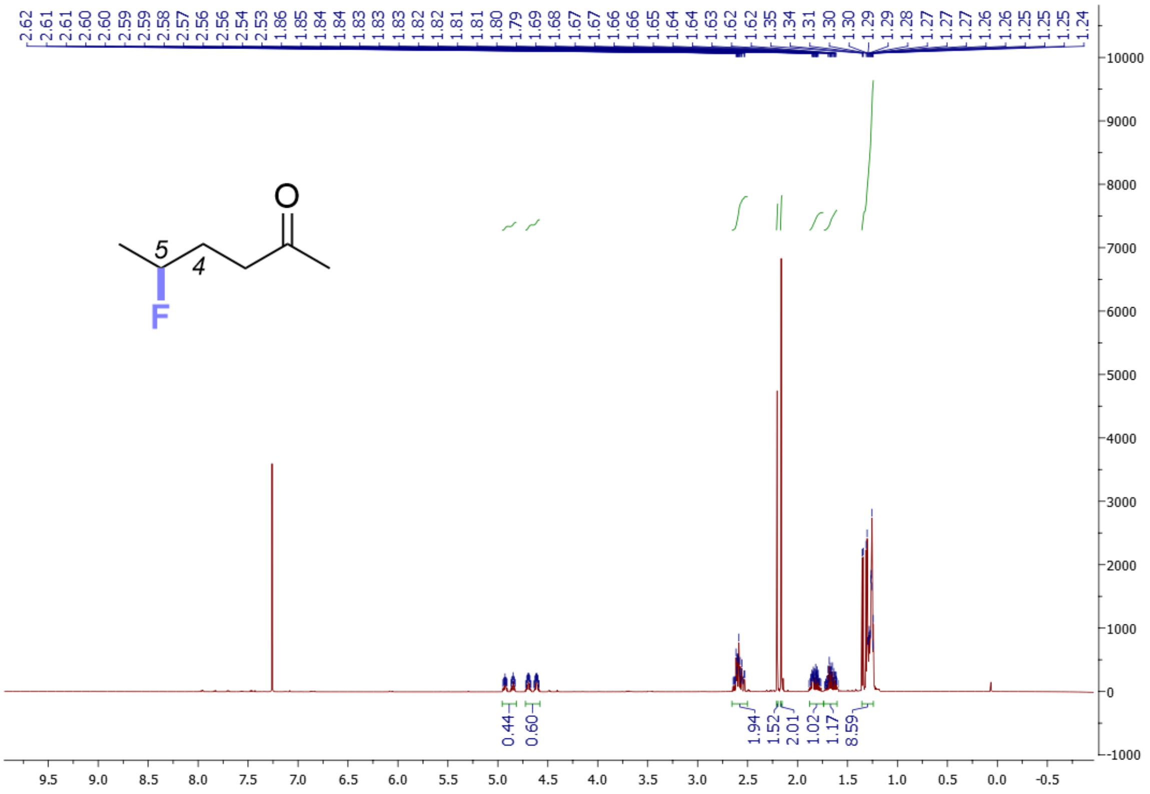

^{13}C NMR of compound **2g** in $CDCl_3$

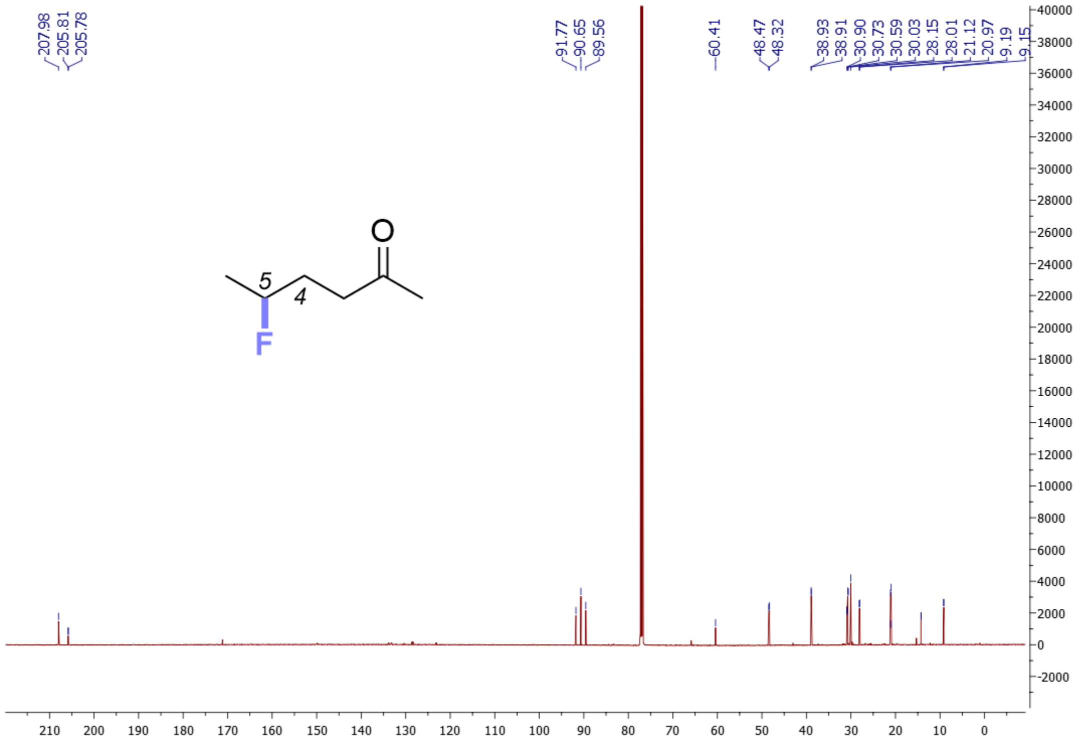

^{19}F NMR of compound **2g** in $CDCl_3$

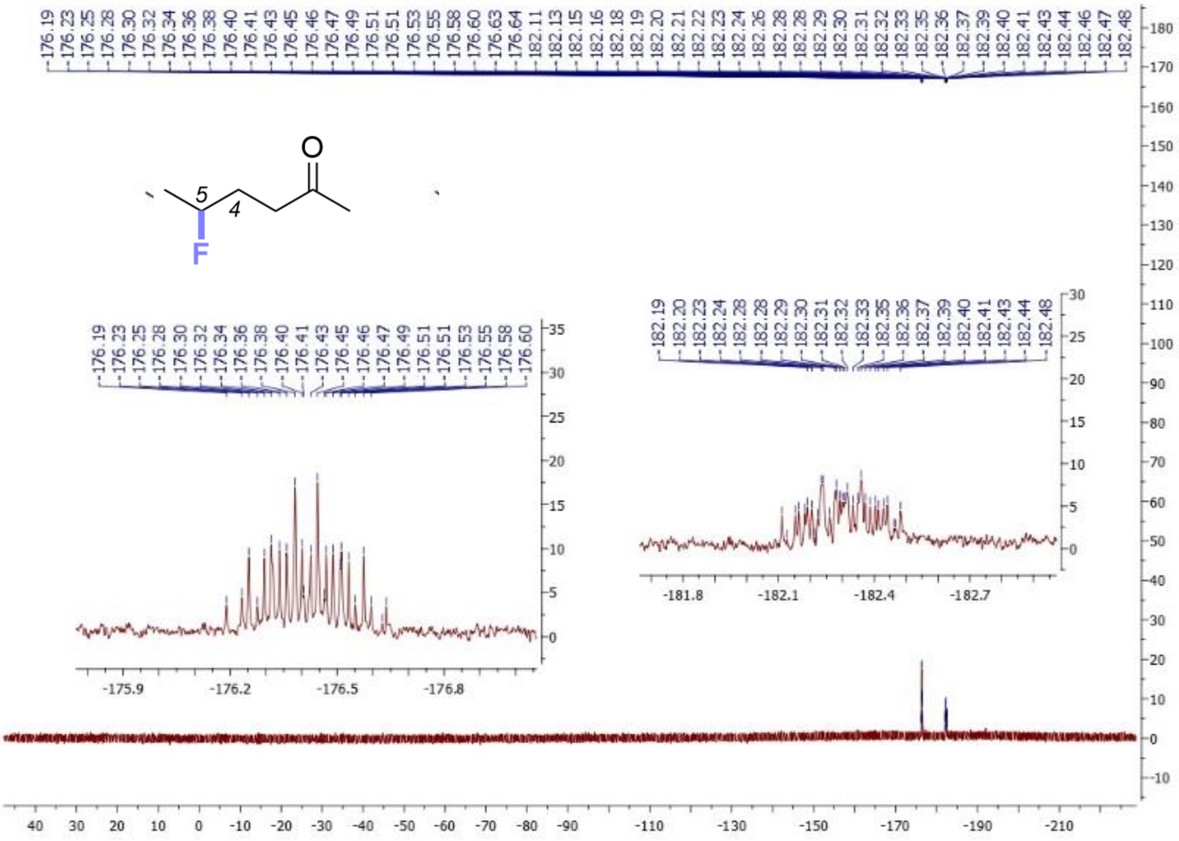

7 References

[1] S. Yakubov, W. J. Stockerl, X. Tian, A. Shahin, M. J. P. Mandigma, R. M. Gschwind, J. P. Barham, *Chem. Sci.* **2022**, *13*, 14041.

[2] A. M. Hua, D. N. Mai, R. Martinez, R. D. Baxter, *Org. Lett.* **2017**, *19*, 2949.

[3] S. Bloom, J. L. Knippel, T. Lectka, *Chem. Sci.* **2014**, *5*, 1175.

[4] J. B. Xia, C. Zhu, C. Chen, *Chem. Commun.* **2014**, *50*, 11701.

[5] C. W. Kee, K. F. Chin, M. W. Wong, C. H. Tan, *Chem. Commun.* **2014**, *50*, 8211.

[6] Y. F. Liang, N. Jiao, *Angew. Chem. Int. Ed.* **2014**, 53, 548.

[7] N. R. Paz, D. Rodríguez-Sosa, H. Valdes, R. Marticorena, D. Melian, M. B. Copano, C. C. Gonzáles, A. J. Herrera, *Org. Lett.* **2015**, 17, 2370.

[8] J. B. Xia, Y. Ma, C. Chen, *Org. Chem. Front.* **2014**, *1*, 468.

[9] C. Feldmeier, H. Bartling, E. Riedle, R. M. Gschwind, *J. Magn. Reson.* **2013**, *232*, 39.

[10] A. Jerschow, N. Müller, *J. Mag. Res*. **1997**, *125*, 372.

[11] E. O. Stejskal, J. E. Tanner, *J. Chem. Phys.* **1965**, *42*, 288.

[12] D. Zuccaccia, A. Macchioni, *Organometallics* **2005**, *24*, 3476.

[13] H. C. Chen, S. H. Chen, *J. Phys. Chem.* **1984**, *88*, 5118.

[14] T. D. W. Claridge, *High-Resolution NMR Techniques in Organic Chemistry* **2009**, 38-4
